# Low-frequency and rare exome chip variants associate with fasting glucose and type 2 diabetes susceptibility

**DOI:** 10.1038/ncomms6897

**Published:** 2015-01-29

**Authors:** Jennifer Wessel, Audrey Y Chu, Sara M Willems, Shuai Wang, Hanieh Yaghootkar, Jennifer A Brody, Marco Dauriz, Marie-France Hivert, Sridharan Raghavan, Leonard Lipovich, Bertha Hidalgo, Keolu Fox, Jennifer E Huffman, Ping An, Yingchang Lu, Laura J Rasmussen-Torvik, Niels Grarup, Margaret G Ehm, Li Li, Abigail S Baldridge, Alena Stančáková, Ravinder Abrol, Céline Besse, Anne Boland, Jette Bork-Jensen, Myriam Fornage, Daniel F Freitag, Melissa E Garcia, Xiuqing Guo, Kazuo Hara, Aaron Isaacs, Johanna Jakobsdottir, Leslie A Lange, Jill C Layton, Man Li, Jing Hua Zhao, Karina Meidtner, Alanna C Morrison, Mike A Nalls, Marjolein J Peters, Maria Sabater-Lleal, Claudia Schurmann, Angela Silveira, Albert V Smith, Lorraine Southam, Marcus H Stoiber, Rona J Strawbridge, Kent D Taylor, Tibor V Varga, Kristine H Allin, Najaf Amin, Jennifer L Aponte, Tin Aung, Caterina Barbieri, Nathan A Bihlmeyer, Michael Boehnke, Cristina Bombieri, Donald W Bowden, Sean M Burns, Yuning Chen, Yii-DerI Chen, Ching-Yu Cheng, Adolfo Correa, Jacek Czajkowski, Abbas Dehghan, Georg B Ehret, Gudny Eiriksdottir, Stefan A Escher, Aliki-Eleni Farmaki, Mattias Frånberg, Giovanni Gambaro, Franco Giulianini, William A Goddard, Anuj Goel, Omri Gottesman, Megan L Grove, Stefan Gustafsson, Yang Hai, Göran Hallmans, Jiyoung Heo, Per Hoffmann, Mohammad K Ikram, Richard A Jensen, Marit E Jørgensen, Torben Jørgensen, Maria Karaleftheri, Chiea C Khor, Andrea Kirkpatrick, Aldi T Kraja, Johanna Kuusisto, Ethan M Lange, I T Lee, Wen-Jane Lee, Aaron Leong, Jiemin Liao, Chunyu Liu, Yongmei Liu, Cecilia M Lindgren, Allan Linneberg, Giovanni Malerba, Vasiliki Mamakou, Eirini Marouli, Nisa M Maruthur, Angela Matchan, Roberta McKean-Cowdin, Olga McLeod, Ginger A Metcalf, Karen L Mohlke, Donna M Muzny, Ioanna Ntalla, Nicholette D Palmer, Dorota Pasko, Andreas Peter, Nigel W Rayner, Frida Renström, Ken Rice, Cinzia F Sala, Bengt Sennblad, Ioannis Serafetinidis, Jennifer A Smith, Nicole Soranzo, Elizabeth K Speliotes, Eli A Stahl, Kathleen Stirrups, Nikos Tentolouris, Anastasia Thanopoulou, Mina Torres, Michela Traglia, Emmanouil Tsafantakis, Sundas Javad, Lisa R Yanek, Eleni Zengini, Diane M Becker, Joshua C Bis, James B Brown, L Adrienne Cupples, Torben Hansen, Erik Ingelsson, Andrew J Karter, Carlos Lorenzo, Rasika A Mathias, Jill M Norris, Gina M Peloso, Wayne H.-H. Sheu, Daniela Toniolo, Dhananjay Vaidya, Rohit Varma, Lynne E Wagenknecht, Heiner Boeing, Erwin P Bottinger, George Dedoussis, Panos Deloukas, Ele Ferrannini, Oscar H Franco, Paul W Franks, Richard A Gibbs, Vilmundur Gudnason, Anders Hamsten, Tamara B Harris, Andrew T Hattersley, Caroline Hayward, Albert Hofman, Jan-Håkan Jansson, Claudia Langenberg, Lenore J Launer, Daniel Levy, Ben A Oostra, Christopher J O’Donnell, Stephen O’Rahilly, Sandosh Padmanabhan, James S Pankow, Ozren Polasek, Michael A Province, Stephen S Rich, Paul M Ridker, Igor Rudan, Matthias B Schulze, Blair H Smith, André G Uitterlinden, Mark Walker, Hugh Watkins, Tien Y Wong, Eleftheria Zeggini, The EPIC-InterAct Consortium, Stephen J Sharp, Nita G Forouhi, Nicola D Kerrison, Debora ME Lucarelli, Matt Sims, Inês Barroso, Mark I McCarthy, Larraitz Arriola, Beverley Balkau, Aurelio Barricarte, Carlos Gonzalez, Sara Grioni, Rudolf Kaaks, Timothy J Key, Carmen Navarro, Peter M Nilsson, Kim Overvad, Domenico Palli, Salvatore Panico, J. Ramón Quirós, Olov Rolandsson, Carlotta Sacerdote, María–José Sánchez, Nadia Slimani, Anne Tjonneland, Rosario Tumino, Daphne L van der A, Yvonne T van der Schouw, Elio Riboli, Markku Laakso, Ingrid B Borecki, Daniel I Chasman, Oluf Pedersen, Bruce M Psaty, E Shyong Tai, Cornelia M van Duijn, Nicholas J Wareham, Dawn M Waterworth, Eric Boerwinkle, W H Linda Kao, Jose C Florez, Ruth J.F. Loos, James G Wilson, Timothy M Frayling, David S Siscovick, Josée Dupuis, Jerome I Rotter, James B Meigs, Robert A Scott, Mark O Goodarzi

**Affiliations:** 1 Department of Epidemiology, Fairbanks School of Public Health, Indianapolis, Indiana 46202, USA; 2 Department of Medicine, Indiana University School of Medicine, Indianapolis, Indiana 46202, USA; 3 Division of Preventive Medicine, Brigham and Women’s Hospital, Boston, Massachusetts 02215, USA; 4 National Heart, Lung, and Blood Institute (NHLBI) Framingham Heart Study, Framingham, Massachusetts 01702, USA; 5 Genetic Epidemiology Unit, Department of Epidemiology, Erasmus University Medical Center, Rotterdam 3000 CE, The Netherlands; 6 MRC Epidemiology Unit, University of Cambridge School of Clinical Medicine, Institute of Metabolic Science, Cambridge Biomedical Campus, Cambridge CB2 0SL, UK; 7 Department of Biostatistics, Boston University School of Public Health, Boston, Massachusetts 02118, USA; 8 Genetics of Complex Traits, University of Exeter Medical School, University of Exeter, Exeter EX1 2LU, UK; 9 Cardiovascular Health Research Unit, University of Washington, Seattle, Washington 98101, USA; 10 Department of Medicine, University of Washington, Seattle, Washington 98195, USA; 11 Massachusetts General Hospital, General Medicine Division, Boston, Massachusetts 02114, USA; 12 Department of Medicine, Harvard Medical School, Boston, Massachusetts 02115, USA; 13 Division of Endocrinology, Diabetes and Metabolism, Department of Medicine, University of Verona Medical School and Hospital Trust of Verona, Verona 37126, Italy; 14 Harvard Pilgrim Health Care Institute, Department of Population Medicine, Harvard Medical School, Boston, Massachusetts 02215, USA; 15 Division of Endocrinology and Metabolism, Department of Medicine, Université de Sherbrooke, Sherbrooke, Québec, Canada J1K 2R1; 16 Diabetes Unit, Department of Medicine, Massachusetts General Hospital, Boston, Massachusetts 02114, USA; 17 Center for Molecular Medicine and Genetics, Wayne State University, Detroit, Michigan 48201, USA; 18 Department of Neurology, Wayne State University School of Medicine, Detroit, Michigan 48202, USA; 19 Department of Epidemiology, University of Alabama at Birmingham, Birmingham, Alabama 35233, USA; 20 Department of Genome Sciences, University of Washington, Seattle, Washington 98195, USA; 21 MRC Human Genetics Unit, MRC IGMM, University of Edinburgh, Edinburgh, Scotland EH4 2XU, UK; 22 Division of Statistical Genomics and Department of Genetics, Washington University School of Medicine, St. Louis, Missouri 63108, USA; 23 The Charles Bronfman Institute for Personalized Medicine, The Icahn School of Medicine at Mount Sinai, New York, New York 10029, USA; 24 The Genetics of Obesity and Related Metabolic Traits Program, The Icahn School of Medicine at Mount Sinai, New York, New York 10029, USA; 25 Department of Preventive Medicine, Northwestern University Feinberg School of Medicine, Chicago, Illinois 60611, USA; 26 The Novo Nordisk Foundation Center for Basic Metabolic Research, Faculty of Health and Medical Sciences, University of Copenhagen, Copenhagen DK-2200, Denmark; 27 Quantitative Sciences, PCPS, GlaxoSmithKline, North Carolina 27709, USA; 28 Institute of Clinical Medicine, Internal Medicine, University of Eastern Finland, Kuopio FI-70211, Finland; 29 Department of Medicine and Department of Biomedical Sciences, Cedars-Sinai Medical Center, Los Angeles, California 90048, USA; 30 Materials and Process Simulation Center, California Institute of Technology, Pasadena, California 91125, USA; 31 CEA, Institut de Génomique, Centre National de Génotypage, 2 Rue Gaston Crémieux, EVRY Cedex 91057, France; 32 Brown Foundation Institute of Molecular Medicine, University of Texas Health Science Center, Houston, Texas 77030, USA; 33 The Wellcome Trust Sanger Institute, Hinxton CB10 1SA, UK; 34 Department of Public Health and Primary Care, Strangeways Research Laboratory, University of Cambridge, Cambridge CB1 8RN, UK; 35 Intramural Research Program, National Institute on Aging, Bethesda, Maryland 21224, USA; 36 Institute for Translational Genomics and Population Sciences, Los Angeles Biomedical Research Institute at Harbor-UCLA Medical Center, Torrance, California 90502, USA; 37 Icelandic Heart Association, Holtasmari 1, Kopavogur IS-201, Iceland; 38 Department of Genetics, University of North Carolina, Chapel Hill, North Carolina 27599, USA; 39 Indiana University, Fairbanks School of Public Health, Indianapolis, Indiana 46202, USA; 40 Department of Epidemiology, Johns Hopkins University, Baltimore, Maryland 21205, USA; 41 Department of Molecular Epidemiology, German Institute of Human Nutrition Potsdam-Rehbrücke, Nuthetal DE-14558, Germany; 42 Human Genetics Center, School of Public Health, The University of Texas Health Science Center at Houston, Houston, Texas 77225, USA; 43 Laboratory of Neurogenetics, National Institute on Aging, Bethesda, Maryland 20892, USA; 44 Department of Internal Medicine, Erasmus University Medical Center, Rotterdam 3000 CE, The Netherlands; 45 The Netherlands Genomics Initiative-sponsored Netherlands Consortium for Healthy Aging (NGI-NCHA), Leiden/Rotterdam 2300 RC, The Netherlands; 46 Atherosclerosis Research Unit, Department of Medicine Solna, Karolinska Institutet, Stockholm SE-171 77, Sweden; 47 University of Iceland, Reykjavik IS-101, Iceland; 48 Wellcome Trust Centre for Human Genetics, Oxford OX3 7BN, UK; 49 Department of Genome Dynamics, Lawrence Berkeley National Laboratory, Berkeley, California 94720, USA; 50 Department of Clinical Sciences, Genetic and Molecular Epidemiology Unit, Lund University, Skåne University Hospital, Malmö SE-205 02, Sweden; 51 Singapore Eye Research Institute, Singapore National Eye Centre, Singapore 168751, Singapore; 52 Department of Ophthalmology, National University of Singapore and National University Health System, Singapore 119228, Singapore; 53 Division of Genetics and Cell Biology, San Raffaele Research Institute, Milano 20132, Italy; 54 Predoctoral Training Program in Human Genetics, McKusick-Nathans Institute of Genetic Medicine, Johns Hopkins University School of Medicine, Maryland 21205, USA; 55 McKusick-Nathans Institute of Genetic Medicine, Johns Hopkins University School of Medicine, Baltimore, Maryland 21205, USA; 56 Department of Biostatistics and Center for Statistical Genetics, University of Michigan, Ann Arbor, Michigan 48109, USA; 57 Section of Biology and Genetics, Department of Life and Reproduction Sciences, University of Verona, Verona 37100, Italy; 58 Department of Biochemistry, Wake Forest School of Medicine, Winston-Salem, North Carolina 27157, USA; 59 Saw Swee Hock School of Public Health, National University of Singapore and National University Health System, Singapore 119228, Singapore; 60 Office of Clinical Sciences, Duke-NUS Graduate Medical School, National University of Singapore, Singapore 169857, Singapore; 61 Department of Medicine, University of Mississippi Medical Center, Jackson, Mississippi 39216, USA; 62 Department of Epidemiology, Erasmus University Medical Center, Rotterdam 3000 CE, The Netherlands; 63 McKusick-Nathans Institute of Genetic Medicine, Johns Hopkins University, Baltimore, Maryland 21205, USA; 64 Division of Cardiology, Geneva University Hospital Geneva 1211, Switzerland; 65 Department of Nutrition and Dietetics, School of Health Science and Education, Harokopio University, Athens 17671, Greece; 66 Department of Numerical Analysis and Computer Science, SciLifeLab, Stockholm University, Stockholm SE-106 91, Sweden; 67 Division of Nephrology, Department of Internal Medicine and Medical Specialties, Columbus-Gemelli University Hospital, Catholic University, Rome 00168, Italy; 68 Department of Cardiovascular Medicine, The Wellcome Trust Centre for Human Genetics, University of Oxford, Oxford OX3 7BN, UK; 69 Department of Medical Sciences, Molecular Epidemiology and Science for Life Laboratory, Uppsala University, Uppsala SE-751 85, Sweden; 70 Department of Biobank Research, Umeå University, Umeå SE-901 87, Sweden; 71 Department of Biomedical Technology, Sangmyung University, Chungnam 330-720, Korea; 72 Institute of Human Genetics, Department of Genomics, Life & Brain Center, University of Bonn, Bonn DE-53127, Germany; 73 Human Genomics Research Group, Division of Medical Genetics, University Hospital Basel Department of Biomedicine 4031, Basel, Switzerland; 74 Institute of Neuroscience and Medicine (INM-1) Genomic Imaging Research Center Juelich, Juelich DE-52425, Germany; 75 Memory Aging & Cognition Centre (MACC), National University Health System, Singapore 117599, Singapore; 76 Steno Diabetes Center, Gentofte DK-2820, Denmark; 77 Research Centre for Prevention and Health, Glostrup University Hospital, Glostrup DK-2600, Denmark; 78 Faculty of Medicine, University of Aalborg, Aalborg DK-9220, Denmark; 79 Echinos Medical Centre, Echinos 67300, Greece; 80 Division of Human Genetics, Genome Institute of Singapore, Singapore 138672, Singapore; 81 Institute of Clinical Medicine, Internal Medicine, University of Eastern Finland and Kuopio University Hospital, Kuopio FI-70211, Finland; 82 Department of Biostatistics, University of North Carolina, Chapel Hill, North Carolina 27599, USA; 83 Division of Endocrine and Metabolism, Department of Internal Medicine, Taichung Veterans General Hospital, Taichung 407, Taiwan; 84 School of Medicine, National Yang-Ming University, Taipei 112, Taiwan; 85 Department of Medical Research, Taichung Veterans General Hospital, Taichung 407, Taiwan; 86 Department of Epidemiology & Prevention, Division of Public Health Sciences, Wake Forest University, Winston-Salem, North Carolina 27106, USA; 87 Wellcome Trust Centre for Human Genetics, University of Oxford, Oxford OX3 7BN, UK; 88 Department of Clinical Experimental Research, Copenhagen University Hospital Glostrup, Glostrup DK-2600, Denmark; 89 Department of Clinical Medicine, Faculty of Health and Medical Sciences, University of Copenhagen, Copenhagen DK-2200, Denmark; 90 National and Kapodistrian University of Athens, Faculty of Medicine, Athens 115 27, Greece; 91 Dromokaiteio Psychiatric Hospital, Athens 124 61, Greece; 92 Division of General Internal Medicine, Johns Hopkins University School of Medicine, Baltimore, Maryland 21205, USA; 93 Welch Center for Prevention, Epidemiology, and Clinical Research, Johns Hopkins University, Baltimore, Maryland 21205, USA; 94 Department of Preventive Medicine, Keck School of Medicine of the University of Southern California, Los Angeles 90033, USA; 95 Human Genome Sequencing Center, Baylor College of Medicine, Houston, Texas 77030, USA; 96 University of Leicester, Leicester LE1 7RH, UK; 97 Center for Genomics and Personalized Medicine Research, Wake Forest School of Medicine, Winston-Salem, North Carolina 27106, USA; 98 Department of Internal Medicine, Division of Endocrinology, Metabolism, Pathobiochemistry and Clinical Chemistry and Institute of Diabetes Research and Metabolic Diseases, University of Tübingen, Tübingen DE-72076, Germany; 99 German Center for Diabetes Research (DZD), Neuherberg DE-85764, Germany; 100 The Oxford Centre for Diabetes, Endocrinology and Metabolism, University of Oxford, Oxford OX3 7LE, UK; 101 Department of Biostatistics, University of Washington, Seattle, Washington 98195, USA; 102 Science for Life Laboratory, Karolinska Institutet, Stockholm SE-171 77, Sweden; 103 Department of Gastroenterology, Gennimatas General Hospital, Athens 11527, Greece; 104 Department of Epidemiology, School of Public Health, University of Michigan, Ann Arbor, Michigan 48109, USA; 105 Department of Hematology, Long Road, Cambridge CB2 0XY, UK; 106 Department of Internal Medicine, Division of Gastroenterology and Department of Computational Medicine and Bioinformatics, University of Michigan, Ann Arbor, Michigan 48109, USA; 107 Division of Psychiatric Genomics, The Icahn School of Medicine at Mount Sinai, New York, New York 10029, USA; 108 William Harvey Research Institute, Barts and The London School of Medicine and Dentistry, Queen Mary University of London, London E1 4NS, UK; 109 First Department of Propaedeutic and Internal Medicine, Athens University Medical School, Laiko General Hospital, Athens 11527, Greece; 110 Diabetes Centre, 2nd Department of Internal Medicine, National University of Athens, Hippokration General Hospital, Athens 11527, Greece; 111 Anogia Medical Centre, Anogia 740 51, Greece; 112 The GeneSTAR Research Program, Division of General Internal Medicine, Department of Medicine, The Johns Hopkins University School of Medicine, Baltimore, Maryland 21205, USA; 113 University of Sheffield, Sheffield S10 2TN, UK; 114 Department of Statistics, University of California at Berkeley, Berkeley, California 94720, USA; 115 Faculty of Health Science, University of Copenhagen, Copenhagen 1165, Denmark; 116 Division of Research, Kaiser Permanente, Northern California Region, Oakland, California 94612, USA; 117 Department of Medicine, University of Texas Health Science Center, San Antonio, Texas 77030, USA; 118 Department of Epidemiology, Colorado School of Public Health, University of Colorado Denver, Aurora, Colorado 80204, USA; 119 Program in Medical and Population Genetics, Broad Institute, Cambridge, Massachusetts 02142, USA; 120 Center for Human Genetic Research, Massachusetts General Hospital, Boston, Massachusetts 02114, USA; 121 College of Medicine, National Defense Medical Center, Taipei 114, Taiwan; 122 Division of Public Health Sciences, Wake Forest School of Medicine, Winston-Salem, North Carolina 27106, USA; 123 Department of Epidemiology, German Institute of Human Nutrition Potsdam Rehbrücke, Nuthetal DE-14558, Germany; 124 Wellcome Trust Sanger Institute, Hinxton, Cambridge CB10 1SA, UK; 125 Princess Al-Jawhara Al-Brahim Centre of Excellence in Research of Hereditary Disorders (PACER-HD), King Abdulaziz University, Jeddah 22254, Saudi Arabia; 126 CNR Institute of Clinical Physiology, Pisa 73100, Italy; 127 Department of Nutrition, Harvard School of Public Health, Boston, Massachusetts 02115, USA; 128 Department of Public Health & Clinical Medicine, Umeå University, Umeå SE-901 87, Sweden; 129 Genetics of Diabetes, University of Exeter Medical School, University of Exeter, Exeter EX1 2LU, UK; 130 Research Unit, Skellefteå SE-931 87, Sweden; 131 Population Sciences Branch, National Heart, Lung, and Blood Institute, National Institutes of Health, Bethesda, Maryland 20892, USA; 132 Framingham Heart Study, Framingham, Massachusetts 01702, USA; 133 Cardiology Division, Department of Medicine, Massachusetts General Hospital and Harvard Medical School, Boston, Massachusetts 02115, USA; 134 University of Cambridge Metabolic Research Laboratories, MRC Metabolic Diseases Unit and NIHR Cambridge Biomedical Research Centre, Wellcome Trust-MRC Institute of Metabolic Science, Addenbrooke’s Hospital, Cambridge CB2 1TN, UK; 135 Institute of Cardiovascular and Medical Sciences, University of Glasgow, Glasgow G12 8TA, UK; 136 Division of Epidemiology and Community Health, School of Public Health, University of Minnesota, Minneapolis, Minnesota 55455, USA; 137 Department of Public Health, Faculty of Medicine, University of Split, Split 21000, Croatia; 138 Center for Public Health Genomics, Department of Public Health Sciences, University of Virginia, Charlottesville, Virginia 22908, USA; 139 Division of Cardiology, Brigham and Women’s Hospital and Harvard Medical School, Boston, Massachusetts 02115, USA; 140 Centre for Population Health Sciences, Medical School, University of Edinburgh, Edinburgh, Scotland EH8 9YL, UK; 141 Medical Research Institute, University of Dundee, Dundee DD1 9SY, UK; 142 Institute of Cellular Medicine, Newcastle University, Newcastle-upon-Tyne NE1 7RU, UK; 143 Division of Genetics, Brigham and Women's Hospital and Harvard Medical School, Boston, Massachusetts, USA; 144 Department of Epidemiology, University of Washington, Seattle, Washington 98195, USA; 145 Department of Health Services, University of Washington, Seattle, Washington 98195, USA; 146 Group Health Research Institute, Group Health Cooperative, Seattle, Washington 98195, USA; 147 Department of Medicine, Yong Loo Lin School of Medicine, National University of Singapore, Singapore 117597, Singapore; 148 Center for Medical Systems Biology, Leiden 2300, The Netherlands; 149 Genetics, PCPS, GlaxoSmithKline, Philadelphia, Pennsylvania 19104, USA; 150 Department of Medicine, Johns Hopkins University, Baltimore, Maryland 21205, USA; 151 The Mindich Child Health and Development Institute, The Icahn School of Medicine at Mount Sinai, New York, New York 10029, USA; 152 Department of Physiology and Biophysics, University of Mississippi Medical Center, Jackson, Mississippi 38677, USA; 153 New York Academy of Medicine, New York, New York 10029, USA; 154 Cardiovascular Health Research Unit, Departments of Medicine and Epidemiology, University of Washington, Seattle, Washington 98195, USA; 155 Division of Endocrinology, Diabetes and Metabolism, Cedars-Sinai Medical Center, Los Angeles, California 90048, USA; 156 Oxford NIHR Biomedical Research Centre, Oxford, UK; 157 Public Health Division of Gipuzkoa, San Sebastian, Spain; 158 Instituto BIO–Donostia, Basque Government, San Sebastian, Spain; 159 CIBER Epidemiología y Salud Pública (CIBERESP), Spain; 160 Inserm, CESP, U1018, Villejuif, France; 161 Univ Paris–Sud, UMRS 1018, Villejuif, France; 162 Navarre Public Health Institute (ISPN), Pamplona, Spain; 163 Catalan Institute of Oncology (ICO), Barcelona, Spain; 164 Epidemiology and Prevention Unit, Milan, Italy; 165 German Cancer Research Centre (DKFZ), Heidelberg, Germany; 166 Cancer Epidemiology Unit, Nuffield Department of Population Health, University of Oxford, Oxford, UK; 167 Department of Epidemiology, Murcia Regional Health Council, Murcia, Spain; 168 Unit of Preventive Medicine and Public Health, School of Medicine, University of Murcia, Murcia, Spain; 169 Department of Public Health, Section for Epidemiology, Aarhus University, Aarhus, Denmark; 170 Aalborg University Hospital, Aalborg, Denmark; 171 Cancer Research and Prevention Institute (ISPO), Florence, Italy; 172 Dipartimento di Medicina Clinica e Chirurgia, Federico II University, Naples, Italy; 173 Public Health Directorate, Asturias, Spain; 174 Unit of Cancer Epidemiology, Citta' della Salute e della Scienza Hospital–University of Turin and Center for Cancer Prevention (CPO), Torino, Italy; 175 Human Genetics Foundation (HuGeF), Torino, Italy; 176 Andalusian School of Public Health, Granada, Spain; 177 Instituto de Investigación Biosanitaria de Granada (Granada.ibs), Granada, Spain; 178 International Agency for Research on Cancer, Lyon, France; 179 Danish Cancer Society Research Center, Copenhagen, Denmark; 180 ASP Ragusa, Italy; 181 Aire Onlus, Ragusa, Italy; 182 National Institute for Public Health and the Environment (RIVM), Bilthoven, The Netherlands; 183 University Medical Center Utrecht, Utrecht, Utrecht, the Netherlands; 184 School of Public Health, Imperial College London, London, UK

## Abstract

Fasting glucose and insulin are intermediate traits for type 2 diabetes. Here we explore the role of coding variation on these traits by analysis of variants on the HumanExome BeadChip in 60,564 non-diabetic individuals and in 16,491 T2D cases and 81,877 controls. We identify a novel association of a low-frequency nonsynonymous SNV in *GLP1R* (A316T; rs10305492; MAF=1.4%) with lower FG (*β*=−0.09±0.01 mmol l^−1^, *P*=3.4 × 10^−12^), T2D risk (OR[95%CI]=0.86[0.76–0.96], *P*=0.010), early insulin secretion *(β*=−0.07±0.035 pmol_insulin_ mmol_glucose_^−1^, *P*=0.048), but higher 2-h glucose *(β*=0.16±0.05 mmol l^−1^, *P*=4.3 × 10^−4^). We identify a gene-based association with FG at *G6PC2* (*p*_SKAT_=6.8 × 10^−6^) driven by four rare protein-coding SNVs (H177Y, Y207S, R283X and S324P). We identify rs651007 (MAF=20%) in the first intron of *ABO* at the putative promoter of an antisense lncRNA, associating with higher FG *(β*=0.02±0.004 mmol l^−1^, *P*=1.3 × 10^−8^). Our approach identifies novel coding variant associations and extends the allelic spectrum of variation underlying diabetes-related quantitative traits and T2D susceptibility.

## Abstract (web summary)

Both rare and common variants contribute to the aetiology of complex traits such as type 2 diabetes (T2D). Here, the authors examine the effect of coding variation on glycaemic traits and T2D, and identify low-frequency variation in *GLP1R* significantly associated with these traits.

## Graphical abstract

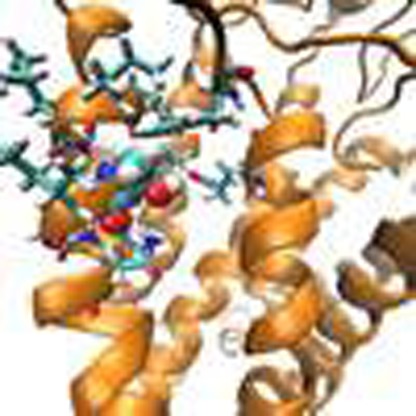

Genome-wide association studies (GWAS) highlight the role of common genetic variation in quantitative glycaemic traits and susceptibility to type 2 diabetes (T2D)^1^,^2^. However, recent large-scale sequencing studies report that rapid expansions in the human population have introduced a substantial number of rare genetic variants^3^,^4^, with purifying selection having had little time to act, which may harbour larger effects on complex traits than those observed for common variants^3^,^5^,^6^. Recent efforts have identified the role of low frequency and rare coding variation in complex disease and related traits^7^,^8^,^9^,^10^, and highlight the need for large sample sizes to robustly identify such associations^11^. Thus, the Illumina HumanExome BeadChip (or exome chip) has been designed to allow the capture of rare (MAF<1%), low frequency (MAF=1–5%) and common (MAF≥5%) exonic single nucleotide variants (SNVs) in large sample sizes.

To identify novel coding SNVs and genes influencing quantitative glycaemic traits and T2D, we perform meta-analyses of studies participating in the Cohorts for Heart and Aging Research in Genomic Epidemiology (CHARGE^12^) T2D-Glycemia Exome Consortium^13^. Our results show a novel association of a low frequency coding variant in *GLP1R*, a gene encoding a drug target in T2D therapy (the incretin mimetics), with FG and T2D. The minor allele is associated with lower FG, lower T2D risk, lower insulin response to a glucose challenge and higher 2-h glucose, pointing to physiological effects on the incretin system. Analyses of non-synonymous variants also enable us to identify particular genes likely to underlie previously identified associations at six loci associated with FG and/or FI (*G6PC2, GPSM1, SLC2A2*, *SLC30A8, RREB1* and *COBLL1)* and five with T2D (*ARAP1*, *GIPR*, *KCNJ11, SLC30A8* and *WFS1*). Further, we found non-coding variants whose putative functions in epigenetic and post-transcriptional regulation of *ABO* and *G6PC2* are supported by experimental ENCODE Consortium, GTEx and transcriptome data from islets. In conclusion, our approach identifies novel coding and non-coding variants and extends the allelic and functional spectrum of genetic variation underlying diabetes-related quantitative traits and T2D susceptibility.

## Results

An overview of the study design is shown in Supplementary Fig. 1, and participating studies and their characteristics are detailed in Supplementary Data 1. We conducted single variant and gene-based analyses for fasting glucose (FG) and fasting insulin (FI), by combining data from 23 studies comprising up to 60,564 (FG) and 48,118 (FI) non-diabetic individuals of European and African ancestry. We followed up associated variants at novel and known glycaemic loci by tests of association with T2D, additional physiological quantitative traits (including post-absorptive glucose and insulin dynamic measures), pathway analyses, protein conformation modelling, comparison with whole-exome sequence data and interrogation of functional annotation resources including ENCODE^14^,^15^ and GTEx^16^. We performed single-variant analyses using additive genetic models of 150,558 SNVs (*P* value for significance ≤3 × 10^−7^) restricted to MAF>0.02% (equivalent to a minor allele count (MAC) ≥20), and gene-based tests using Sequence Kernel Association (SKAT) and Weighted Sum Tests (WST) restricted to variants with MAF<1% in a total of 15,260 genes (*P* value for significance ≤2 × 10^−6^, based on number of gene tests performed). T2D case/control analyses included 16,491 individuals with T2D and 81,877 controls from 22 studies (Supplementary Data 2).

### Novel association of a GLP1R variant with glycaemic traits

**Figure 1:**
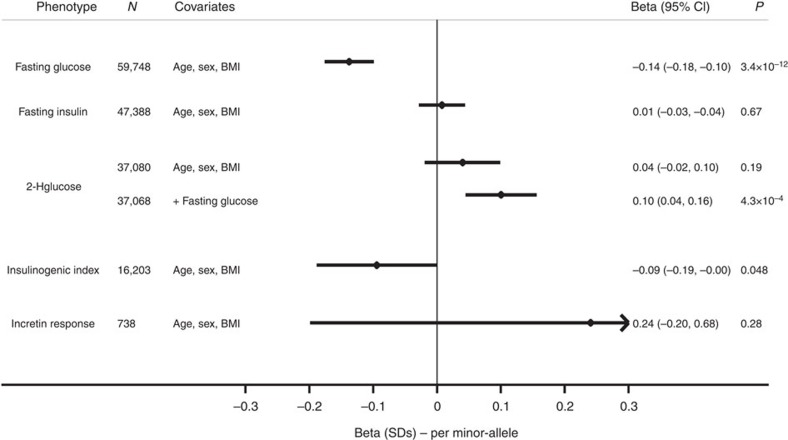
Glycaemic associations with rs10305492 (*GLP1R* A316T). Glycaemic phenotypes were tested for association with rs10305492 in *GLP1R* (A316T). Each phenotype, sample size (*N*), covariates in each model, beta per s.d., 95% confidence interval (95%CI) and *P* values (*P*) are reported. Analyses were performed on native distributions and scaled to s.d. values from the Fenland or Ely studies to allow comparisons of effect sizes across phenotypes.

We identified a novel association of a nonsynonymous SNV (nsSNV) (A316T, rs10305492, MAF=1.4%) in the gene encoding the receptor for glucagon-like peptide 1 (*GLP1R)*, with the minor (A) allele associated with lower FG (*β*=−0.09±0.01 mmol l^−1^ (equivalent to 0.14 SDs in FG), *P*=3.4 × 10^−12^, variance explained=0.03%, Table 1 and Fig. 1), but not with FI (*P*=0.67, Supplementary Table 1). GLP-1 is secreted by intestinal L-cells in response to oral feeding and accounts for a major proportion of the so-called ‘incretin effect’, that is, the augmentation of insulin secretion following an oral glucose challenge relative to an intravenous glucose challenge. GLP-1 has a range of downstream actions including glucose-dependent stimulation of insulin release, inhibition of glucagon secretion from the islet alpha-cells, appetite suppression and slowing of gastrointestinal motility^17^,^18^. In follow-up analyses, the FG-lowering minor A allele was associated with lower T2D risk (OR [95%CI]=0.86 [0.76–0.96], *P*=0.010, Supplementary Data 3). Given the role of incretin hormones in post-prandial glucose regulation, we further investigated the association of A316T with measures of post-challenge glycaemia, including 2-h glucose, and 30 min-insulin and glucose responses expressed as the insulinogenic index^19^ in up to 37,080 individuals from 10 studies (Supplementary Table 2). The FG-lowering allele was associated with higher 2-h glucose levels (β in SDs per-minor allele [95%CI]: 0.10 [0.04, 0.16], *P*=4.3 × 10^−4^, N=37,068) and lower insulinogenic index (−0.09 [−0.19, −0.00], *P*=0.048, *N*=16,203), indicating lower early insulin secretion (Fig. 1). Given the smaller sample size, these associations are less statistically compelling; however, the directions of effect indicated by their beta values are comparable to those observed for fasting glucose. We did not find a significant association between A316T and the measure of ‘incretin effect’, but this was only available in a small sample size of 738 non-diabetic individuals with both oral and intravenous glucose tolerance test data (β in SDs per-minor allele [95%CI]: 0.24 [−0.20–0.68], *P*=0.28, Fig. 1 and Supplementary Table 2). We did not see any association with insulin sensitivity estimated by euglycaemic-hyperinsulinemic clamp or frequently sampled IV glucose tolerance test (Supplementary Table 3). While stimulation of the GLP-1 receptor has been suggested to reduce appetite^20^ and treatment with GLP1R agonists can result in reductions in BMI^21^, these potential effects are unlikely to influence our results, which were adjusted for BMI.

In an effort to examine the potential functional consequence of the *GLP1R* A316T variant, we modelled the A316T receptor mutant structure based on the recently published^22^ structural model of the full-length human GLP-1 receptor bound to exendin-4 (an exogenous GLP-1 agonist). The mutant structural model was then relaxed in the membrane environment using molecular dynamics simulations. We found that the T316 variant (in transmembrane (TM) domain 5) disrupts hydrogen bonding between N320 (in TM5) and E364 (TM6) (Supplementary Fig. 2). In the mutant receptor, T316 displaces N320 and engages in a stable interaction with E364, resulting in slight shifts of TM5 towards the cytoplasm and TM6 away from the cytoplasm (Supplementary Figs 3 and 4). This alters the conformation of the third intracellular loop, which connects TM5 and TM6 within the cell, potentially affecting downstream signalling through altered interaction with effectors such as G proteins.

**Figure 2:**
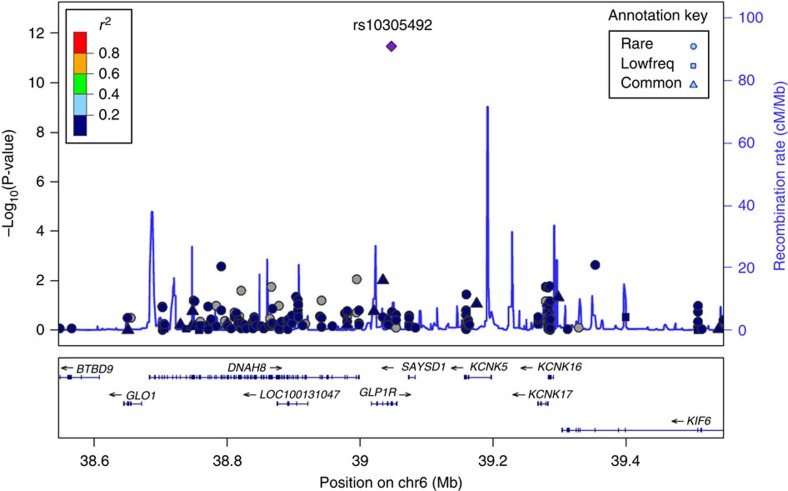
*GLP1R* regional association plot. Regional association results (−log_10_*p*) for fasting glucose of *GLP1R* locus on chromosome 6. Linkage disequilibrium (*r*^2^) indicated by colour scale legend. Triangle symbols indicate variants with MAF>5%, square symbols indicate variants with MAF1–5% and circle symbols indicate variants with MAF<1%.

A targeted Gene Set Enrichment Analysis (Supplementary Table 4) identified enrichment of genes biologically related to *GLP1R* in the incretin signalling pathway (*P*=2 × 10^−4^); after excluding *GLP1R* and previously known loci *PDX1, GIPR* and *ADCY5*, the association was attenuated (*P*=0.072). Gene-based tests at *GLP1R* did not identify significant associations with glycaemic traits or T2D susceptibility, further supported by Fig. 2, which indicates only one variant in the *GLP1R* region on the exome chip showing association with FG.

To more fully characterize the extent of local sequence variation and its association with FG at *GLP1R*, we investigated 150 *GLP1R* SNVs identified from whole-exome sequencing in up to 14,118 individuals available in CHARGE and the GlaxoSmithKline discovery sequence project (Supplementary Table 5). Single-variant analysis identified association of 12 other SNVs with FG (*P*<0.05; Supplementary Data 4), suggesting that additional variants at this locus may influence FG, including two variants (rs10305457 and rs761386) in close proximity to splice sites that raise the possibility that their functional impact is exerted via effects on *GLP1R* pre-mRNA splicing. However, the smaller sample size of the sequence data limits power for firm conclusions.

### Association of noncoding variants in ABO with glycaemic traits

We also newly identified that the minor allele A at rs651007 near the *ABO* gene was associated with higher FG (*β*=0.02±0.004 mmol l^−1^, MAF=20%, *P*=1.3 × 10^−8^, variance explained=0.02%, Table 1). Three other associated common variants in strong linkage disequilibrium (LD) (r^2^=0.95–1) were also located in this region; conditional analyses suggested that these four variants reflect one association signal (Supplementary Table 6). The FG-raising allele of rs651007 was nominally associated with increased FI (*β*=0.008±0.003, *P*=0.02, Supplementary Table 1) and T2D risk (OR [95%CI]=1.05 [1.01–1.08], *P*=0.01, Supplementary Data 3). Further, we independently replicated the association at this locus with FG in non-overlapping data from MAGIC^1^ using rs579459, a variant in LD with rs651007 and genotyped on the Illumina CardioMetabochip (*β*=0.008±0.003 mmol l^−1^, *P*=5.0 × 10^−3^; *N*_MAGIC_=88,287). The FG-associated SNV at *ABO* was in low LD with the three variants^23^ that distinguish between the four major blood groups O, A_1_, A_2_ and B (rs8176719 *r*^2^=0.18, rs8176749 *r*^2^=0.01 and rs8176750 *r*^2^=0.01). The blood group variants (or their proxies) were not associated with FG levels (Supplementary Table 7).

Variants in the *ABO* region have been associated with a number of cardiovascular and metabolic traits in other studies (Supplementary Table 8), suggesting a broad role for this locus in cardiometabolic risk. A search of the four FG-associated variants and their associations with metabolic traits using data available through other CHARGE working groups (Supplementary Table 9) revealed a significant association of rs651007 with BMI in women (*β*=0.025±0.01 kg m^−2^, *P*=3.4 × 10^−4^) but not in men. As previously reported^24^,^25^, the FG increasing allele of rs651007 was associated with increased LDL and TC (LDL: *β*=2.3±0.28 mg dl^−1^, *P*=6.1 × 10^−16^; TC: *β*=2.4±0.33 mg dl^−1^, *P*=3.4 × 10^−13^). As the FG-associated *ABO* variants were located in non-coding regions (intron 1 or intergenic) we interrogated public regulatory annotation data sets, GTEx^16^ (http://www.gtexportal.org/home/) and the ENCODE Consortium resources^14^ in the UCSC Genome Browser^15^ (http://genome.ucsc.edu/) and identified a number of genomic features coincident with each of the four FG-associated variants. Three of these SNPs, upstream of the ABO promoter, reside in a DNase I hypersensitive site with canonical enhancer marks in ENCODE Consortium data: H3K4Me1 and H3K27Ac (Supplementary Fig. 5). We analysed all SNPs with similar annotations, and found that these three are coincident with DNase, H3K4Me1 and H3K27Ac values each near the genome-wide mode of these assays (Supplementary Fig. 6). Indeed, in haematopoietic model K562 cells, the ENCODE Consortium has identified the region overlapping these SNPs as a putative enhancer^14^. Interrogating the GTEx database (*N*=156), we found that rs651007 (*P*=5.9 × 10^−5^) and rs579459 (*P*=6.7 × 10^−5^) are eQTLs for *ABO*, and rs635634 (*P*=1.1 × 10^−4^) is an eQTL for *SLC2A6* in whole blood (Supplementary Table 10). The fourth SNP, rs507666, resides near the transcription start site of a long non-coding RNA that is antisense to exon 1 of *ABO* and expressed in pancreatic islets (Supplementary Fig. 5). rs507666 was also an eQTL for the glucose transporter *SLC2A6* (*P*=1.1 × 10^−4^) (Supplementary Fig. 5 and Supplementary Table 10). *SLC2A6* codes for a glucose transporter whose relevance to glycaemia and T2D is largely unknown, but expression is increased in rodent models of diabetes^26^. Gene-based analyses did not reveal significant quantitative trait associations with rare coding variation in *ABO*.

### Rare variants in G6PC2 are associated with fasting glucose

**Figure 3:**
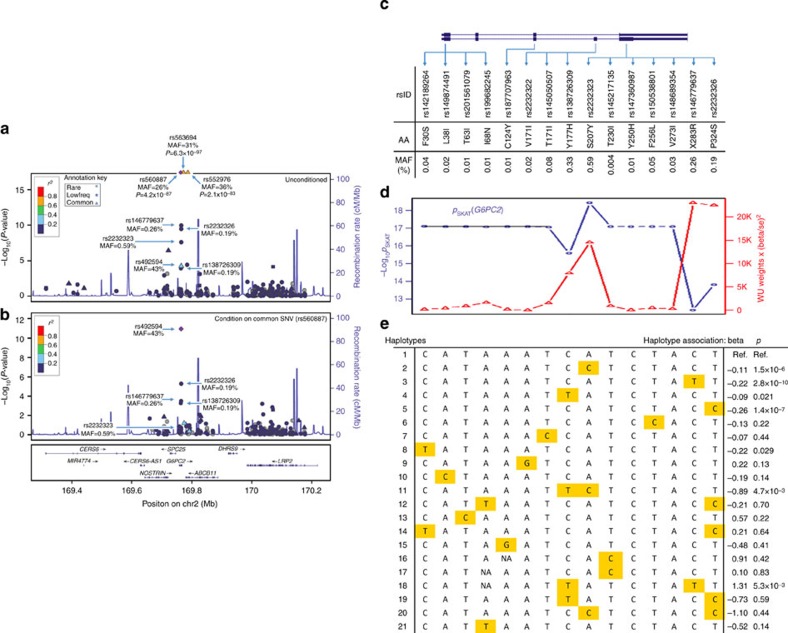
G6PC2. (**a**) Regional association results (−log_10_*p*) for fasting glucose of the *G6PC2* locus on chromosome 2. Minor allele frequencies (MAF) of common and rare *G6PC2* SNVs from single-variant analyses are shown. *P* values for rs560887, rs563694 and rs552976 were artificially trimmed for the figure. Linkage disequilibrium (*r*^2^) indicated by colour scale legend. *y*-Axis scaled to show associations for variant rs560887 (purple dot, MAF=43%, *P*=4.2 × 10^−87^). Triangle symbols indicate variants with MAF>5%, square symbols indicate variants with MAF1–5% and circle symbols indicate variants with MAF <1%. (**b**) Regional association results (−log_10_*p*) for fasting glucose conditioned on rs560887 of *G6PC2*. After adjustment for rs560887, both rare SNVs rs2232326 (S324P) and rs146779637 (R283X), and common SNV rs492594 remain significantly associated with FG indicating the presence of multiple independent associations with FG at the *G6PC2* locus. (**c**) Inset of *G6PC2* gene with depiction of exon locations, amino-acid substitutions and MAFs of the 15 SNVs included in gene-based analysis (MAF<1% and nonsynonymous, splice-site and gain/loss-of-function variation types as annotated by dbNSFPv2.0). (**d**) The contribution of each variant on significance and effect of the SKAT test when one variant is removed from the test. Gene-based SKAT *P* values (blue line) and test statistic (red line) of *G6PC2* after removing one SNV at a time and re-calculating the association. (**e**) Haplotypes and haplotype association statistics and *P* values generated from the 15 rare SNVs from gene-based analysis of *G6PC2* from 18 cohorts and listed in panel (**c**). Global haplotype association, *P*=1.1 × 10^−17^. Haplotypes ordered by decreasing frequency with haplotype 1 as the reference. Orange highlighting indicates the minor allele of the SNV on the haplotype.

At the known glycaemic locus *G6PC2*, gene-based analyses of 15 rare predicted protein-altering variants (MAF<1%) present on the exome chip revealed a significant association of this gene with FG (cumulative MAF of 1.6%, *p*_SKAT_=8.2 × 10^−18^, *p*_WST_=4.1 × 10^−9^; Table 2). The combination of 15 rare SNVs remained associated with FG after conditioning on two known common SNVs in LD^27^ with each other (rs560887 in intron 1 of *G6PC2* and rs563694 located in the intergenic region between *G6PC2* and *ABCB11*) (conditional *p*_SKAT_=5.2 × 10^−9^, *p*_WST_=3.1 × 10^−5^; Table 2 and Fig. 3), suggesting that the observed rare variant associations were distinct from known common variant signals. Although *ABCB11* has been proposed to be the causal gene at this locus^28^, identification of rare and putatively functional variants implicates *G6PC2* as the much more likely causal candidate. As rare alleles that increase risk for common disease may be obscured by rare, neutral mutations^4^, we tested the contribution of each *G6PC2* variant by removing one SNV at a time and re-calculating the evidence for association across the gene. Four SNVs, rs138726309 (H177Y), rs2232323 (Y207S), rs146779637 (R283X) and rs2232326 (S324P), each contributed to the association with FG (Fig. 3c and Supplementary Table 11). Each of these SNVs also showed association with FG of larger effect size in unconditional single-variant analyses (Supplementary Data 5), consistent with a recent report in which H177Y was associated with lower FG levels in Finnish cohorts^29^. We developed a novel haplotype meta-analysis method to examine the opposing direction of effects of each SNV. Meta-analysis of haplotypes with the 15 rare SNVs showed a significant global test of association with FG (*p*_global test_=1.1 × 10^−17^) (Supplementary Table 12) and supported the findings from the gene-based tests. Individual haplotype tests showed that the most significantly associated haplotypes were those carrying a single rare allele at R283X (*P*=2.8 × 10^−10^), S324P (*P*=1.4 × 10^−7^) or Y207S (*P*=1.5 × 10^−6^) compared with the most common haplotype. Addition of the known common intronic variant (rs560887) resulted in a stronger global haplotype association test (*p*_global test_=1.5 × 10^−81^), with the most strongly associated haplotype carrying the minor allele at rs560887 (Supplementary Table 13). Evaluation of regulatory annotation found that this intronic SNV is near the splice acceptor of intron 3 (RefSeq: NM_021176.2) and has been implicated in *G6PC2* pre-mRNA splicing^30^; it is also near the transcription start site of the expressed sequence tag (EST) DB031634, a potential cryptic minor isoform of *G6PC2* mRNA (Supplementary Fig. 7). No associations were observed in gene-based analysis of *G6PC2* with FI or T2D (Supplementary Tables 14 and 15).

Further characterization of exonic variation in *G6PC2* by exome sequencing in up to 7,452 individuals identified 68 SNVs (Supplementary Table 5), of which 4 were individually associated with FG levels and are on the exome chip (H177Y, MAF=0.3%, *P*=9.6 × 10^−5^; R283X, MAF=0.2%, *P*=8.4 × 10^−3^; S324P, MAF=0.1%, *P*=1.7 × 10^−2^; rs560887, intronic, MAF=40%; *P*=7 × 10^−9^) (Supplementary Data 6). Thirty-six SNVs met criteria for entering into gene-based analyses (each MAF<1%). This combination of 36 coding variants was associated with FG (cumulative MAF=2.7%, *p*_SKAT_=1.4 × 10^−3^, *p*_WST_=5.4 × 10^−4^, Supplementary Table 16). Ten of these SNVs had been included in the exome chip gene-based analyses. Analyses indicated that the 10 variants included on the exome chip data had a stronger association with FG (*p*_SKAT_=1.3 × 10^−3^, *p*_WST_=3.2 × 10^−3^ vs *p*_SKAT_=0.6, *p*_WST_=0.04 using the 10 exome chip or the 26 variants not captured on the chip, respectively, Supplementary Table 16).

### Pathway analyses of FG and FI signals

In agnostic pathway analysis applying MAGENTA (http://www.broadinstitute.org/mpg/magenta/) to all curated biological pathways in KEGG (http://www.genome.jp/kegg/), GO (http://www.geneontology.org), Reactome (http://www.reactome.org), Panther (http://www.pantherdb.org), Biocarta (http://www.biocarta.com) and Ingenuity (http://www.ingenuity.com/) databases, no pathways achieved our Bonferroni-corrected threshold for significance of *P*<1.6 × 10^−6^ for gene set enrichment in either FI or FG data sets (Supplementary Tables 17 and 18). The pathway *P* values were further attenuated when loci known to be associated with either trait were excluded from the analysis. Similarly, even after narrowing the MAGENTA analysis to gene sets in curated databases with names suggestive of roles in glucose, insulin or broader metabolic pathways, we did not identify any pathways that met our Bonferroni-corrected threshold for significance of *P*<2 × 10^−4^ (Supplementary Table 19).

### Testing nonsynonomous variants for association in known loci

Owing to the expected functional effects of protein-altering variants, we tested SNVs (4,513 for FG and 1,281 for FI) annotated as nonsynonymous, splice-site or stop gain/loss by dbNSFP^31^ in genes within 500 kb of known glycaemic variants^1^,^27^,^32^ for association with FG and FI to identify associated coding variants, which may implicate causal genes at these loci (Supplementary Table 20). At the *DNLZ*-*GPSM1* locus, a common nsSNV (rs60980157; S391L) in the *GPSM1* gene was significantly associated with FG (Bonferroni corrected *P* value <1.1 × 10^−5^=0.05/4513 SNVs for FG), and had previously been associated with insulinogenic index^9^. The *GPSM1* variant is common and in LD with the intronic index variant in the *DNLZ* gene (rs3829109) from previous FG GWAS^1^ (*r*^2^_EU_=0.68; 1000 Genomes EU). The association of rs3829109 with FG was previously identified using data from the Illumina CardioMetabochip, which poorly captured exonic variation in the region^1^. Our results implicate *GPSM1* as the most likely causal gene at this locus (Supplementary Fig. 8a). We also observed significant associations with FG for eight other potentially protein-altering variants in five known FG loci, implicating three genes (*SLC30A8*, *SLC2A2* and *RREB1*) as potentially causal, but still undetermined for two loci (*MADD* and *IKBKAP*) (Supplementary Figs 6f–8b). At the *GRB14*/*COBLL1* locus, the known GWAS^1^,^32^ nsSNV rs7607980 in the *COBLL1* gene was significantly associated with FI (Bonferroni corrected *P* value <3.9 × 10^−5^=0.05/1281 SNVs for FI), further suggesting *COBLL1* as the causal gene, despite prior functional evidence that GRB14 may represent the causal gene at the locus^33^ (Supplementary Fig. 8g).

Similarly, we performed analyses for loci previously identified by GWAS of T2D, but only focusing on the 412 protein-altering variants within the exonic coding region of the annotated gene(s) at 72 known T2D loci^2^,^34^ on the exome chip. In combined ancestry analysis, three nsSNVs were associated with T2D (Bonferroni-corrected *P* value threshold (*P*<0.05/412=1.3 × 10^−4^) (Supplementary Data 7). At *WFS1*, *SLC30A8* and *KCNJ11*, the associated exome chip variants were all common and in LD with the index variant from previous T2D GWAS in our population (*r*_EU_^2^: 0.6–1.0; 1000 Genomes), indicating these coding variants might be the functional variants that were tagged by GWAS SNVs. In ancestry stratified analysis, three additional nsSNVs in *SLC30A8*, *ARAP1* and *GIPR* were significantly associated with T2D exclusively in African ancestry cohorts among the same 412 protein-altering variants (Supplementary Data 8), all with MAF>0.5% in the African ancestry cohorts, but MAF<0.02% in the European ancestry cohorts. The three nsSNVs were in incomplete LD with the index variants at each locus (*r*^2^_AF_=0, D’_AF_=1; 1000 Genomes). SNV rs1552224 at *ARAP1* was recently shown to increase *ARAP1* mRNA expression in pancreatic islets^35^, which further supports *ARAP1* as the causal gene underlying the common GWAS signal^36^. The association for nsSNV rs73317647 in *SLC30A8* (OR_AF_[95%CI]: 0.45[0.31–0.65], *p*_AF_=2.4 × 10^−5^, MAF_AF_=0.6%) is consistent with the recent report that rare or low frequency protein-altering variants at this locus are associated with protection against T2D^10^. The protein-coding effects of the identified variants indicate all five genes are excellent causal candidates for T2D risk. We did not observe any other single variant nor gene-based associations with T2D that met chip-wide Bonferroni significance thresholds (*P*<4.5 × 10^−7^ and *P*<1.7 × 10^−6^, respectively).

### Associations at known FG, FI and T2D index variants

For the previous reported GWAS loci, we tested the known FG and FI SNVs on the exome chip. Overall, 34 of the 38 known FG GWAS index SNVs and 17 of the 20 known FI GWAS SNVs (or proxies, *r*^2^≥0.8 1000 Genomes) were present on the exome chip. Twenty-six of the FG and 15 of the FI SNVs met the threshold for significance (*p*_FG_<1.5 × 10^−3^ (0.05/34 FG SNVs), *p*_FI_<2.9 × 10^−3^ (0.05/17 FI SNVs)) and were in the direction consistent with previous GWAS publications. In total, the direction of effect was consistent with previous GWAS publications for 33 of the 34 FG SNVs and for 16 of the 17 FI SNVs (binomial probability: *p*_FG_=2.0 × 10^−9^, *p*_FI_=1.4 × 10^−4^, Supplementary Data 9). Of the known 72 T2D susceptibility loci, we identified 59 index variants (or proxies *r*^2^≥0.8 1000 Genomes) on the exome chip; 57 were in the direction consistent with previous publications (binomial probability: *P*=3.1 × 10^−15^, see Supplementary Data 10). In addition, two of the known MODY variants were on the exome chip. Only *HNF4A* showed nominal significance with FG levels (rs139591750, *P*=3 × 10^−3^, Supplementary Table 21).

## Discussion

Our large-scale exome chip-wide analyses identified a novel association of a low frequency coding variant in *GLP1R* with FG and T2D. The minor allele, which lowered FG and T2D risk, was associated with a lower early insulin response to a glucose challenge and higher 2-h glucose. Although the effect size on fasting glucose is slightly larger than for most loci reported to date, our findings suggest that few low frequency variants have a very large effect on glycaemic traits and further demonstrate the need for large sample sizes to identify associations of low frequency variation with complex traits. However, by directly genotyping low frequency coding variants that are poorly captured through imputation, we were able to identify particular genes likely to underlie previously identified associations. Using this approach, we implicate causal genes at six loci associated with fasting glucose and/or FI (*G6PC2, GPSM1, SLC2A2*, *SLC30A8, RREB1* and *COBLL1)* and five with T2D (*ARAP1*, *GIPR*, *KCNJ11, SLC30A8* and *WFS1*). For example, via gene-based analyses, we identified 15 rare variants in *G6PC2 (p*_SKAT_=8.2 × 10^−18^), which are independent of the common non-coding signals at this locus and implicate this gene as underlying previously identified associations. We also revealed non-coding variants whose putative functions in epigenetic and post-transcriptional regulation of *ABO* and *G6PC2* are supported by experimental ENCODE Consortium, GTEx and transcriptome data from islets and for which future focused investigations using human cell culture and animal models will be needed to clarify their functional influence on glycaemic regulation.

The seemingly paradoxical observation that the minor allele at *GLP1R* is associated with opposite effects on FG and 2-h glucose is not unique to this locus, and is also observed at the *GIPR* locus, which encodes the receptor for gastric inhibitory peptide (GIP), the other major incretin hormone. However, for *GLP1R*, we observe that the FG-lowering allele is associated with lower risk of T2D, while at *GIPR*, the FG-lowering allele is associated with higher risk of T2D (and higher 2-h glucose)^1^. The observation that variation in both major incretin receptors is associated with opposite effects on FG and 2-h glucose is a finding whose functional elucidation will yield new insights into incretin biology. An example where apparently paradoxical findings prompted cellular physiologic experimentation that yielded new knowledge is the *GCKR* variant P446L associated with opposing effects on FG and triglycerides^37^,^38^. The *GCKR* variant was found to increase active cytosolic GCK, promoting glycolysis and hepatic glucose uptake while increasing substrate for lipid synthesis^39^,^40^.

Two studies have characterized the *GLP1R* A316T variant *in vitro*. The first study found no effect of this variant on cAMP response to full-length GLP-1 or exendin-4 (endogenous and exogenous agonists)^41^. The second study corroborated these findings, but documented as much as 75% reduced cell surface expression of T316 compared with wild-type, with no alteration in agonist binding affinity. Although this reduced expression had little impact on agonist-induced cAMP response or ERK1/2 activation, receptors with T316 had greatly reduced intracellular calcium mobilization in response to GLP-1(7-36NH_2_) and exendin-4 (ref. 42). Given that GLP-1 induced calcium mobilization is a key factor in the incretin response, the *in vitro* functional data on T316 are consistent with the reduced early insulin response we observed for this variant, further supported by the Glp1r-knockout mouse, which shows lower early insulin secretion relative to wild-type mice^43^.

The associations of *GLP1R* variation with lower FG and T2D risk are more challenging to explain, and highlight the diverse and complex roles of *GLP1R* in glycaemic regulation. While future experiments will be needed, here we offer the following hypothesis. Given fasting hyperglycaemia observed in Glp1r-knockout mice^43^, A316T may be a gain-of-function allele that activates the receptor in a constitutive manner, causing beta cells to secrete insulin at a lower ambient glucose level, thereby maintaining a lower FG; this could in turn cause downregulation of GLP1 receptors over time, causing incretin resistance and a higher 2-h glucose after an oral carbohydrate load. Other variants in G protein-coupled receptors central to endocrine function such as the TSH receptor (*TSHR*), often in the transmembrane domains^44^ (like A316T, which is in a transmembrane helix (TM5) of the receptor peptide), have been associated with increased constitutive activity alongside reduced cell surface expression^45^,^46^, but blunted or lost ligand-dependent signalling^46^,^47^.

The association of variation in *GLP1R* with FG and T2D represents another instance wherein genetic epidemiology has identified a gene that codes for a direct drug target in T2D therapy (incretin mimetics), other examples including *ABCC8/KCNJ11* (encoding the targets of sulfonylureas) and *PPARG* (encoding the target of thiazolidinediones). In these examples, the drug preceded the genetic discovery. Today, there are over 100 loci showing association with T2D and glycaemic traits. Given that at least three of these loci code for potent antihyperglycaemic targets, these genetic discoveries represent a promising long-term source of potential targets for future diabetes therapies.

In conclusion, our study has shown the use of analysing the variants present on the exome chip, followed-up with exome sequencing, regulatory annotation and additional phenotypic characterization, in revealing novel genetic effects on glycaemic homeostasis and has extended the allelic and functional spectrum of genetic variation underlying diabetes-related quantitative traits and T2D susceptibility.

## Methods

### Study cohorts

The CHARGE consortium was created to facilitate large-scale genomic meta-analyses and replication opportunities among multiple large population-based cohort studies^12^. The CHARGE T2D-Glycemia Exome Consortium was formed by cohorts within the CHARGE consortium as well as collaborating non-CHARGE studies to examine rare and common functional variation contributing to glycaemic traits and T2D susceptibility (Supplementary Note 1). Up to 23 cohorts participated in this effort representing a maximum total sample size of 60,564 (FG) and 48,118 (FI) participants without T2D for quantitative trait analyses. Individuals were of European (84%) and African (16%) ancestry. Full study characteristics are shown in Supplementary Data 1. Of the 23 studies contributing to quantitative trait analysis, 16 also contributed data on T2D status. These studies were combined with six additional cohorts with T2D case–control status for follow-up analyses of the variants observed to influence FG and FI and analysis of known T2D loci in up to 16,491 T2D cases and 81,877 controls across 4 ancestries combined (African, Asian, European and Hispanic; see Supplementary Data 2 for T2D case–control sample sizes by cohort and ancestry). All studies were approved by their local institutional review boards and written informed consent was obtained from all study participants.

### Quantitative traits and phenotypes

FG (mmol l^−1^) and FI (pmol l^−1^) were analysed in individuals free of T2D. FI was log transformed for genetic association tests. Study-specific sample exclusions and detailed descriptions of glycaemic measurements are given in Supplementary Data 1. For consistency with previous glycaemic genetic analyses, T2D was defined by cohort and included one or more of the following criteria: a physician diagnosis of diabetes, on anti-diabetic treatment, fasting plasma glucose ≥7 mmol l^−1^, random plasma glucose ≥11.1 mmol l^−1^ or haemoglobin A1C≥6.5% (Supplementary Data 2).

### Exome chip

The Illumina HumanExome BeadChip is a genotyping array containing 247,870 variants discovered through exome sequencing in ~12,000 individuals, with ~75% of the variants with a MAF<0.5%. The main content of the chip comprises protein-altering variants (nonsynonymous coding, splice-site and stop gain or loss codons) seen at least three times in a study and in at least two studies providing information to the chip design. Additional variants on the chip included common variants found through GWAS, ancestry informative markers (for African and Native Americans), mitochondrial variants, randomly selected synonymous variants, HLA tag variants and Y chromosome variants. In the present study we analysed association of the autosomal variants with glycaemic traits and T2D. See Supplementary Fig. 1 for study design and analysis flow.

### Exome array genotyping and quality control

Genotyping was performed with the Illumina HumanExome BeadChipv1.0 (N=247,870 SNVs) or v1.1 (N=242,901 SNVs). Illumina’s GenTrain version 2.0 clustering algorithm in GenomeStudio or zCall^48^ was used for genotype calling. Details regarding genotyping and QC for each study are summarized in Supplementary Data 1. To improve accurate calling of rare variants 10 studies comprising *N*=62,666 samples participated in joint calling centrally, which has been described in detail elsewhere^13^. In brief, all samples were combined and genotypes were initially auto-called with the Illumina GenomeStudio v2011.1 software and the GenTrain2.0 clustering algorithm. SNVs meeting best practices criteria^13^ based on call rates, genotyping quality score, reproducibility, heritability and sample statistics were then visually inspected and manually re-clustered when possible. The performance of the joint calling and best practices approach (CHARGE clustering method) was evaluated by comparing exome chip data to available whole-exome sequencing data (N=530 in ARIC). The CHARGE clustering method performed better compared with other calling methods and showed 99.8% concordance between the exome chip and exome sequence data. A total of 8,994 SNVs failed QC across joint calling of studies and were omitted from all analyses. Additional studies used the CHARGE cluster files to call genotypes or used a combination of gencall and zCall^48^. The quality control criteria performed by each study for filtering of poorly genotyped individuals and of low-quality SNVs included a call rate of <0.95, gender mismatch, excess autosomal heterozygosity, and SNV effect estimate s.e. >10^−6^. Concordance rates of genotyping across the exome chip and GWAS platforms were checked in ARIC and FHS and was >99%. After SNV-level and sample-level quality control, 197,481 variants were available for analyses. The minor allele frequency spectrums of the exome chip SNVs by annotation category are depicted in Supplementary Table 22. Cluster plots of *GLP1R* and *ABO* variants are shown in Supplementary Fig. 9.

### Whole-exome sequencing

For exome sequencing analyses we had data from up to 14,118 individuals of European ancestry from seven studies, including four studies contributing exome sequence samples that also participated in the exome chip analyses (Atherosclerosis Risk in Communities Study (ARIC, *N*=2,905), Cardiovascular Health Study (CHS, *N*=645), Framingham Heart Study (FHS, *N*=666) and Rotterdam Study (RS, *N*=702)) and three additional studies, Erasmus Rucphen Family Study (ERF, *N*=1,196), the Exome Sequencing Project (ESP, *N*=1,338) and the GlaxoSmithKline discovery sequence project^3^ (GSK, *N*=6,666). The GlaxoSmithKline (GSK) discovery sequence project provided summary level statistics combining data from GEMS, CoLaus and LOLIPOP collections that added additional exome sequence data at *GLP1R*, including *N*=3,602 samples with imputed genotypes. In all studies sequencing was performed using the Illumina HiSeq 2000 platform. The reads were mapped to the GRCh37 Human reference genome (http://www.ncbi.nlm.nih.gov/projects/genome/assembly/grc/human/) using the Burrows-Wheeler aligner (BWA^49^, http://bio-bwa.sourceforge.net/), producing a BAM^50^ (binary alignment/map) file. In ERF, the NARWHAL pipeline^51^ was used for this purpose as well. In GSK paired-end short reads were aligned with SOAP^52^. GATK^53^ (http://www.broadinstitute.org/gatk/) and Picard (http://picard.sourceforge.net) were used to remove systematic biases and to do quality recalibration. In ARIC, CHS and FHS the Atlas2^54^ suite (Atlas-SNP and Atlas-indel) was used to call variants and produce a variant call file (VCF^55^). In ERF and RS genetic variants were called using the Unified Genotyper Tool from GATK, for ESP the University of Michigan’s multisample SNP calling pipeline UMAKE was used (H.M. Kang and G. Jun, unpublished data) and in GSK variants were called using SOAPsnp^56^. In ARIC, CHS and FHS variants were excluded if SNV posterior probability was <0.95 (QUAL<22), number of variant reads were <3, variant read ratio was <0.1, >99% variant reads were in a single strand direction, or total coverage was <6. Samples that met a minimum of 70% of the targeted bases at × 20 or greater coverage were submitted for subsequent analysis and QC in the three cohorts. SNVs with >20% missingness, >2 observed alleles, monomorphic, mean depth at the site of >500-fold or HWE *P*<5 × 10^−6^ were removed. After variant-level QC, a quality assessment of the final sequence data was performed in ARIC, CHS and FHS based on a number of measures, and all samples with a missingness rate of >20% were removed. In RS, samples with low concordance to genotyping array (< 95%), low transition/transversion ratio (<2.3) and high heterozygote to homozygote ratio (>2.0) were removed from the data. In ERF, low-quality variants were removed using a QUAL<150 filter. Details of variant and sample exclusion criteria in ESP and GSK have been described before^3^,^57^. In brief, in ESP these were based on allelic balance (the proportional representation of each allele in likely heterozygotes), base quality distribution for sites supporting the reference and alternate alleles, relatedness between individuals and mismatch between called and phenotypic gender. In GSK these were based on sequence depth, consensus quality and concordance with genome-wide panel genotypes, among others.

### Phenotyping glycaemic physiologic traits in additional cohorts

We tested association of the lead signal rs10305492 at *GLP1R* with glycaemic traits in the post absorptive state because it has a putative role in the incretin effect. Cohorts with measurements of glucose and/or insulin levels post 75 g oral glucose tolerance test (OGTT) were included in the analysis (see Supplementary Table 2 for list of participating cohorts and sample sizes included for each trait). We used linear regression models under the assumption of an additive genetic effect for each physiologic trait tested.

Ten cohorts (ARIC, CoLaus, Ely, Fenland, FHS, GLACIER, Health2008, Inter99, METSIM, RISC, Supplementary Table 2) provided data for the 2-h glucose levels for a total sample size of 37,080 individuals. We collected results for 2-h insulin levels in a total of 19,362 individuals and for 30 min-insulin levels in 16,601 individuals. Analyses of 2-h glucose, 2-h insulin and 30 min-insulin were adjusted using three models: (1) age, sex and centre; (2) age, sex, centre and BMI; and (3) age, sex, centre, BMI and FG. The main results in the manuscript are presented using model 3. We opted for the model that included FG because these traits are dependent on baseline FG^1^,^58^. Adjusting for baseline FG assures the effect of a variant on these glycaemic physiologic traits are independent of FG.

We calculated the insulinogenic index using the standard formula: [insulin 30 min−insulin baseline]/[glucose 30 min−glucose baseline] and collected data from five cohorts with appropriate samples (total *N*=16,203 individuals). Models were adjusted for age, sex, centre, then additionally for BMI. In individuals with ≥3 points measured during OGTT, we calculated the area under the curve (AUC) for insulin and glucose excursion over the course of OGTT using the trapezoid method^59^. For the analysis of AUC_ins_ (*N*=16,126 individuals) we used three models as discussed above. For the analysis of AUC_ins_/AUC_gluc_ (*N*=16,015 individuals) we only used models 1 and 2 for adjustment.

To calculate the incretin effect, we used data derived from paired OGTT and intra-venous glucose tolerance test (IVGTT) performed in the same individuals using the formula: (AUC_ins_ OGTT-AUC_ins_ IVGTT)/AUC_ins_ OGTT in RISC (*N*=738). We used models 1 and 2 (as discussed above) for adjustment.

We were also able to obtain lookups for estimates of insulin sensitivity from euglycaemic-hyperinsulinemic clamps and from frequently sampled intravenous glucose tolerance test from up to 2,170 and 1,208 individuals, respectively (Supplementary Table 3).

All outcome variables except 2-h glucose were log transformed. Effect sizes were reported as s.d. values using s.d. values of each trait in the Fenland study^60^, the Ely study^61^ for insulinogenic index and the RISC study^62^ for incretin effects to allow for comparison of effect sizes across phenotypes.

### Statistical analyses

The R package seqMeta was used for single variant, conditional and gene-based association analyses^63^ (http://cran.r-project.org/web/packages/seqMeta/). We performed linear regression for the analysis of quantitative traits and logistic regression for the analysis of binary traits. For family-based cohorts linear mixed effects models were used for quantitative traits and related individuals were removed before logistic regression was performed. All studies used an additive coding of variants to the minor allele observed in the jointly called data set^13^. All analyses were adjusted for age, sex, principal components calculated from genome-wide or exome chip genotypes and study-specific covariates (when applicable) (Supplementary Data 1). Models testing FI were further adjusted for BMI^32^. Each study analysed ancestral groups separately. At the meta-analysis level ancestral groups were analysed both separately and combined. Meta-analyses were performed by two independent analysts and compared for consistency. Overall quantile-quantile plots are shown in Supplementary Fig. 10.

Bonferroni correction was used to determine the threshold of significance. In single-variant analyses, for FG and FI, all variants with a MAF>0.02% (equivalent to a MAC≥20; *N*_SNVs_=150,558) were included in single-variant association tests; the significance threshold was set to *P*≤3 × 10^−7^ (*P*=0.05/150,558), corrected for the number of variants tested. For T2D, all variants with a MAF>0.01% in T2D cases (equivalent to a MAC≥20 in cases; *N*_SNVs_=111,347) were included in single-variant tests; the significance threshold was set to *P*≤4.5 × 10^−7^ (*P*=0.05/111,347).

We used two gene-based tests: the Sequence Kernel Association Test (SKAT) and the Weighted Sum Test (WST) using Madsen Browning weights to analyze variants with MAF<1% in genes with a cumulative MAC≥20 for quantitative traits and cumulative MAC≥40 for binary traits. These analyses were limited to stop gain/loss, nsSNV, or splice-site variants as defined by dbNSFP v2.0 (ref. 31). We considered a Bonferroni-corrected significance threshold of *P*≤1.6 × 10^−6^ (0.05/30,520 tests (15,260 genes × 2 gene-based tests)) in the analysis of FG and FI and *P*≤1.7 × 10^−6^ (0.05/29,732 tests (14,866 genes × 2 gene-based tests)) in the analysis of T2D. Owing to the association of multiple rare variants with FG at *G6PC2* from both single and gene-based analyses, we removed one variant at a time and repeated the SKAT test to determine the impact of each variant on the gene-based association effects (Wu weight) and statistical significance.

We performed conditional analyses to control for the effects of known or newly discovered loci. The adjustment command in seqMeta was used to perform conditional analysis on SNVs within 500 kb of the most significant SNV. For *ABO* we used the most significant SNV, rs651007. For *G6PC2* we used the previously reported GWAS variants, rs563694 and rs560887, which were also the most significant SNV(s) in the data analysed here.

The threshold of significance for known FG and FI loci was set at *p*_FG_≤1.5 × 10^−3^ and *p*_FI_<2.9 × 10^−3^ (=0.05/34 known FG loci and=0.05/17 known FI loci). For FG, FI and T2D functional variant analyses the threshold of significance was computed as *P*=1.1 × 10^−5^ (=0.05/4513 protein affecting SNVs at 38 known FG susceptibility loci), *P*=3.9 × 10^−5^ (=0.05/1281 protein affecting SNVs at 20 known FI susceptibility loci), *P*=1.3 × 10^−4^ (=0.05/412 protein affecting SNVs at 72 known T2D susceptibility loci) and *P*=3.5 × 10^−4^ (0.05/(72 × 2)) for the gene-based analysis of 72 known T2D susceptibility loci^2^,^34^. We assessed the associations of glycaemic^1^,^32^,^64^ and T2D^2^,^34^ variants identified by previous GWAS in our population.

We developed a novel meta-analysis approach for haplotype results based on an extension of Zaykin’s method^65^. We incorporated family structure into the basic model, making it applicable to both unrelated and related samples. All analyses were performed in *R*. We developed an *R* function to implement the association test at the cohort level. The general model formula for *K*-observed haplotypes (with the most frequent haplotype used as the reference) is




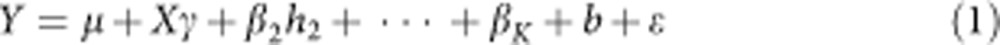




Where *Y* is the trait; *X* is the covariates matrix; *h*_m_(m=2,…, K) is the expected haplotype dosage: if the haplotype is observed, the value is 0 or 1; otherwise, the posterior probability is inferred from the genotypes; *b* is the random intercept accounting for the family structure (if it exists), and is 0 for unrelated samples; 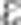 is the random error.

For meta-analysis, we adapted a multiple parameter meta-analysis method to summarize the findings from each cohort^66^. One primary advantage is that this approach allows variation in the haplotype set provided by each cohort. In other words, each cohort could contribute uniquely observed haplotypes in addition to those observed by multiple cohorts.

### Associations of *ABO* variants with cardiometabolic traits

Variants in the *ABO* region have been associated with a number of cardiovascular and metabolic traits in other studies (Supplementary Table 8), suggesting a broad role for the locus in cardiometabolic risk. For significantly associated SNVs in this novel glycaemic trait locus, we further investigated their association with other metabolic traits, including systolic blood pressure (SBP, in mm Hg), diastolic blood pressure (DBP, in mm Hg), body mass index (BMI, in kg m^−2^), waist hip ratio (WHR) adjusted for BMI, high-density lipoprotein cholesterol (HDL-C, in mg dl^−1^), low-density lipoprotein cholesterol (LDL-C, in mg dl^−1^), triglycerides (TG, natural log transformed, in % change units) and total cholesterol (TC, in mg dl^−1^). These traits were examined in single-variant exome chip analysis results in collaboration with other CHARGE working groups. All analyses were conducted using the R packages skatMeta or seqMeta^63^. Analyses were either sex stratified (BMI and WHR analyses) or adjusted for sex. Other covariates in the models were age, principal components and study-specific covariates. BMI, WHR, SBP and DBP analyses were additionally adjusted for age squared; WHR, SBP and DBP were BMI adjusted. For all individuals taking any blood pressure lowering medication, 15 mm Hg was added to their measured SBP value and 10 mm Hg to the measured DBP value. As described in detail previously^8^ in selected individuals using lipid lowering medication, the untreated lipid levels were estimated and used in the analyses. All genetic variants were coded additively. Maximum sample sizes were 64,965 in adiposity analyses, 56,538 in lipid analyses and 92,615 in blood pressure analyses. Threshold of significance was *P*=6.2 × 10^−3^ (*P*=0.05/8, where eight is the number of traits tested).

### Pathway analyses of *GLP1R*

To examine whether biological pathways curated into gene sets in several publicly available databases harboured exome chip signals below the threshold of exome-wide significance for FG or FI, we applied the MAGENTA gene-set enrichment analysis (GSEA) software as previously described using all pathways in the Kyoto Encyclopedia of Genes and Genomes (KEGG), Gene Ontology (GO), Reactome, Panther, BioCarta and Ingenuity pathway databases^67^. Genes in each pathway were scored based on unconditional meta-analysis *P* values for SNVs falling within 40 kb upstream and 110 kb downstream of gene boundaries; we used a 95th percentile enrichment cutoff in MAGENTA, meaning pathways (gene sets) were evaluated for enrichment with genes harbouring signals exceeding the 95th percentile of all genes. As we tested a total of 3,216 pathways in the analysis, we used a Bonferroni-corrected significance threshold of *P*<1.6 × 10^−5^ in this unbiased examination of pathways. To limit the GSEA analysis to pathways that might be implicated in glucose or insulin metabolism, we selected gene sets from the above databases whose names contained the terms ‘gluco,’ ‘glycol,’ ‘insulin’ or ‘metabo.’ We ran MAGENTA with FG and FI data sets on these ‘glucometabolic’ gene sets using the same gene boundary definitions and 95th percentile enrichment cutoff as described above; as this analysis involved 250 gene sets, we specified a Bonferroni-corrected significance threshold of *P*<2.0 × 10^−4^. Similarly, to examine whether genes associated with incretin signalling harboured exome chip signals, we applied MAGENTA software to a gene set that we defined comprised genes with putative biologic functions in pathways common to *GLP1R* activation and insulin secretion, using the same gene boundaries and 95th percentile enrichment cutoff described above (Supplementary Table 4). To select genes for inclusion in the incretin pathway gene set, we examined the ‘Insulin secretion’ and ‘Glucagon-like peptide-1 regulates insulin secretion’ pathways in KEGG and Reactome, respectively. From these two online resources, genes encoding proteins implicated in GLP1 production and degradation (namely glucagon and *DPP4*), acting in direct pathways common to *GLP1R* and insulin transcription, or involved in signalling pathways shared by *GLP1R* and other incretin family members were included in our incretin signalling pathway gene set; however, we did not include genes encoding proteins in the insulin secretory pathway or encoding cell membrane ion channels as these processes likely have broad implications for insulin secretion independent from *GLP1R* signalling. As this pathway included genes known to be associated with FG, we repeated the MAGENTA analysis excluding genes with known association from our gene set—*PDX1*, *ADCY5*, *GIPR* and *GLP1R* itself.

### Protein conformation simulations

The A316T receptor mutant structure was modelled based on the WT receptor structure published previously^22^. First, the Threonine residue is introduced in place of Alanine at position 316. Then, this receptor structure is inserted back into the relaxed membrane-water system from the WT structure^22^. T316 residue and other residues within 5 Å of itself are minimized using the CHARMM force field^68^ in the NAMD^69^ molecular dynamics (MD) programme. This is followed by heating the full receptor-membrane-water to 310 K and running MD simulation for 50 ns using the NAMD program. Electrostatics are treated by E-wald summation and a time step of 1 fs is used during the simulation. The structure snapshots are saved every 1 ps and the fluctuation analysis (Supplementary Fig. 3) used snapshots every 100 ps. The final snapshot is shown in all the structural figures.

### Annotation and functional prediction of variants

Variants were annotated using dbNSFP v2.0 (ref. 31). GTEx (Genotype-Tissue Expression Project) results were used to identify variants associated with gene expression levels using all available tissue types^16^. The Encyclopedia of DNA Elements (ENCODE) Consortium results^14^ were used to identify non-coding regulatory regions, including but not limited to transcription factor binding sites (ChIP-seq), chromatin state signatures, DNAse I hypersensitive sites and specific histone modifications (ChIP-seq) across the human cell lines and tissues profiled by ENCODE. We used the UCSC Genome Browser^15^,^70^ to visualize these data sets, along with the public transcriptome data contained in the browser’s ‘Genbank mRNA’ (cDNA) and ‘Human ESTs’ (Expressed Sequence Tags) tracks, on the hg19 human genome assembly. LncRNA and antisense transcription were inferred by manual annotation of these public transcriptome tracks at UCSC. All relevant track groups were displayed in Pack or Full mode and the Experimental Matrix for each subtrack was configured to display all extant intersections of these regulatory and transcriptional states with a selection of cell or tissue types comprised of ENCODE Tier 1 and Tier 2 human cell line panels, as well as all cells and tissues (including but not limited to pancreatic beta cells) of interest to glycaemic regulation. We visually scanned large genomic regions containing genes and SNVs of interest and selected trends by manual annotation (this is a standard operating procedure in locus-specific in-depth analyses utilizing ENCODE and the UCSC Browser). Only a subset of tracks displaying gene structure, transcriptional and epigenetic data sets from or relevant to T2D, and SNVs in each region of interest was chosen for inclusion in each UCSC Genome Browser-based figure. Uninformative tracks (those not showing positional differences in signals relevant to SNVs or genes of interest) were not displayed in the figures. ENCODE and transcriptome data sets were accessed via UCSC in February and March 2014. To investigate the possible significant overlap between the ABO locus SNPs of interest and ENCODE feature annotations we performed the following analysis. The following data sets were retrieved from the UCSC genome browser: wgEncodeRegTfbsClusteredV3 (TFBS); wgEncodeRegDnaseClusteredV2 (DNase); all H3K27ac peaks (all: wgEncodeBroadHistone*H3k27acStdAln.bed files); and all H3K4me1 peaks (all: wgEncodeBroadHistone*H3k4me1StdAln.bed files). The histone mark files were merged and the maximal score was taken at each base over all cell lines. These features were then overlapped with all SNPs on the exome chip from this study using bedtools (v2.20.1). GWAS SNPs were determined using the NHGRI GWAS catalogue with *P* value<5 × 10^−8^. LD values were obtained by the PLINK program based on the Rotterdam Study for SNPs within 100 kB with an *r*^2^ threshold of 0.7. Analysis of these files was completed with a custom R script to produce the fractions of non-GWAS SNPs with stronger feature overlap than the ABO SNPs as well as the Supplementary Figure.

## Additional information

**How to cite this article**: Wessel, J. *et al*. Low-frequency and rare exome chip variants associate with fasting glucose and type 2 diabetes susceptibility. *Nat. Commun.* 6:5897 doi: 10.1038/ncomms6897 (2015).

## Supplementary Material

Supplementary Information Supplementary Figures 1-10, Supplementary Tables 1-22, Supplementary Notes 1-2 and Supplementary References

Supplementary Data 1 Study characteristics

Supplementary Data 2 Case definitions and sample size stratified by ancestry for all studies contributing to the type 2 diabetes association analyses

Supplementary Data 3 Association of significant FG and FI SNVs with T2D in combined ancestry analyses and stratfied by European and African ancestry

Supplementary Data 4 Association of GLP1R SNVs from whole exome sequencing with fasting glucose by chromosome position

Supplementary Data 5 Single variant association results for rare coding variants (included in gene-based analyses) and common variants at G6PC2 on fasting glucose

Supplementary Data 6 Association of G6PC2 SNVs from whole exome sequencing with FG

Supplementary Data 7 Protein affecting SNVs at known T2D loci associated with T2D in European and combined ancestry analyses

Supplementary Data 8 Protein affecting SNVs at known T2D loci associated with T2D in African ancestry analyses

Supplementary Data 9 Association results from FG and FI analyses for previously published index variants and their proxies at FG and FI susceptibility loci

Supplementary Data 10 Association results from T2D analyses for previously published index variants and their proxies at T2D susceptibility loci

## Figures and Tables

**Table 1:** Novel SNPs associated with fasting glucose in African and European ancestries combined.

**Gene**	**Variation type**	**Chr**	**Build 37 position**	**dbSNPID**	**Alleles**		**African and European**				**Proportion of trait variance explained**
					**Effect**	**Other**	**EAF**	**Beta**	**s.e.**	***P***	
* GLP1R *	A316T	6	39046794	rs10305492	A	G	0.01	−0.09	0.013	3.4 × 10^−12^	0.0003
* ABO *	intergenic	9	136153875	rs651007	A	G	0.20	0.02	0.004	1.3 × 10^−8^	0.0002

EAF, effect allele frequency.

Fasting glucose concentrations were adjusted for sex, age, cohort effects and up to 10 principal components in up to 60,564 (AF *N*=9,664 and EU N=50,900) non-diabetic individuals. Effects are reported per copy of the minor allele. Beta coefficient units are in mmol l^−1^.

**Table 2:** Gene-based associations of *G6PC2* with fasting glucose in African and European ancestries combined.

**Gene**	**Chr: Build 37 position**	**cMAF** *	**SNVs (** ***n*** **)** †	**Weighted sum test (WST)**				**Sequence Kernel Association Test (SKAT)**			
				***P***	***P*** ‡	***P*** §	***P*** ||	***P***	***P*** ‡	***P*** §	***P*** ||
* G6PC2 *	2:169757930-169764491	0.016	15	4.1 × 10^−9^	2.6 × 10^−5^	2.3 × 10^−4^	3.1 × 10^−5^	8.2 × 10^−18^	4.8 × 10^−9^	6.8 × 10^−6^	5.2 × 10^−9^

Fasting glucose concentrations were adjusted for sex, age, cohort effects and up to 10 principal components in up to 60,564 non-diabetic individuals.

^*^cMAF=combined minor allele frequency of all variants included in the analysis.

^†^SNVs(n)=number of variants included in the analysis; variants were restricted to those with MAF<0.01 and annotated as nonsynonymous, splice-site, or stop loss/gain variants.

^‡^*P* value for gene-based test after conditioning on rs563694.

^§^*P* value for gene-based test after conditioning on rs560887.

^||^*P* value for gene-based test after conditioning on rs563694 and rs560887.
