# Supplementary material for: Low-frequency and rare exome chip variants associate with fasting glucose and type 2 diabetes susceptibility
Source: Nat Commun. 2015 Jan 29;6:5897. doi: 10.1038/ncomms6897 (PMC4311266; doi:10.1038/ncomms6897)
Supplement: Supplementary Information — Supplementary Figures 1-10, Supplementary Tables 1-22, Supplementary Notes 1-2 and Supplementary References [file ncomms6897-s1.pdf]

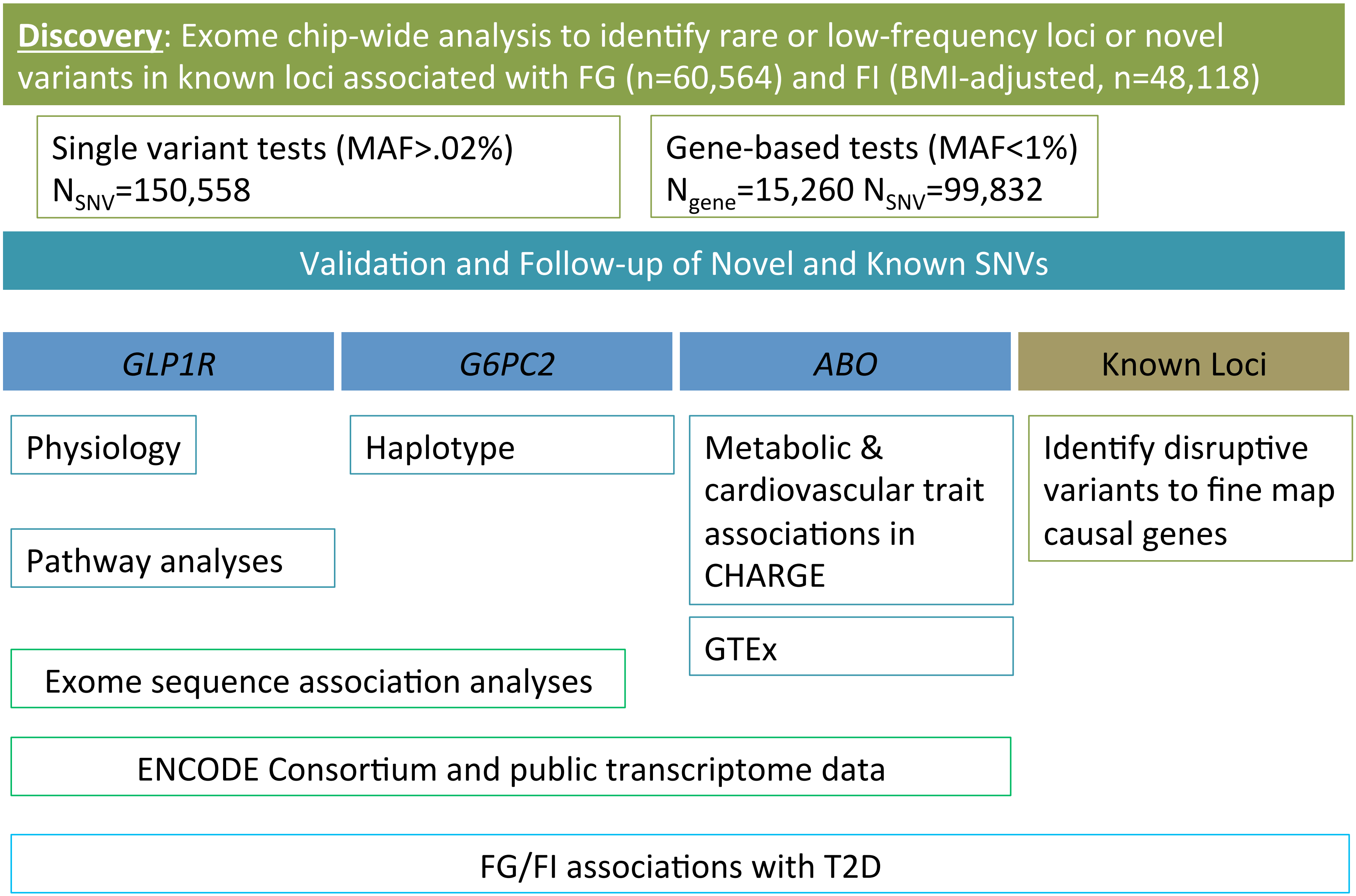

Supplementary Figure 1. Study design. Design of CHARGE consortium discovery of novel variants associated with fasting glucose (FG) and fasting insulin (FI), and the type of follow-up performed on novel and known loci.

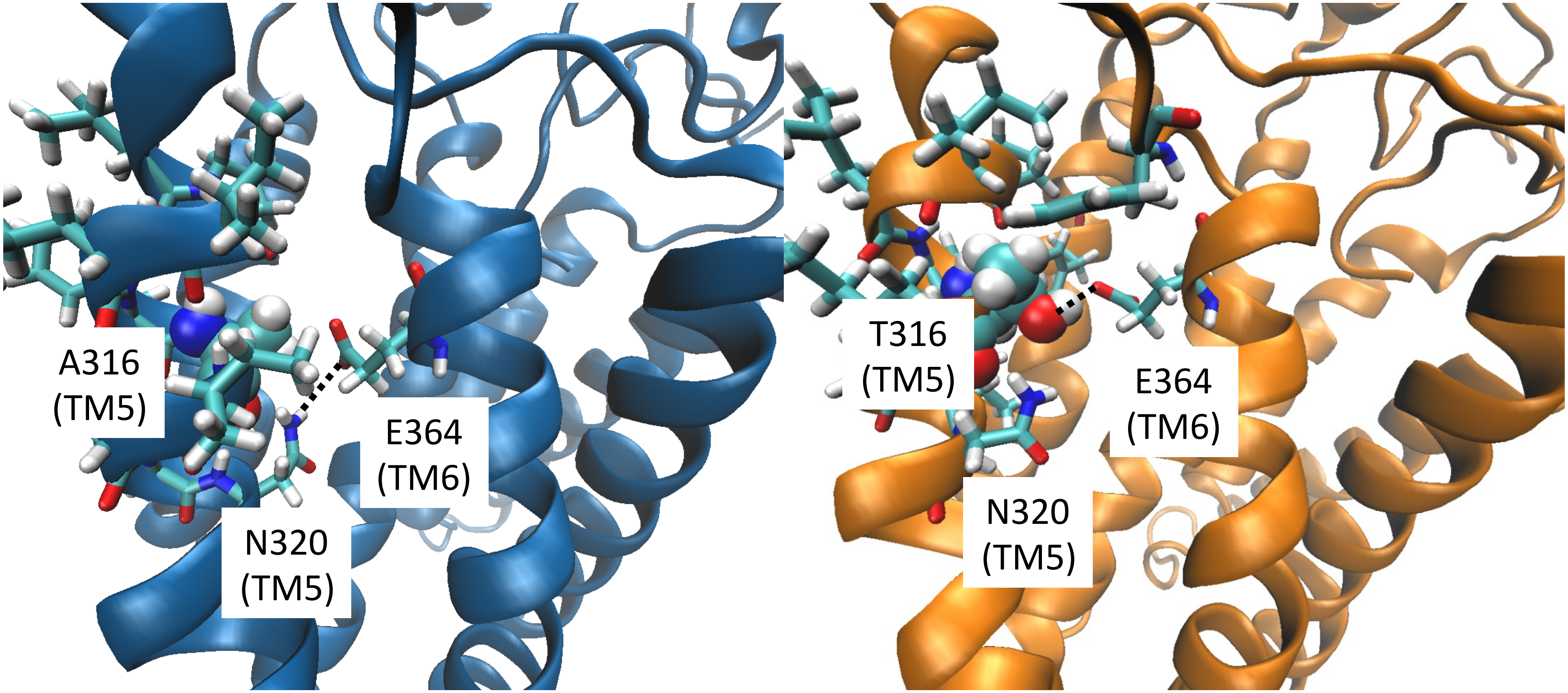

A316 (WT) T316 (mutant)

Supplementary Figure 2. Detailed Comparison of A316 (WT) with T316 (mutant). The figure shows that in the wildtype (WT) receptor (A316), residue N320 (transmembrane (TM) 5) is involved in a hydrogen-bonding interaction with E364 (TM6), whereas in the mutant receptor the T316 residue displaces N320 and takes its place to engage in a stable interaction with E364. These changes then affect the positions of TM5 and TM6, as well as the conformation of the intracellular loop 3 (that connects TM5 and TM6 inside the cell).

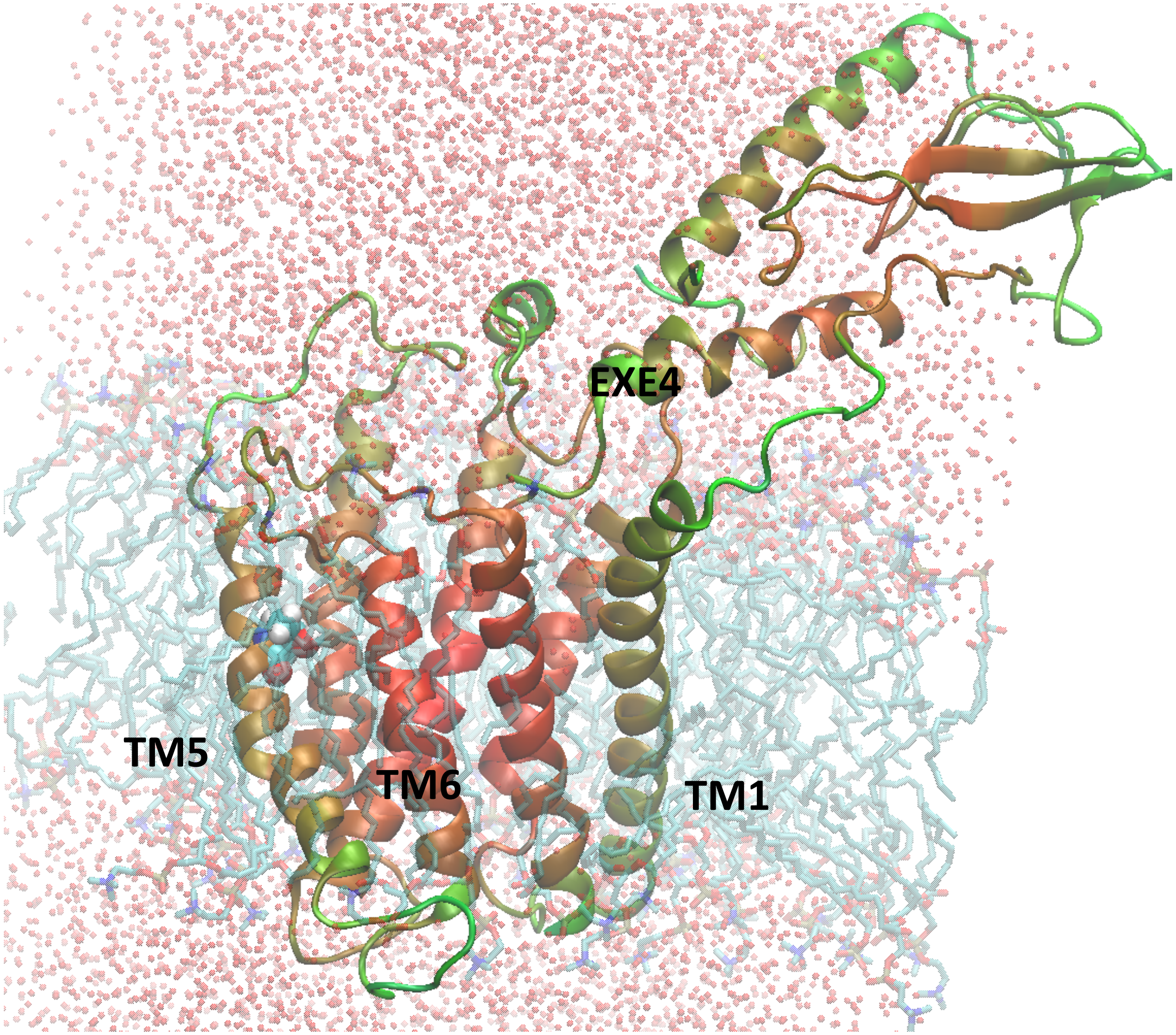

Supplementary Figure 3. Effect of mutant GLP-1 receptor on position in the cell membrane. This figure displays the receptor mutant embedded in the membrane with the receptor color capturing the fluctuations and deviations (red for less and green/blue for more) in the mutant compared to the wild type (WT) receptor. Transmembrane domain 5 (TM5), which contains A316T, and TM1 show the largest changes in conformation/position.

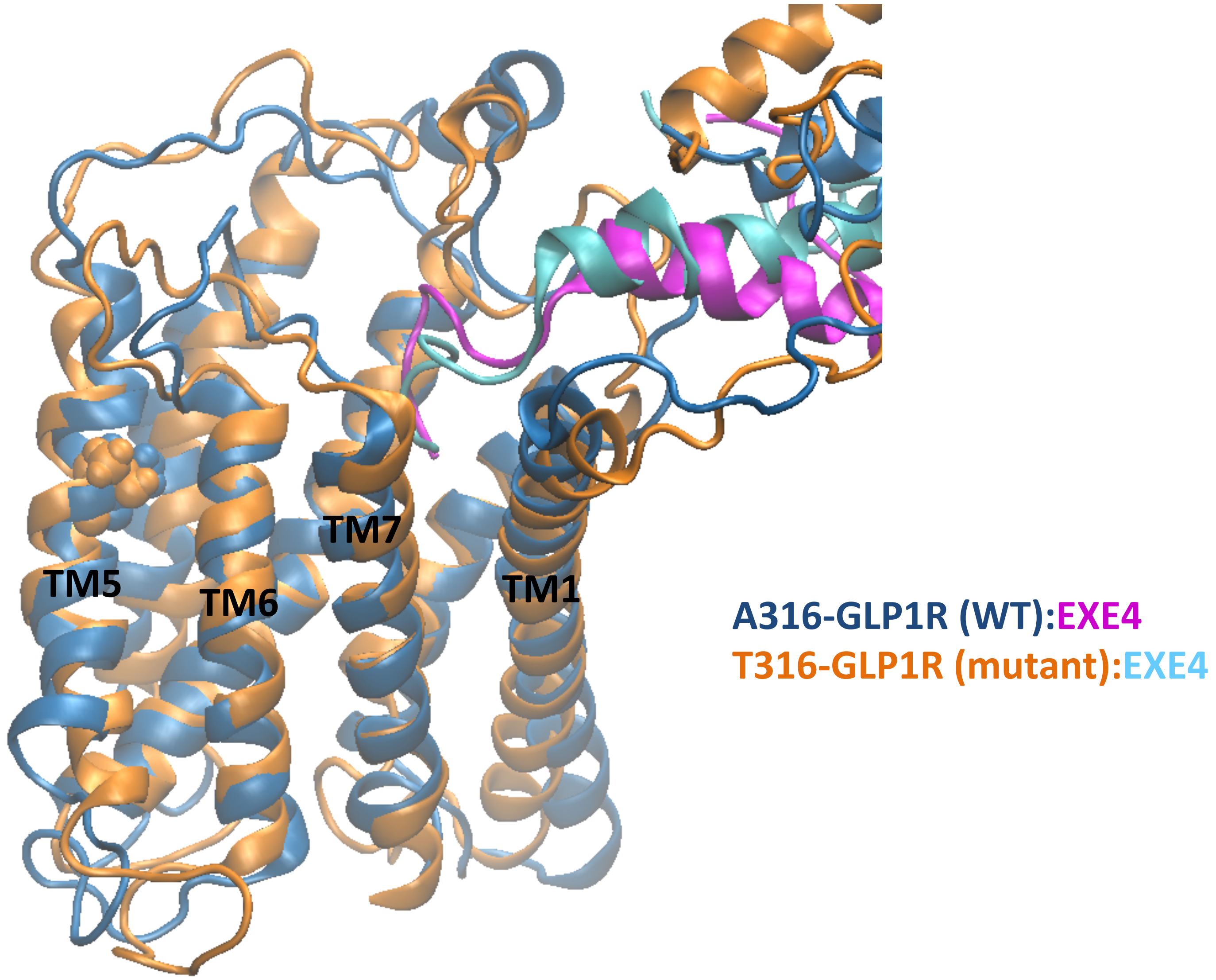

Supplementary Figure 4. Global changes in the transmembrane domains of the mutant GLP1R and exendin-4 (EXE4) system. This figure compares the global changes in the transmembrane (TM) domains for the A316 (WT) receptor and EXE4 (in blue and purple, respectively) versus the T316 (mutant) receptor and EXE4 (in orange and cyan, respectively), showing that TM5 shifts slightly down towards the cytoplasm and TM6 shifts slightly upward.

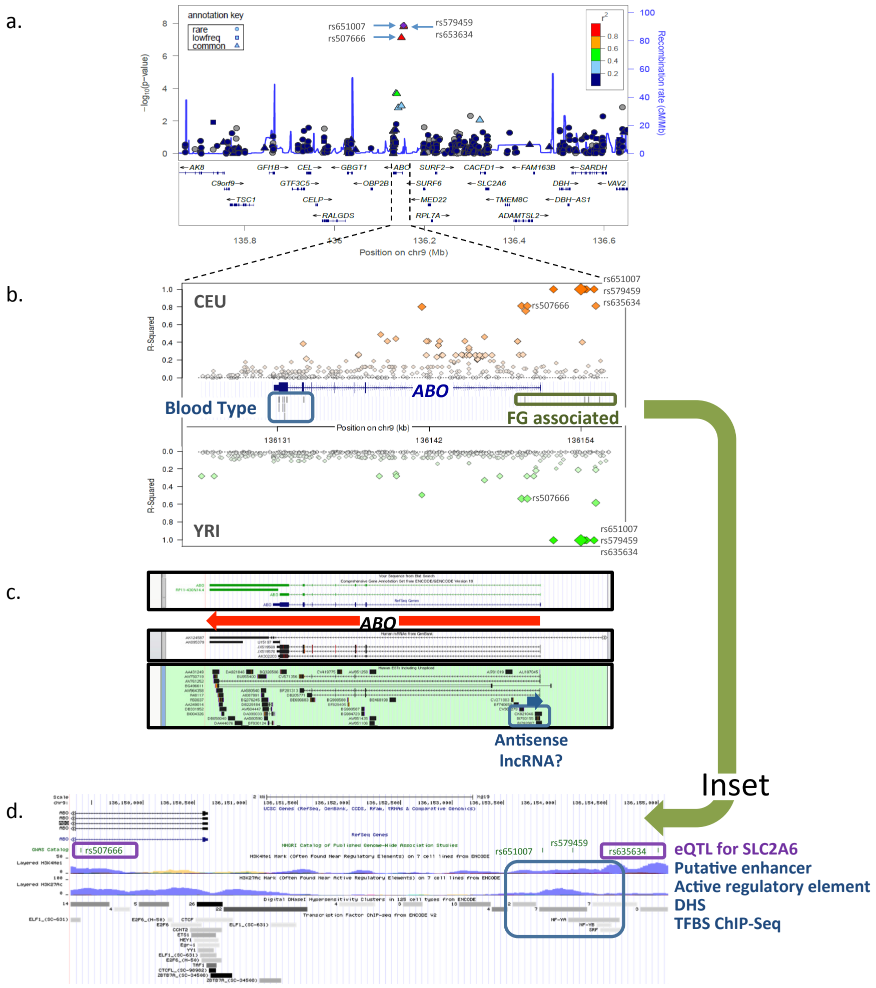

Supplementary Figure 5. Association signals, linkage disequilibrium, transcriptional and epigenetic landscapes of significant SNVs at the *ABO* locus

(**a**) Regional association results ($-\log_{10}p$) for fasting glucose of the *ABO* locus and within 500KB around the lead SNV (rs651007, purple dot); rs579459, rs653634 and rs507666 are also shown and are in strong linkage disequilibrium (LD) with rs651007 (r^2=0.95-1). r^2 indicated by color scale legend. Triangle symbols indicate variants with MAF>5%, square symbols indicate variants with MAF1-5%, and circle symbols indicate variants with MAF <1%.

(**b**) Inset of *ABO* gene with lead SNVs from FG analysis (labeled "FG associated") depicting low LD (r^2) with the major blood group variants (labeled "Blood Type") in European (CEU, top) and African (YRI, bottom) individuals. Major blood group variants were not genotyped on the exome chip; therefore r^2 was calculated from the 1000 Genomes project (Phase 1, version 3).

(**c**) An expressed sequence tag (EST)-supported antisense transcript from islets overlaps *ABO* exon 1. RED arrow: Genomic span of the *ABO* gene. The panel highlighted (light green, below) is the UCSC Expressed Sequence Tags (EST) Track. BLUE arrow: Genomic span of the EST-supported antisense transcript. BLUE Ellipse: ESTs supporting antisense transcription.

(**d**) Inset of the ABO upstream region, promoter,5' untranslated region (5'UTR) and part of intron 1. The intronic SNV rs507666 is near the transcription start site of the expressed sequence tag (EST) CK821046 from a human islet cDNA library. Two other ESTs, also from human islets, support this antisense non-coding transcript. This EST is antisense to exon 1 of *ABO*, suggesting that rs507666 may function as a promoter SNV of a previously uncharacterized *ABO* antisense non-coding RNA transcript in islet cells. The intergenic SNVs rs651007 and rs579459 reside in a DNAseI hypersensitive site cluster, overlapping an H3K27Ac peak and partially overlapping a transcription factor binding site (TFBS) ChIP-seq peak upstream of the *ABO* promoter. The sequences encompassing these SNVs may, therefore, represent putative active chromatin regulatory elements whose function may be altered by these SNVs. The intergenic SNV rs635634 had less evidence for transcriptional or regulatory activity. Purple Boxes: Two variants (rs507666, rs635634) were annotated as eQTLs for *SLC2A6* (gene to the right of *ABO* in panel A) from GTEx analysis. Blue Box: The ENCODE H3K4Me1 (enhancer), H3K27Ac (active regulatory element), DNAse I hypersensitive sites, and TFBS ChIP-Seq tracks, all with signals overlapping the SNVs, are shown.

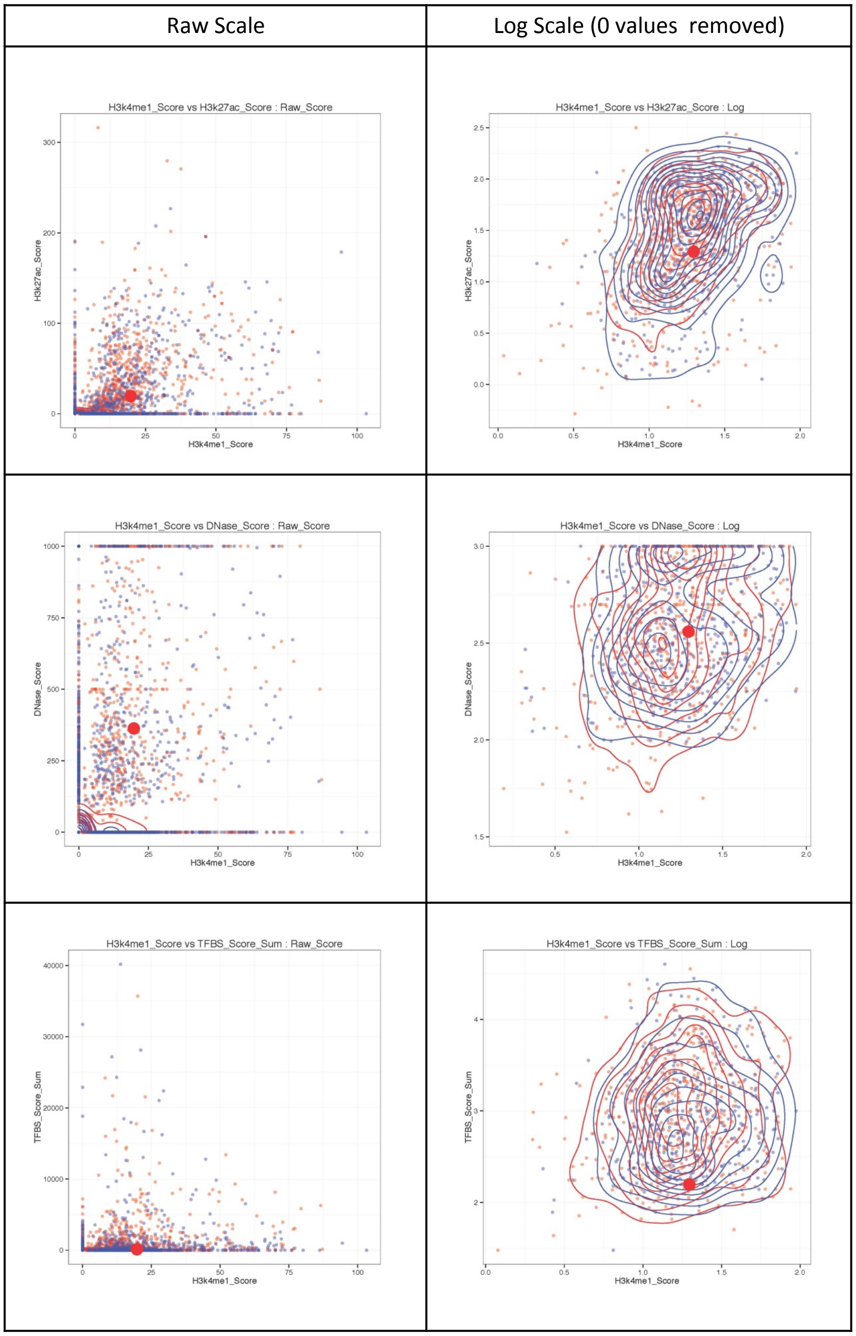

Supplementary Figure 6. ENCODE enrichment analyses. Overlap of *ABO* locus SNP LD block with ENCODE functional marks. Each plot shows the distribution of all SNPs or SNP LD blocks (*ABO* SNP LD block of interest is the larger red point; smaller red points are GWAS SNPs; blue points are non-GWAS SNPs). Each point represents a single SNP or SNP LD block and its location represents the SNPs overlap (score of each mark overlapped or sum for marks with multiple types; i.e. TFBS) with the ENCODE functional mark denoted. Scores are averaged over LD blocks. Each plot is shown in raw and $\log_{10}$ scale. Contour lines are created with the R function kde2d in order to represent the density of all points in each plot.

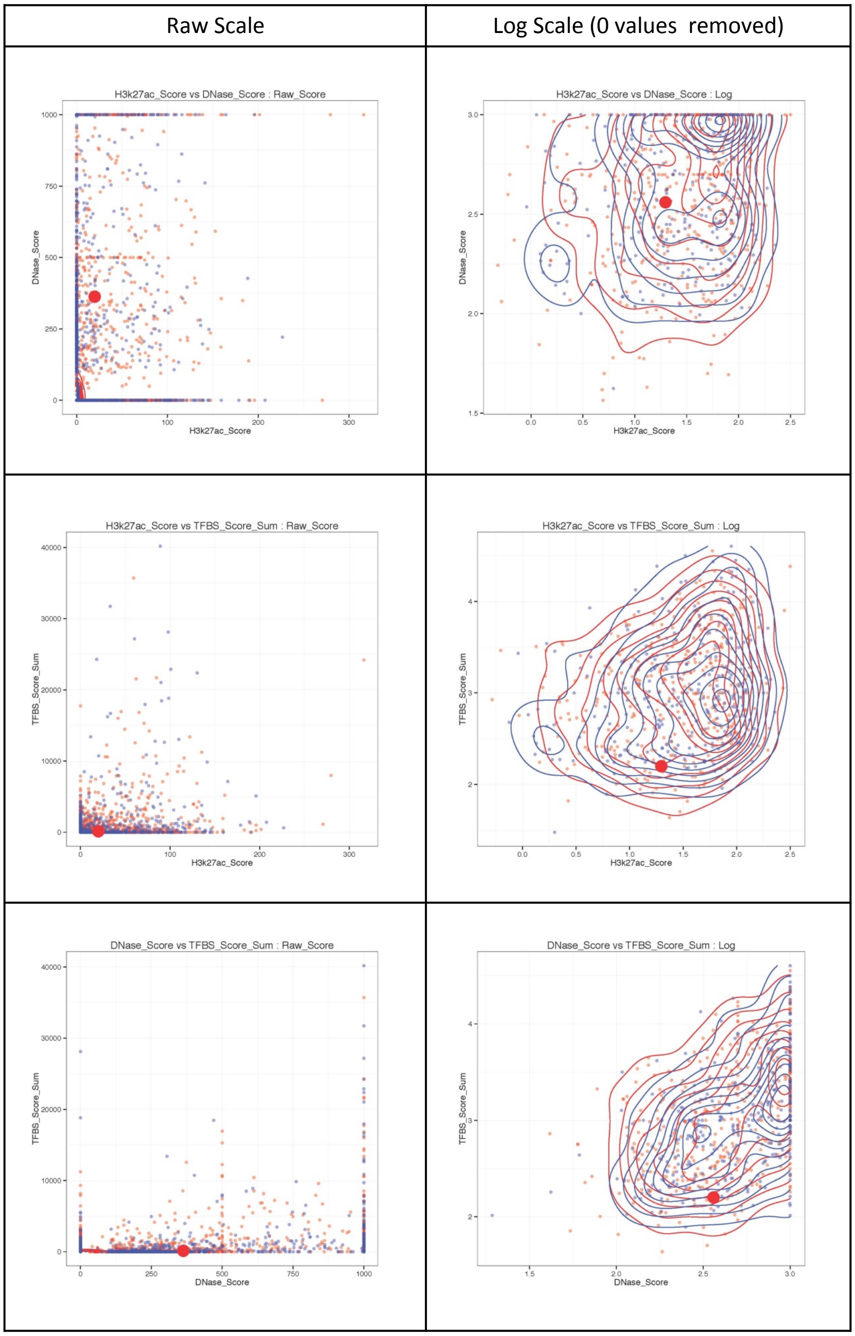

Supplementary Figure 6 (continued). ENCODE enrichment analyses. Overlap of *ABO* locus SNP LD block with ENCODE functional marks. Each plot shows the distribution of all SNPs or SNP LD blocks (*ABO* SNP LD block of interest is the larger red point; smaller red points are GWAS SNPs; blue points are non-GWAS SNPs). Each point represents a single SNP or SNP LD block and its location represents the SNPs overlap (score of each mark overlapped or sum for marks with multiple types; i.e. TFBS) with the ENCODE functional mark denoted. Scores are averaged over LD blocks. Each plot is shown in raw and log_{10} scale. Contour lines are created with the R function kde2d in order to represent the density of all points in each plot.

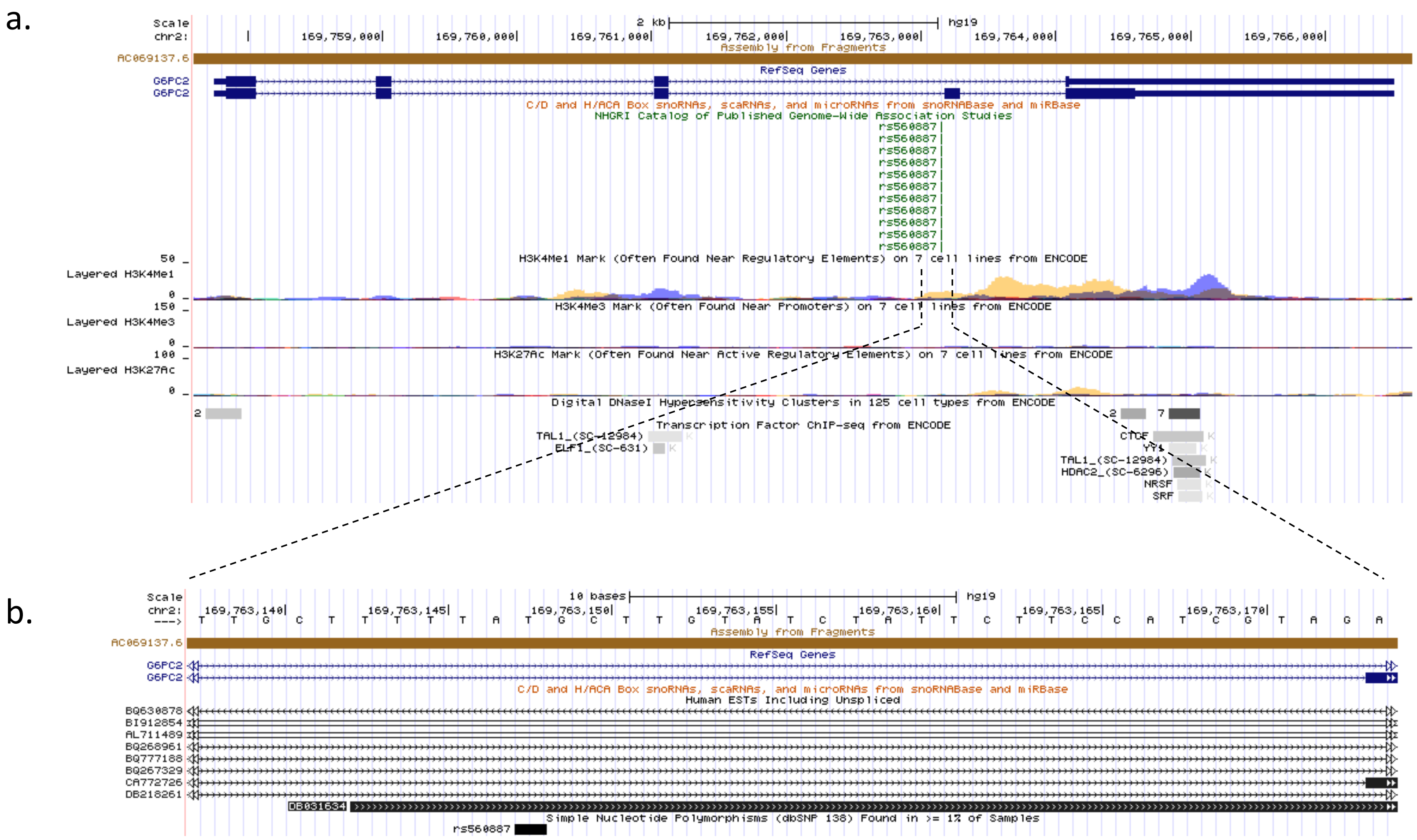

Supplementary Figure 7. The relationship of the common intronic variant rs560887 to epigenetic marks, transcriptional regulation, and splicing at the *G6PC2* locus

(**a**) Figure of *G6PC2* gene structure showing the location of rs560887 and nearby ENCODE epigenetic signatures.

(**b**) Zoomed-in plot showing the EST DB031634 and the splice site of *G6PC2* nearest to rs560887. The intronic SNV rs560887 was assessed as significant in 11 independent NHGRI-catalogued GWAS studies (green, top, A) of serum metabolites, pregnancy-associated glycemia, fasting glucose levels, atherosclerosis, and body mass index (http://genome.ucsc.edu/cgi-bin/hgc?hgsid=369635347_bV0VuuwNQelM7MRwqH5tEyMLIvjx&c=chr2&o=169763147&t=169763148&g=gwasCatalog&i=rs560887). The SNV resides at the 5'end of an ENCODE H3K4Me1 (putative enhancer) signature, suggesting that it may have a regulatory role (Layered H3K4Me1, middle, A). There are several ENCODE transcription factor binding sites evident in ChIP-seq data in the last exon of *G6PC2* (bottom, a), further suggesting that the region may have regulatory functions impacting the expression of *G6PC2* or other genes. The intronic SNV rs560887 is also exonic with respect to the EST DB031634, a positive-strand (same as *G6PC2*) transcript that may represent a cryptic minor isoform of *G6PC2* initiating from an internal promoter in the intron where this SNV resides (b). This SNV is 25 bases upstream of the intron's splice acceptor (b), suggesting that it may also function as a regulator of *G6PC2* splicing.

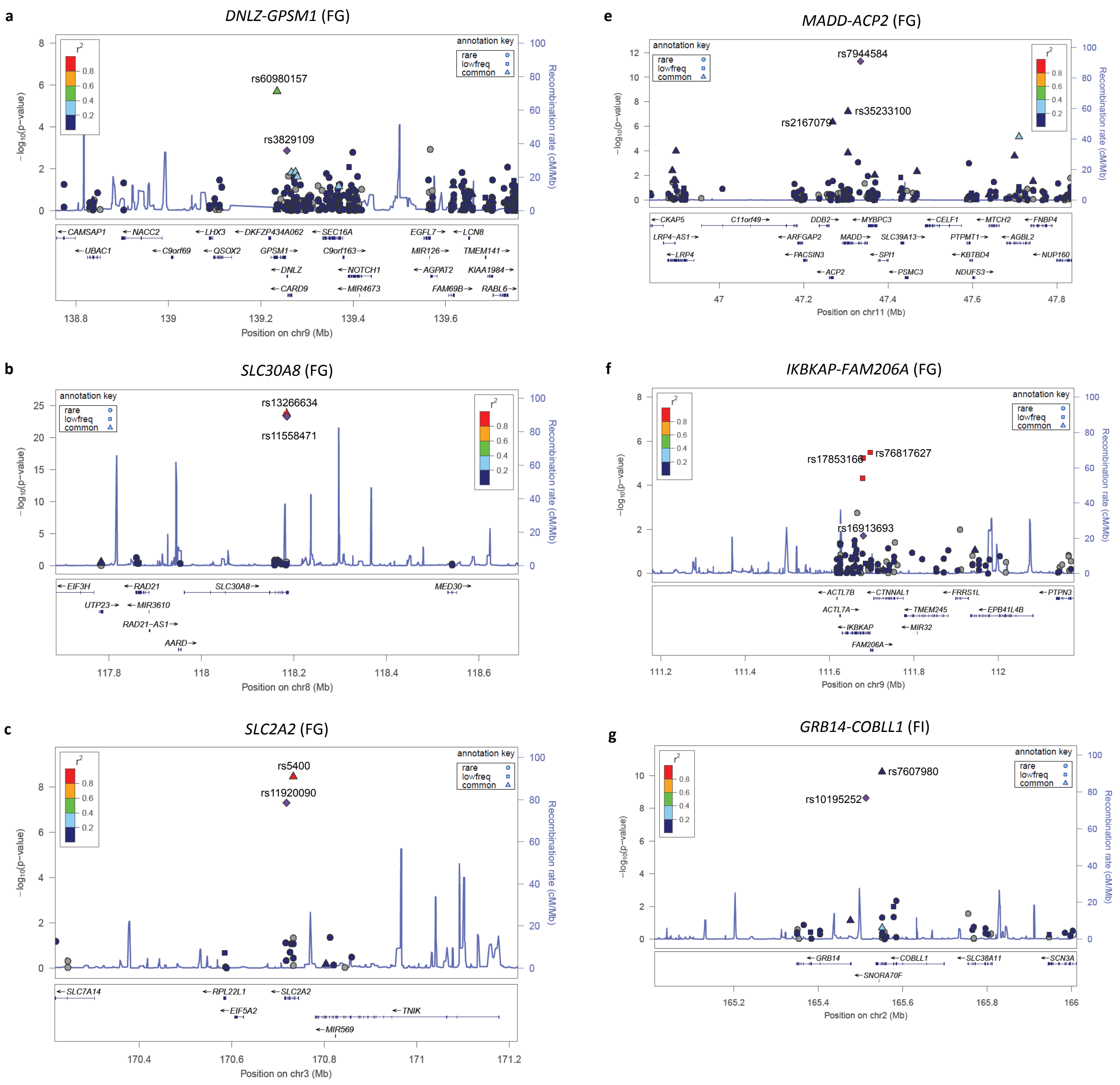

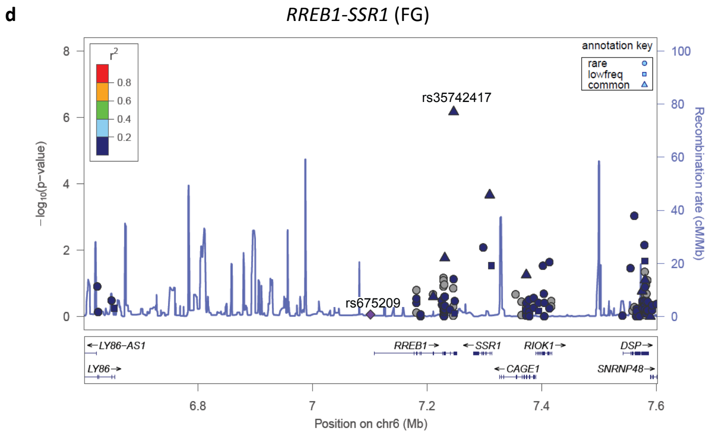

Supplementary Figure 8: Regional association plots for known fasting glucose and fasting insulin loci and including only nonsynonymous, splice or stop-gain/loss variants

Regional association results (-log$_{10}p$) for fasting glucose (FG) (a-f) and fasting insulin (FI) (g).

(a) *DNLZ-GPSM1*

(b) *SLC30A8*

(c) *SLC2A2*

(d) *RREB1*: footnote, rs675209 is the highest quality (but a poor) proxy for the index GWAS FG SNV (rs17762454; r^2=0.46, D'=0.71) available on the Exome chip.

(e) *MADD-ACP2*

(f) *IKBKAP-FAM206A*

(g) *GRB14-COBLL1*

a. rs10305492 – *GLP1R*   b. rs651007 – *ABO*

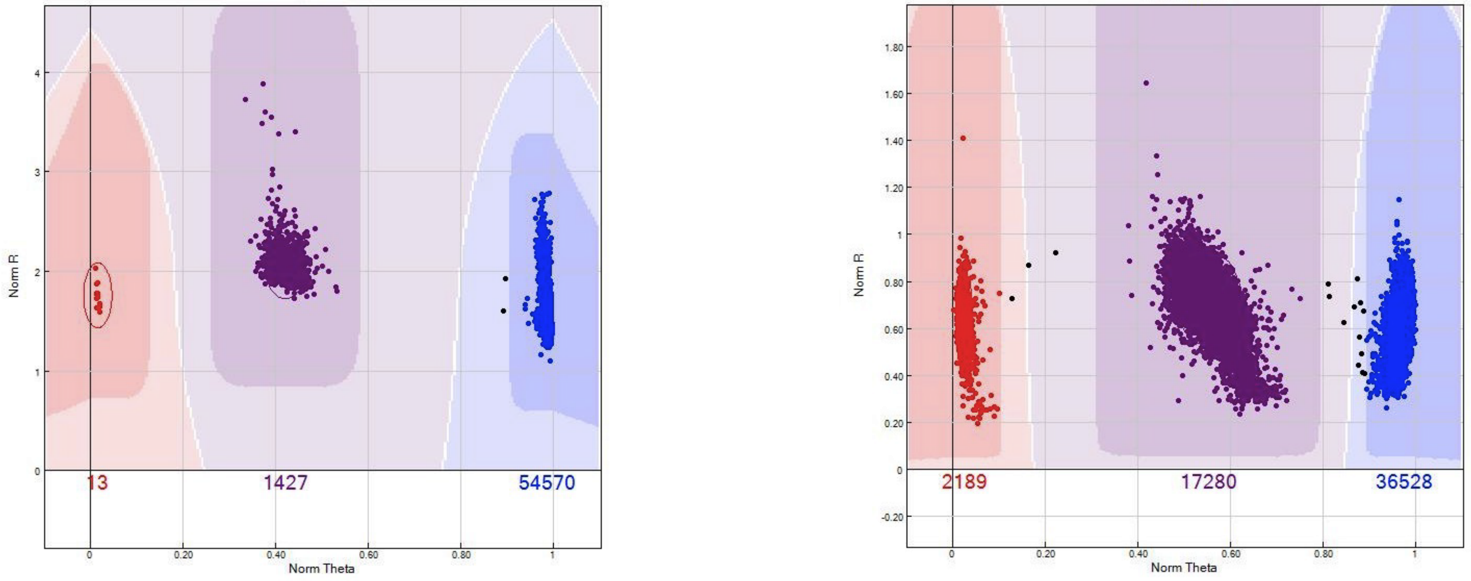

Supplementary Figure 9. Cluster plots of the newly reported variants from CHARGE joint calling.

(a) rs10305492 – *GLP1R* (A316T)

(b) rs651007 – *ABO*

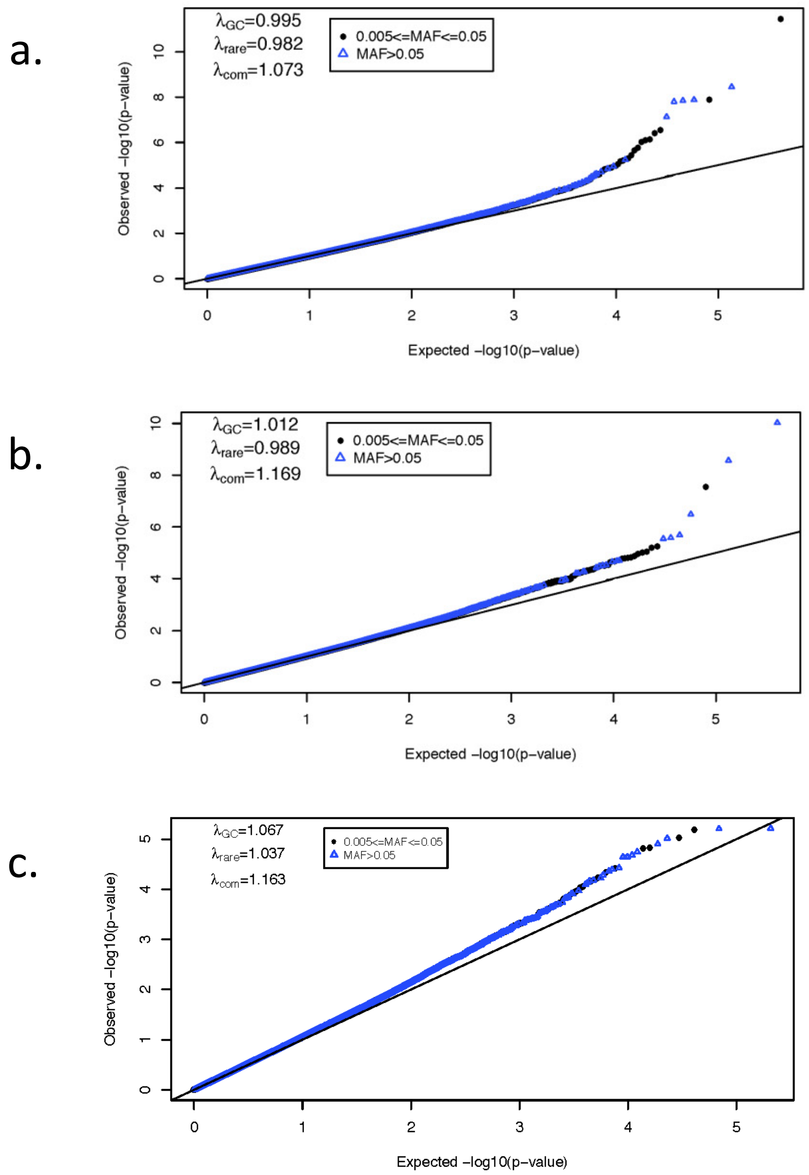

Supplementary Figure 10. Quantile-quantile (QQ) plots from single variant association meta-analysis of (a) fasting glucose, (b) fasting insulin and (c) type 2 diabetes associations without known variants

Supplementary Tables

Supplementary Table 1. Association of novel fasting glucose loci with fasting insulin

Gene	rsID	Chr	Build 37 position	Variation type	Alleles		EAF	Beta	SE	*p*	N
					Effect	Other					
GLP1R	rs10305492	6	39046794	nonsynonymous	A	G	0.015	0.005	0.011	0.67	47388
ABO	rs507666	9	136149399	intronic	A	G	0.177	0.007	0.004	0.05	47388
ABO	rs651007	9	136153875	intergenic	A	G	0.201	0.008	0.003	0.02	47148
ABO	rs579459	9	136154168	intergenic	C	T	0.201	0.008	0.003	0.02	47148
ABO	rs635634	9	136155000	intergenic	A	G	0.177	0.008	0.004	0.03	47148

Fasting insulin concentrations were log transformed and adjusted for sex, age, BMI, cohort effects and up to 10 principal components in up to 48,118 non-diabetic individuals. Effects are reported per copy of the minor allele. EAF: Effect allele frequency; N: sample size

Supplementary Table 2. Sample sizes from cohorts participating in the glycemic physiologic trait analyses

	Sample sizes for traits derived from oral glucose tolerance test (OGTT)						paired IVGTT and OGTT
Cohorts	2h-glucose	2h-insulin	30min insulin	insulinogenic index	AUC insulin	ratio of AUC ins/ AUC gluc	
Ely	1392	1377	1361	1345	1303	1217	NA
Fenland	6319	NA	NA	NA	NA	NA	NA
CoLaus	498	NA	NA	NA	NA	NA	NA
FHS	5716	2625	NA	NA	NA	NA	NA
ARIC	6707	NA	NA	NA	NA	NA	NA
GLACIER	916	NA	NA	NA	NA	NA	NA
Health2008	608	611	594	573	593	589	NA
Inter99	5419	5268	5210	4979	4872	4854	NA
METSIM	8230	8221	8189	8113	8182	8181	NA
RISC	1275	1260	1247	1193	1176	1174	738
total N	37080	19362	16601	16203	16126	16015	738

Glycemic physiologic traits were tested for association with *GLP1R* A316T rs10305492. See methods for estimation of glycemic trait measurements.

Supplementary Table 3. Association of *GLP1R* A316T and insulin sensitivity

SI from frequently sampled IV glucose tolerance test*				Standardized M/I from clamp	
IRAS study (n=184)**		IRAS Family study (n=1,024)***		RISC & ULSAM studies (n=2,170)	
Beta (SE)	*p*	Beta (SE)	*p*	Beta (SD)	*p*
0.51 (0.3)	0.13	-0.23 (0.2)	0.11	-0.04 (-0.25, 0.17)	0.71

Results are presented per minor allele. *Log transformed. **African-Americans. ***Hispanic-Americans

Supplementary Table 4. Genes included in the *GLP1R* pathway for MAGENTA gene-set enrichment analysis

Gene								
ADCY1	*ADCY5*	*ADCY9*	*CALM2*	*GCG*	*GLP2R*	*PCLO*	*PRKX*	*VIP*
ADCY2	*ADCY6*	*ADCYAP1*	*CALM3*	*GIP*	*GNAS*	*PDX1*	*RAB3A*	*VIPR1*
ADCY3	*ADCY7*	*ADCYAP1R1*	*CREB1*	*GIPR*	*ITPR3*	*PRKACA*	*RAPGEF3*	*VIPR2*
ADCY4	*ADCY8*	*CALM1*	*DPP4*	*GLP1R*	*MZB1*	*PRKACB*	*RIMS2*	*WFS1*

Set of 36 genes were defined as having putative biological functions in a pathway between GLP1R activation and insulin secretion.

Supplementary Table 5. Annotation descriptions of exome sequence SNVs for *GLP1R and G6PC2* in all SNVs and stratified by MAF<1% and MAF≥1%

Variation type	*GLP1R*			*G6PC2*		
	All MAF	MAF<1%	MAF≥1%	All MAF	MAF<1%	MAF≥1%
Nonsynonymous	34	30	4	33	30	3
Splicing	0	0	0	1	1	0
Stopgain	2	2	0	4	4	0
Synonymous	29	24	5	9	9	0
Intronic	81	61	20	19	16	3
3' UTR	3	3	0	2	1	1
5' UTR	1	1	0	0	0	0
TOTAL	150	121	29	68	61	7

MAF: minor allele frequency; UTR: untranslated region

Supplementary Table 6. Novel ABO SNPs associated with fasting glucose in African and European ancestries combined and stratified by ancestry

rsID	Alleles		Combined ancestry analysis					European ancestry analysis					African ancestry analysis				
	Effect	Other	EAF	Beta	SE	*p*	p_{cond}[a]	EAF	Beta	SE	*p*	p_{cond}[a]	EAF	Beta	SE	*p*	p_{cond}[a]
rs507666	A	G	0.17	0.02	0.004	7.4E-08	0.28	0.19	0.02	0.004	4.0E-07	0.60	0.10	0.03	0.015	0.02	0.11
rs651007	A	G	0.20	0.02	0.004	1.3E-08	NA	0.21	0.02	0.004	5.8E-08	NA	0.14	0.02	0.013	0.09	NA
rs579459	C	T	0.20	0.02	0.004	2.6E-08	0.15	0.21	0.02	0.004	6.5E-08	0.25	0.14	0.02	0.013	0.10	0.38
rs635634	A	G	0.17	0.02	0.004	1.4E-08	0.17	0.19	0.02	0.004	1.9E-07	0.46	0.11	0.03	0.014	0.02	0.07

Fasting glucose concentrations were adjusted for sex, age, cohort effects and up to 10 principal components in up to 60,564 (African ancestry n=9664 and European ancestry n=50,900) non-diabetic individuals. Effects are reported per copy of the minor allele. Beta coefficient units are in mmol/L. EAF: Effect allele frequency. a Conditional p-value; variants near the ABO locus were conditioned on the most significant SNP in the region (rs651007).

Supplementary Table 7. Associations of *ABO* variants genotyped on the HumanExome BeadChip with FG in combined European and African ancestries

Gene	rsID	Chr	Build 37 Position	Variation type	Alleles		EAF	N	Beta	SE	*p*	p_{cond}[a]	Proxy[b]
					Effect	Other							
ABO	rs7466899	9	136131069	nonsynonymous	A	G	0.001	59748	0.077	0.047	1.0E-01	0.07	
ABO	rs201604341	9	136131119	nonsynonymous	A	G	0.000	59748	0.085	0.250	7.3E-01	0.85	
ABO	rs8176749	9	136131188	nonsynonymous	T	C	0.089	59748	-0.001	0.006	9.2E-01	0.03	Y
ABO	rs8176746	9	136131322	synonymous	A	C	0.089	59748	0.000	0.006	9.3E-01	0.03	Y
ABO	rs8176745	9	136131347	nonsynonymous	A	G	0.246	59016	-0.007	0.004	4.4E-02	0.28	
ABO	rs35494115	9	136131389	nonsynonymous	A	G	0.001	59748	-0.075	0.058	1.9E-01	0.24	
ABO	rs201439325	9	136131407	nonsynonymous	A	G	0.000	59748	-0.103	0.131	4.3E-01	0.20	
ABO	rs8176741	9	136131461	nonsynonymous	A	G	0.089	59748	0.000	0.006	1.0E+00	0.02	Y
ABO	rs8176740	9	136131472	nonsynonymous	T	A	0.246	59748	-0.007	0.004	4.0E-02	0.31	Y
ABO	rs55764262	9	136131539	nonsynonymous	G	A	0.000	57931	0.056	0.131	6.7E-01	0.92	
ABO	rs55727303	9	136131576	nonsynonymous	T	C	0.017	57615	-0.003	0.012	7.8E-01	0.68	
ABO	rs7853989	9	136131592	nonsynonymous	C	G	0.109	49364	0.004	0.006	4.6E-01	0.47	Y
ABO	rs201567722	9	136131629	nonsynonymous	T	C	0.000	59748	0.034	0.090	7.0E-01	0.91	
ABO	rs55756402	9	136131630	nonsynonymous	A	G	0.001	59748	0.079	0.057	1.6E-01	0.92	
ABO	rs200932155	9	136131635	nonsynonymous	A	G	0.001	57615	0.067	0.053	2.0E-01	0.36	
ABO	rs8176738	9	136131636	nonsynonymous	T	C	0.002	59748	0.003	0.037	9.3E-01	0.31	
ABO	rs1053878	9	136131651	nonsynonymous	A	G	0.094	59748	-0.005	0.006	4.1E-01	0.34	Y
ABO	rs201105186	9	136131740	nonsynonymous	A	G	0.000	59748	0.023	0.075	7.6E-01	0.91	
ABO	rs8176721	9	136132852	nonsynonymous	A	G	0.023	59748	-0.018	0.012	1.4E-01	0.25	
ABO	rs8176720	9	136132873	nonsynonymous	C	T	0.363	59748	-0.005	0.003	1.1E-01	0.92	Y
ABO	rs512770	9	136133506	nonsynonymous	A	G	0.217	59748	-0.004	0.004	2.9E-01	0.30	Y
ABO	rs56335272	9	136135236	nonsynonymous	T	C	0.002	59748	0.057	0.043	1.9E-01	0.14	
ABO	rs549446	9	136135238	nonsynonymous	T	C	0.257	59748	-0.007	0.004	3.6E-02	0.27	
ABO	rs688976	9	136136770	nonsynonymous	A	C	0.258	59748	-0.007	0.004	3.6E-02	0.26	
ABO	rs8176696	9	136136773	nonsynonymous	T	C	0.022	59748	-0.013	0.011	2.3E-01	0.20	
ABO	rs687621	9	136137065	intronic	C	T	0.348	59748	0.012	0.003	2.0E-04	0.10	
ABO	rs55876802	9	136137547	nonsynonymous	A	C	0.019	59748	0.013	0.011	2.2E-01	0.38	
ABO	rs55917063	9	136137554	nonsynonymous	T	C	0.003	59748	-0.081	0.034	1.5E-02	0.02	
ABO	rs657152	9	136139265	intronic	T	G	0.366	59748	0.012	0.003	2.1E-04	0.39	
ABO	rs514659	9	136142203	intronic	C	A	0.351	59748	0.010	0.003	1.5E-03	0.39	
ABO	rs505922	9	136149229	intronic	C	T	0.334	59748	0.011	0.003	1.2E-03	0.39	
ABO	rs507666	9	136149399	intronic	A	G	0.173	59748	0.022	0.004	**7.4E-08**	0.28	
ABO-SURF6	rs651007	9	136153875	intergenic	A	G	0.196	59502	0.022	0.004	**1.3E-08**	NA	
ABO-SURF6	rs579459	9	136154168	intergenic	C	T	0.196	59502	0.022	0.004	**1.6E-08**	0.15	
ABO-SURF6	rs635634	9	136155000	intergenic	A	G	0.172	59502	0.023	0.004	**1.4E-08**	0.17	

Fasting glucose concentrations were adjusted for sex, age, cohort effects and up to 10 principal components in up to 60,564 non-diabetic individuals of African and European ancestry. Effects are reported per copy of the minor allele. Beta coefficient units are in mmol/L. Bolded p-values meet significance threshold for single variant analysis. EAF: effect allele frequency; N: sample size. [a] Conditional p-value; variants near the ABO locus were conditioned on the most significant SNP in the region (rs651007). [b] Variant is a proxy for one of the major blood group alleles A_1, A_2, B or O

Supplementary Table 8. Pleiotropic associations at the *ABO* locus from previously reported studies

rsID	Trait	Effect Allele	Reported effect (Beta or OR)	Reported *p*	r^2	Reference
rs651007*	sE-selectin	T	-17.23	1.2E-44	1	1
rs579459*	CAD (OR)	C	1.1	4.1E-14	1	2
rs651007*	TC	T	2.3	8.7E-21	1	2
rs579459*	TC	C	1.72	3.8E-03	1	2
rs651007	LDL-C	A	2.2026	9.8E-09	1	3
rs579459*	LDL-C	C	1.54	4.9E-03	1	3
rs649129	LDL-C	T	2.24	6.0E-13	1	4
rs495828	RBC	T	-0.091	3.3E-12	1	4
rs495828	Hb	T	-0.089	1.2E-11	1	4
rs495828	Ht	T	-0.081	6.1E-10	1	4
rs8176746	MCHC	T	0.084	4.3E-08	0.01	5
rs612169	FAaP	G	NR	9.1E-40	0.51	6
rs507666*	sICAM-1	A	-17.3	3.0E-91	0.96	6
rs514659	disposition index	C	-0.09	3.8E-09	0.53	7

CAD: coronary artery disease, OR: odds ratio, TC: total cholesterol, LDL-C: low-density lipoprotein cholesterol, RBC: red blood cell, Hb:hemoglobin concentration, Ht: hematocrit, MCHC: mean corpuscular hemoglobin concentration, FAaP: fibrinogen A-alpha phosphorylation, NR: not reported, sICAM-1: soluable ICAM1. *Variants associated with fasting glucose in Table 1 and Supplementary Table 10. r^2 is between rs651007 and each SNP listed the first column.

Supplementary Table 9. Lookups of ABO top hits in adiposity, lipid, and blood pressure traits

Adiposity (N_{max}=64,965)

rsID	Alleles		BMI women			BMI men			WHR women			WHR men		
	Effect	Other	Beta	SE	*p*	Beta	SE	*p*	Beta	SE	*p*	Beta	SE	*p*
rs507666	A	G	0.021	0.01	4.0E-03	-0.006	0.01	0.53	-0.011	0.01	0.18	0.001	0.01	0.93
rs651007	A	G	0.025	0.01	3.1E-04	-0.004	0.01	0.62	-0.010	0.01	0.23	0.003	0.01	0.77
rs579459	C	T	0.025	0.01	3.5E-04	-0.004	0.01	0.61	-0.010	0.01	0.24	0.003	0.01	0.80
rs635634	A	G	0.020	0.01	6.0E-03	-0.005	0.01	0.60	-0.010	0.01	0.22	0.002	0.01	0.86

Lipids (N_{max}=56,538)

rsID	Alleles		HDL-C			LDL-C			TG			TC		
	Effect	Other	Beta	SE	*p*	Beta	SE	*p*	Beta*	SE	*p*	Beta	SE	*p*
rs507666	A	G	0.103	0.11	0.36	2.594	0.30	1.9E-18	-0.001	0.00	0.73	2.748	0.33	5.0E-17
rs651007	A	G	0.052	0.11	0.63	2.276	0.28	6.1E-16	-0.001	0.00	0.88	2.397	0.33	3.4E-13
rs579459	C	T	0.052	0.11	0.63	2.274	0.28	6.5E-16	-0.001	0.00	0.88	2.394	0.33	3.6E-13
rs635634	A	G	0.097	0.11	0.40	2.594	0.30	1.9E-18	-0.002	0.00	0.61	2.681	0.35	1.0E-14

Blood pressure (N_{max}=92,615)

rsID	Alleles		DBP			SBP		
	Effect	Other	Beta	SE	*p*	Beta	SE	*p*
rs507666	A	G	-0.153	0.07	2.5E-02	-0.011	0.11	0.92
rs651007	A	G	-0.096	0.07	0.14	0.016	0.11	0.88
rs579459	C	T	-0.095	0.07	0.15	0.022	0.11	0.84
rs635634	A	G	-0.144	0.07	3.5E-02	0.003	0.11	0.98

Fasting lipid concentrations were used. Individuals on lipid or blood pressure lowering medication had their individual values adjusted, see Methods for details. Analyses were adjusted for sex (adiposity was stratified by sex), BMI (for WHR), age, cohort effects and up to 10 principal components. Effects are reported per copy of the minor allele. Beta coefficient units are in kg/m^2 for BMI, mg/dL for lipids and mmHg for blood pressure. BMI: body mass index, WHR: waist-hip ratio, HDL-C: high-density lipoprotein cholesterol, LDL-C: low-density lipoprotein cholesterol, TG: triglycerides, TC: total cholesterol, DBP: diastolic blood pressure, SBP: systolic blood pressure. *Triglycerides are log transformed.

Supplementary Table 10. Associations with *ABO* SNVs and eQTLs from the GTEx database

Gene Id	Gene Symbol	rsID	*p*	Tissue
ENSG00000160326.9	***SLC2A6***	rs507666	1.1E-04	Whole_Blood
ENSG00000175164.8	***ABO***	rs651007	5.9E-05	Whole_Blood
ENSG00000175164.8	***ABO***	rs579459	6.7E-05	Whole_Blood
ENSG00000160326.9	***SLC2A6***	rs635634	1.1E-04	Whole_Blood

Supplementary Table 11. Gene based association results for *G6PC2* removing sets of rare SNVs

cMAF	SNVs(n)	p_{SKAT}	Variant in SKAT gene-based test
0.014	4	8.3E-18	Y177H, S207Y, R283X, P324S
0.003	11	0.36	Removing 4 above

SKAT gene-based test with and without the 4 significant variants identified in single variant analyses. Initital gene-based tests (Table 2) used 15 rare SNVs (MAF<0.01) and annotated as nonsynonymous, splice-site, or loss/gain-of-function variants. cMAF: cumulative minor allele frequency; SNVs(n): number of SNVs in gene-based SKAT test; p_{SKAT}: p-value from gene-based SKAT analysis

Supplementary Table 12. Association of fasting glucose and *G6PC2* haplotypes of 15 rare SNVs

rs142189264	rs149874491	rs201561079	rs199682245	rs187707963	rs2232322	rs145050507	rs138726309	rs2232323	rs145217135	rs147360987	rs150538801	rs148689354	rs146779637	rs2232326	15 rare SNVs (Overall p-value=1.1e-17)		
S30F	I38I	I63T	N68I	Y124C	I171V	I171T	H177Y	Y207S	I230T	H250Y	F256L	I273V	R283X	S324P	N study	Beta	*p*
C	A	T	A	A	A	T	C	A	T	C	T	A	T	T	17	-0.22	2.84E-10
C	A	T	A	A	A	T	C	A	T	C	T	A	C	C	13	-0.26	1.40E-07
C	A	T	A	A	A	T	C	C	T	C	T	A	C	T	18	-0.11	1.45E-06
C	A	T	A	A	A	T	T	C	T	C	T	A	C	T	3	-0.89	0.005
C	A	T	NA	A	A	T	T	A	T	C	T	A	T	T	1	1.31	0.005
C	A	T	A	A	A	T	T	A	T	C	T	A	C	T	16	-0.09	0.021
T	A	T	A	A	A	T	C	A	T	C	T	A	C	T	10	-0.22	0.029
C	A	T	A	A	G	T	C	A	T	C	T	A	C	T	7	0.22	0.134
C	A	T	A	A	A	T	T	A	T	C	T	A	C	T	1	-0.52	0.139
C	C	T	A	A	A	T	C	A	T	C	T	A	C	T	3	-0.19	0.140
C	A	C	A	A	A	T	C	A	T	C	T	A	C	T	2	0.57	0.216
C	A	T	A	A	A	T	C	A	T	C	C	A	C	T	11	-0.13	0.220
C	A	T	A	G	A	T	C	A	T	C	T	A	C	T	1	-0.48	0.407
C	A	T	A	NA	A	T	C	A	C	C	T	A	C	T	1	0.91	0.417
C	A	T	A	A	A	C	C	A	T	C	T	A	C	T	11	-0.07	0.435
C	A	T	A	A	A	T	C	C	T	C	T	A	C	C	1	-1.10	0.438
C	A	T	A	A	A	T	T	A	T	C	T	A	C	C	1	-0.73	0.592
T	A	T	A	A	A	T	C	A	T	C	T	A	C	C	1	0.21	0.645
C	A	T	T	A	A	T	C	A	T	C	T	A	C	C	3	-0.21	0.700
C	A	T	NA	A	A	T	C	A	C	C	T	A	C	T	1	0.10	0.833
C	A	T	A	A	A	T	C	A	T	C	T	A	C	T	18	NA	NA

18 cohorts contributed data. NA is the reference haplotype. Yellow highlighted alleles are the minor allele. N study: number of studies contributing the haplotype observed.

Supplementary Table 13. Association of *G6PC2* haplotypes of 15 rare SNVs and one common SNV (rs560887) with fasting glucose

rs142189264	rs149874491	rs201561079	rs199682245	rs187707963	rs560887	rs2232322	rs145050507	rs138726309	rs2232323	rs145217135	rs147360987	rs150538801	rs148689354	rs146779637	rs2232326	15 rare SNVs plus rs560887 (Overall p-value=1.5e-81)		
S30F	I38I	I63T	N68I	Y124C	NA	I171V	I171T	H177Y	Y207S	I230T	H250Y	F256L	I273V	R283X	S324P	N study	Beta	*p*
C	A	T	A	A	T	A	T	C	A	T	C	T	A	C	T	18	-0.08	8.92E-77
C	A	T	A	A	T	A	T	C	A	T	C	T	A	T	T	17	-0.24	5.63E-12
C	A	T	A	A	T	A	T	C	C	T	C	T	A	C	T	18	-0.13	7.70E-09
C	A	T	A	A	C	A	T	C	A	T	C	T	A	C	C	13	-0.28	2.02E-08
C	A	T	A	A	T	A	T	T	C	T	C	T	A	C	T	3	-0.95	0.003
C	A	T	NA	A	T	A	T	T	A	T	C	T	A	T	T	1	1.29	0.006
C	A	T	A	A	C	A	T	T	A	T	C	T	A	C	T	16	-0.11	0.007
T	A	T	A	A	C	A	T	C	A	T	C	T	A	C	T	10	-0.28	0.010
C	A	T	A	A	C	A	T	C	A	T	C	C	A	C	T	11	-0.19	0.10
C	A	T	A	A	C	G	T	C	A	T	C	T	A	C	T	6	0.32	0.17
C	C	T	A	A	C	A	T	C	A	T	C	T	A	C	T	3	-0.18	0.23
C	A	C	A	A	C	A	T	C	A	T	C	T	A	C	T	2	0.51	0.26
C	A	T	A	A	T	A	T	T	A	T	C	T	A	C	T	9	-2.51	0.30
C	A	T	A	G	C	A	T	C	A	T	C	T	A	C	T	1	-0.53	0.36
C	A	T	T	A	C	A	T	C	A	T	C	T	A	C	T	1	-0.45	0.37
C	A	T	A	A	T	A	C	C	A	T	C	T	A	C	T	7	-0.38	0.40
C	A	T	A	A	T	A	T	C	C	T	C	T	A	C	C	1	-1.13	0.43
C	A	T	A	A	C	A	C	C	A	T	C	T	A	C	T	9	-0.08	0.43
C	A	T	A	NA	C	A	T	C	A	C	C	T	A	C	T	1	0.86	0.44
C	A	T	T	A	T	A	T	C	A	T	C	T	A	C	T	1	-0.96	0.53
C	A	T	A	A	C	A	T	T	A	T	C	T	A	C	C	1	-0.78	0.57
C	C	T	A	A	T	A	T	C	A	T	C	T	A	C	T	1	-200	0.57
C	A	T	A	A	C	A	T	C	C	T	C	T	A	C	T	4	-0.32	0.58
C	A	T	T	A	C	A	T	C	A	T	C	T	A	C	C	3	-0.25	0.64
C	A	T	A	A	T	A	T	C	A	T	C	T	A	C	C	8	-0.22	0.66
T	A	T	A	A	C	A	T	C	A	T	C	T	A	C	C	1	0.16	0.71
C	A	T	A	A	T	A	T	C	A	T	C	C	A	C	T	4	0.13	0.74
C	A	T	A	A	C	A	T	C	A	T	C	T	A	T	T	2	-619118	0.76
C	A	T	A	A	T	G	T	C	A	T	C	T	A	C	T	5	0.06	0.79
T	A	T	A	A	T	A	T	C	A	T	C	T	A	C	T	6	0.11	0.81
C	A	T	NA	A	C	A	T	C	A	C	C	T	A	C	T	1	0.05	0.91
C	A	T	A	A	C	A	T	C	A	T	C	T	A	C	T	18	NA	NA

18 cohorts contributed data. NA is the reference haplotype. Yellow highlighted alleles are the minor allele. N study: number of studies contributing the haplotype observed.

Supplementary Table 14. Gene based association results for *G6PC2* and fasting insulin

Ancestry	cMAF	SNVs(n)	p_{WST}	p_{SKAT}	N
Combined ancestry	0.02	17	0.11	0.53	47388
European ancestry only	0.02	14	0.45	0.55	38528
African ancestry only	0.01	13	0.75	0.76	8860

Analyses adjusted for sex, age, cohort effects and up to 10 principal components in up to 47,388 in the combined ancestry analysis, 38,528 in the European ancestry analysis, and 8860 in the African ancestry analysis. SNVs(n), number of variants included in the analysis; variants were restricted to those with MAF<0.01 and annotated as nonsynonymous splice-site, or loss/gain-of-function variants. cMAF, cumulative MAF: combined minor allele frequency of all variants included in the analysis; pWST: p-value from weighted sum test (WST); pSKAT: p-value from sequence kernal association test (SKAT); N: sample size.

Supplementary Table 15. Gene based association results for *G6PC2* and type 2 diabetes

Ancestry	cMAF	SNVs(n)	p_{WST}	p_{SKAT}	N
Combined ancestry	0.019	18	0.75	0.68	34984
European ancestry only	0.019	16	0.49	0.52	17651
African ancestry only	0.010	15	0.60	0.60	3814

Analyses adjusted for sex, age, cohort effects and up to 10 principal components in up to 16,491 T2D cases and 81,877 controls in the combined ancestry analysis, 10,240 T2D cases and 63,105 controls in the European ancestry analysis, and 3,097 T2D cases and 10,326 controls in the African ancestry analysis. cMAF, cumulative MAF: combined minor allele frequency of all variants included in the analysis. SNVs(n), number of variants included in the analysis; variants were restricted to those with MAF<0.01 and annotated as nonsynonymous, splice-site, or loss/gain-of-function variants. pWST: p-value from weighted sum test (WST); pSKAT: p-value from sequence kernal association test (SKAT); N: sample size.

Supplementary Table 16. Gene based association results for *G6PC2* and fasting glucose from exome sequence analyses in up to 7,452 individuals of European ancestry

Gene	cMAF	SNVs(n)	p_{WST}	p_{SKAT}
G6PC2	0.027	36	5.4E-04	1.4E-03
G6PC2 (exome chip variants)	0.018	10	3.2E-03	1.3E-03
G6PC2 (excluding exome chip variants)	0.009	26	4.0E-02	6.1E-01

cMAF, cumulative MAF: combined minor allele frequency of all variants included in the analysis; SNVs(n): number of variants included in the analysis; pWST: p-value from weighted sum test (WST). pSKAT: p-value from sequence kernal association test (SKAT). Variants were restricted to those with MAF<0.01 and annotated as nonsynonymous splice-site, or loss/gain-of-function variants.SNVs(n)=36 variants met criteria for inclusion in gene based tests. SNVs(n)=10 are the same variants available on the exome chip. SNVs(n)=26 are the the variants available for analyses after excluding the 10 above.

Supplementary Table 17. Top ten pathways with lowest p-values in MAGENTA analysis of FG, analyzing all genes (A) and excluding those with known associations with FG (B)

A.

Database	Pathway name	GSEA p-value	FDR
GOTERM	Positive regulation of DNA replication	9.80E-05	1.33E-01
KEGG	Glioma	3.00E-04	4.80E-02
GOTERM	Pancreas development	5.00E-04	2.24E-01
PANTHER	Insulin/IGF pathway, protein kinase B signaling cascade	1.00E-03	1.16E-01
REACTOME	Signal attenuation	1.00E-03	1.43E-01
KEGG	Citrate cycle, TCA cycle	1.70E-03	4.63E-02
INGENUITY	IGF-1 signaling	1.80E-03	6.32E-02
PANTHER	DNA repair	1.80E-03	4.61E-01
KEGG	Acute myeloid leukemia	1.90E-03	5.21E-02
KEGG	Type 2 diabetes mellitus	2.20E-03	5.84E-02

B.

Database	Pathway name	GSEA p-value	FDR
KEGG	Glioma	1.00E-04	1.59E-02
GOTERM	Positive regulation of DNA replication	1.12E-04	1.68E-01
KEGG	Acute myeloid leukemia	1.50E-03	5.40E-02
KEGG	Focal adhesion kinase	1.60E-03	1.12E-01
KEGG	Non-homologous end joining	2.80E-03	4.50E-02
KEGG	Citrate cycle, TCA cycle	3.00E-03	6.63E-02
BIOCARTA	IGF1 pathway	3.00E-03	3.08E-01
GOTERM	Centrosome organization	3.20E-03	5.03E-01
GOTERM	Oligodendrocyte development	3.70E-03	6.88E-01
REACTOME	Cell cycle, mitotic	3.80E-03	7.37E-01

Supplementary Table 18. Top ten pathways with lowest p-values in MAGENTA analysis of FI, analyzing all genes (A) and excluding those with known associations with FI (B)

A.

Database	Pathway name	GSEA p-value	FDR
GOTERM	Response to DNA damage stimulus	9.80E-05	1.33E-01
REACTOME	Regulation of IGF activity by IGF binding proteins	3.00E-04	4.80E-02
GOTERM	ATP binding	5.00E-04	2.23E-01
GOTERM	Solute:hydrogen antiporter activity	1.00E-03	1.16E-01
REACTOME	PECAM1 interactions	1.00E-03	1.44E-01
GOTERM	Dephosphorylation	1.70E-03	4.63E-02
GOTERM	Positive regulation of smooth muscle contraction	1.80E-03	6.32E-02
BIOCARTA	ATRBRCA pathway	1.80E-03	4.61E-01
GOTERM	Rab GTPase binding	1.90E-03	5.21E-02
GOTERM	Nucleotide binding	2.20E-03	5.84E-02

B.

Database	Pathway name	GSEA p-value	FDR
GOTERM	Response to DNA damage stimulus	1.00E-04	1.59E-02
GOTERM	ATP binding	1.12E-04	1.68E-02
REACTOME	PECAM1 interactions	1.50E-03	5.40E-02
GOTERM	Protein C-terminus binding	1.60E-03	1.12E-01
GOTERM	Solute:hydrogen antiporter activity	2.80E-03	4.50E-02
GOTERM	Dephosphorylation	3.00E-03	6.63E-01
GOTERM	Positive regulation of smooth muscle contraction	3.00E-03	3.08E-01
GOTERM	Motor activity	3.20E-03	5.03E-01
GOTERM	Rab GTPase binding	3.70E-03	6.88E-01
GOTERM	Cation transport	3.80E-03	7.37E-01

Supplementary Table 19. MAGENTA results for glucometabolic pathways from curated pathway databases for FG (A) and FI (B)

A.

Database	Pathway name	GSEA p-value	FDR
GOTERM	Cellular metabolic process	3.50E-03	6.55E-02
PANTHER	Metabolism of cyclic nucleotides	1.67E-02	4.33E-01
GOTERM	Positive regulation of insulin secretion	1.77E-02	1.36E-01
REACTOME	Glucose and other sugar SLC transporters	2.83E-02	1.10E-01
KEGG	Alpha linoleic acid metabolism	3.88E-02	5.79E-01
PANTHER	Lipid, fatty acid and steroid metabolism	4.17E-02	4.19E-01
GOTERM	Hydrolase activity, hydrolyzing O-glycosyl compounds	5.24E-02	3.66E-01
GOTERM	Response to glucose stimulus	5.45E-02	2.90E-01
PANTHER	Phospholipid metabolism	5.60E-02	5.65E-01
REACTOME	Metabolism of carbohydrates	5.62E-02	1.00E+00

B.

Database	Pathway name	GSEA p-value	FDR
REACTOME	Regulation of IGF activity by IGF binding proteins	8.00E-04	2.30E-03
GOTERM	Xenobiotic metabolic process	6.60E-03	1.49E-01
GOTERM	Regulation of lipid metabolic process	1.59E-02	1.50E-01
GOTERM	Lipid metabolic process	3.73E-02	5.13E-01
KEGG	Inositol phosphate metabolism	5.22E-02	1.00E+00
GOTERM	Response to glucose stimulus	5.48E-02	2.96E-01
GOTERM	Generation of precursor metabolites and energy	6.08E-02	4.87E-01
GOTERM	Response to glucocorticoid stimulus	6.25E-02	2.35E-01
GOTERM	Glucose homeostasis	7.08E-02	1.73E-01
REACTOME	Peroxisomal lipid metabolism	7.21E-02	8.74E-01

Supplementary Table 20. Identifying coding variants significantly associated with fasting glucose and fasting insulin in known loci

Gene	Index*/novel	rsID	Chr	Build 37 position	Alleles		Variation type	EAF	Beta	SE	*p*	N	r^2	D'
					Effect	Other								
**<u>Fasting Glucose</u>**														
DNLZ	index	rs3829109	9	139256766	A	G	intronic	0.26	-0.01	0.004	1.4E-03	55633	LD with rs3829109	
GPSM1	nsSNV	rs60980157	9	139235415	T	C	nonsynonymous	0.24	-0.02	0.004	2.0E-06	55633	0.62	0.88
SLC30A8	index	rs11558471	8	118185733	G	A	UTR3	0.28	-0.04	0.003	5.5E-24	59748	LD with rs11558471	
SLC30A8	nsSNV	rs13266634	8	118184783	T	C	nonsynonymous	0.27	-0.04	0.004	1.7E-24	59748	0.97	1.00
SLC2A2	index	rs11920090	3	170717521	A	T	intronic	0.17	-0.02	0.004	5.0E-08	59748	LD with rs11920090	
SLC2A2	nsSNV	rs5400	3	170732300	A	G	nonsynonymous	0.18	-0.03	0.004	3.5E-09	59748	1.00	1.00
													LD with rs17762454*	
	"proxy"	rs675209	6	7102084	T	C	intergenic	0.29	0.00	0.003	8.7E-01	59502	0.46	0.71
RREB1	novel	rs35742417	6	7247344	A	C	nonsynonymous	0.18	-0.02	0.004	6.9E-07	59748	0.04	0.79
MADD	index	rs7944584	11	47336320	T	A	intronic	0.24	-0.03	0.004	5.1E-12	59748	LD with rs7944584	
MADD	nsSNV	rs35233100	11	47306630	T	C	stopgain	0.05	-0.04	0.007	6.2E-08	59748	0.13	1.00
ACP2	novel	rs2167079	11	47270255	T	C	nonsynonymous	0.34	0.02	0.003	4.5E-07	59748	0.15	0.93
AGBL2	novel	rs7941404	11	47712213	T	C	nonsynonymous	0.12	-0.02	0.005	7.0E-06	59748	0.20	0.73
IKBKAP	index	rs16913693	9	111680359	G	T	intronic	0.06	-0.02	0.007	1.9E-02	59748	LD with rs16913693	
FAM206A	novel	rs76817627	9	111696795	T	C	nonsynonymous	0.02	-0.05	0.010	3.2E-06	59748	1.00	1.00
IKBKAP	novel	rs17853166	9	111679940	C	T	nonsynonymous	0.02	-0.05	0.010	5.7E-06	59748	1.00	1.00
**<u>Fasting Insulin</u>**														
	index	rs10195252	2	165513091	C	T	intergenic	0.48	-0.02	0.003	2.3E-09	46332	LD with rs10195252	
COBLL1	index	rs7607980	2	165551201	C	T	nonsynonymous	0.13	-0.03	0.004	5.9E-11	47388	0.15	0.83

Causal gene for *MADD* and *IKBKAP* is undetermined since associations are seen in multiple genes and the SNVs r2<.2. Fasting glucose concentrations were adjusted for sex, age, cohort effects and up to 10 principal components in up to 60,564 (African ancestry n=9664 and European ancestry n=50,900) non-diabetic individuals. Effects are reported per copy of the minor allele. Beta coefficient units are in mmol/L. *No proxy for index (rs17762454) is on the exome chip. p-value threshold: 1.1x10-5=0.05/4513 SNVs analyzed. EAF: effect allele frequency; N: Sample size, UTR: untranslated region; nsSNV:nonsynonymous single nucleotide variant.

Supplementary Table 21. Association of MODY variants with fasting glucose and fasting insulin

Trait	Gene	rsID	Chr	Build 37 Position	Alleles		Variation type	EAF	Beta	SE	*p*	N
					Effect	Other						
FG	*KLF11*	rs34336420	2	10188123	T	C	nonsynonymous	0.007	-0.013	0.021	0.529	59748
	HNF4A	rs139591750	20	43047151	G	A	synonymous	0.002	-0.109	0.036	0.003	59502
FI	*KLF11*	rs34336420	2	10188123	T	C	nonsynonymous	0.008	-0.009	0.019	0.656	47388
	HNF4A	rs139591750	20	43047151	G	A	synonymous	0.003	-0.062	0.033	0.063	47148

Fasting glucose concentrations were adjusted for sex, age, cohort effects and up to 10 principal components in up to 60,564 non-diabetic individuals; beta coefficient units are in mmol/L. Fasting insulin concentrations were log transformed and adjusted for sex, age, BMI, cohort effects and up to 10 principal components in up to 48,118 non-diabetic individuals. Effects are reported per copy of the minor allele. EAF: effect allele frequency; N: sample size.

Supplementary Table 22. Type of variant on the exome chip by allele frequency

Type	All MAF	MAF<1%	MAF>=1%
nonsynonymous	128679	32142	96537
intergenic	8142	8116	26
intronic	5573	5536	37
synonymous	3827	1332	2495
splicing, synonymous	1788	494	1294
splicing, nonsynonymous	2387	491	1896
ncRNA intronic	435	434	1
UTR3	482	431	51
splicing	1079	307	772
stopgain	1862	289	1573
downstream	181	178	3
upstream	177	176	1
ncRNA_exonic	101	92	9
UTR5	69	64	5
exonic;stoploss	125	33	92
exonic;splicing	39	15	24
upstream;downstream	8	8	0
ncRNA_UTR3	7	7	0
exonic;splicing;stopgain	31	4	27
exonic;splicing;stoploss	3	1	2
ncRNA_splicing	1	1	0
ncRNA_UTR5	1	1	0
TOTAL Disruptive	134205	33282	100923
TOTAL	154997	50152	104845

Up to 155,106 SNVs were available for association analyses of fasting glucvose and insulin.The type of variant is uncategorized for 109 SNVs. MAF: minor allele frequency.

Supplementary Note 1. The EPIC-InterAct Consortium

Claudia Langenberg(1), Robert A Scott(1), Stephen J Sharp(1), Nita G Forouhi(1), Nicola D Kerrison(1), Debora ME Lucarelli (1), Matt Sims(1), Inês Barroso(2,3), Panos Deloukas(2), Mark I McCarthy(4,5,6), Larraitz Arriola(7,8,9), Beverley Balkau(10,11), Aurelio Barricarte(12,9), Matthias B Schulze (13), Heiner Boeing(13), Paul W Franks(14,15), Carlos Gonzalez(16), Sara Grioni(17), Rudolf Kaaks(18), Timothy J Key(19), Carmen Navarro(20,9,21), Peter M Nilsson(14), Kim Overvad(22,23), Domenico Palli(24), Salvatore Panico(25), J. Ramón Quirós(26), Olov Rolandsson(15), Carlotta Sacerdote(27,28), María-José Sánchez(29,9,30), Nadia Slimani(31), Anne Tjonneland(32), Rosario Tumino(33,34), Daphne L van der A(35), Yvonne T van der Schouw(36), Elio Riboli(37), Nicholas J Wareham(1)

(1) MRC Epidemiology Unit, University of Cambridge, Cambridge, United Kingdom, (2) The Wellcome Trust Sanger Institute, Cambridge, United Kingdom, (3) University of Cambridge Metabolic Research Laboratories, Cambridge, UK, (4) Oxford Centre for Diabetes, Endocrinology and Metabolism (OCDEM), University of Oxford, UK, (5) Wellcome Trust Centre for Human Genetics, University of Oxford, Oxford, UK, (6) Oxford NIHR Biomedical Research Centre, Oxford, UK, (7) Public Health Division of Gipuzkoa, San Sebastian, Spain, (8) Instituto BIO-Donostia, Basque Government, San Sebastian, Spain, (9) CIBER Epidemiología y Salud Pública (CIBERESP), Spain, (10) Inserm, CESP, U1018, Villejuif, France, (11) Univ Paris-Sud, UMRS 1018, Villejuif, France, (12) Navarre Public Health Institute (ISPN), Pamplona, Spain, (13) German Institute of Human Nutrition Potsdam-Rehbruecke, Germany, (14) Lund University, Malmö, Sweden, (15) Umeå University, Umeå, Sweden, (16) Catalan Institute of Oncology (ICO), Barcelona, Spain, (17) Epidemiology and Prevention Unit, Milan, Italy, (18) German Cancer Research Centre (DKFZ), Heidelberg, Germany, (19) University of Oxford, United Kingdom, (20) Department of Epidemiology, Murcia Regional Health Council, Murcia, Spain, (21) Unit of Preventive Medicine and Public Health, School of Medicine, University of Murcia, Spain, (22) Department of Public Health, Section for Epidemiology, Aarhus University, Aarhus, Denmark, (23) Aalborg University Hospital, Aalborg, Denmark, (24) Cancer Research and Prevention Institute (ISPO), Florence, Italy, (25) Dipartimento di Medicina Clinica e Chirurgia, Federico II University, Naples, Italy, (26) Public Health Directorate, Asturias, Spain, (27) Unit of Cancer Epidemiology, Citta' della Salute e della Scienza Hospital-University of Turin and Center for Cancer Prevention (CPO), Torino, Italy, (28) Human Genetics Foundation (HuGeF), Torino, Italy, (29) Andalusian School of Public Health, Granada, Spain, (30) Instituto de Investigación Biosanitaria de Granada (Granada.ibs), Granada (Spain), (31) International Agency for Research on Cancer, Lyon, France, (32) Danish Cancer Society Research Center, Copenhagen, Denmark, (33) ASP Ragusa, Italy, (34) Aire Onlus, Ragusa, Italy, (35) National Institute for Public Health and the Environment (RIVM), Bilthoven, The Netherlands, (36) University Medical Center Utrecht, Utrecht, the Netherlands, (37) School of Public Health, Imperial College London, UK

SUPPLEMENTARY NOTE 2. CHARGE T2D-Glycemia Exome Consortium Study Descriptions

Age, Gene/Environment, Susceptibility—Reykjavik (AGES) Study
The AGES study has been described previously. The study was initiated in 2002 to examine genetic susceptibility and gene/environment interactions related to disease and disability in old age. The AGES study is comprised of 5,764 individuals drawn from the Reykjavik Study, a population-based cohort comprised of individuals born between 1907 and 1935 and followed since 1967 by the Icelandic Heart Association. 2983 individuals have ExomeChip genotypes[1].

Atherosclerosis Risk in Communities (ARIC) Study
The ARIC Study is a prospective cohort study of cardiovascular disease risk in four US communities[2]. Between 1987 and 1989, 7,082 men and 8,710 women aged 45–64 years were recruited from Forsyth County, North Carolina; Jackson, Mississippi (African Americans only); suburban Minneapolis, Minnesota; and Washington County, Maryland. The ARIC Study protocol was approved by the institutional review board of each participating university. After written informed consent was obtained, including that for genetic studies, participants underwent a baseline clinical examination (Visit 1) and four subsequent follow-up exams (Visits 2 – 5).

Coronary Artery Risk Development in young Adults (CARDIA)
The CARDIA study is a prospective, multi-center investigation of the natural history and etiology of cardiovascular disease in African Americans and whites 18-30 years of age at the time of initial examination. The initial examination included 5,115 participants selectively recruited to represent proportionate racial, gender, age, and education groups from four communities: Birmingham, AL; Chicago, IL; Minneapolis, MN; and Oakland, CA. Participants from the Birmingham, Chicago, and Minneapolis centers were recruited from the total community or from selected census tracts. Participants from the Oakland center were randomly recruited from the Kaiser-Permanente health plan membership. From the time of initiation of the study in 1985-1986, seven follow-up examinations have been conducted at years 2, 5, 7, 10, 15, 20, and 25.

The Chinese American Eye Study (CHES) is a population-based study designed to assess the prevalence and risk factors of visual impairment, diabetic retinopathy, age-related macular degeneration, lens opacities, glaucoma and myopia, and to determine potential unique genetic associations with ocular disease in 4,582 (79% participation) non-institutionalized Chinese Americans aged 50 years and older living in Monterey Park, in Los Angeles County. Household residence were determined eligible if they lived in Monterey Park, were 50 years or older, and self-reported Chinese Americans. Eligible participants were asked to complete an in-home questionnaire which included demographic and ocular history information. Participants were then invited to complete a comprehensive eye examination, including presenting and best-corrected visual acuity measurements, visual field, intraocular pressure, random glucose, Hba1c, lens grading, fundus photography and diabetes status.

Cardiovascular Health Study (CHS)
CHS is an NHLBI-funded observational study of risk factors for cardiovascular disease in adults 65 years or older[3]. Starting in 1989, and continuing through 1999, participants underwent annual extensive clinical examinations. Measurements included traditional risk factors such as blood pressure and lipids as well as measures of subclinical disease, including echocardiography of the heart, carotid ultrasound, and cranial magnetic-resonance imaging (MRI). At six month intervals between clinic visits, and once clinic visits ended, participants were contacted by phone to ascertain hospitalizations and health status. The main outcomes are coronary heart disease (CHD), angina, heart failure (HF), stroke, transient ischemic attack (TIA), claudication, and mortality. Participants continue to be followed for these events. Participants of either European or African-American Ancestry were included in this study and signed informed test for genetic testing.

CoLaus Study
The CoLaus study is a community-based study of 6188 European white subjects aged 35 ~75 years[4]. Participants were drawn from the CHUV University Hospital in Lausanne Switzerland and studied for cardiovascular and metabolic phenotypes.

CROATIA-Korcula (Korcula) Study
The CROATIA-Korcula study includes 969 Croatians between the ages of 18 and 98. The field work was performed in 2007 and 2008 in the eastern part of the island, targeting healthy volunteers from the town of Korcula and the villages of Lumbarda, Zrnovo and Racisce. Ethical approval was obtained from appropriate regulatory bodies in both Scotland and Croatia and participants gave informed consent prior to joining the study. After all quality control measures, 855 European individuals were successfully genotyped using the Illumina HumanExome BeadChip array[5].

European Prospective Investigation into Cancer and Nutrition (EPIC)-Potsdam Study
The European Prospective Investigation into Cancer and Nutrition (EPIC) – Potsdam recruited men aged 40 to 64 years and women aged 35 to 64 years from the general population in Potsdam and surrounding municipalities from 1994 – 1998[6] and is part of the multi-center prospective cohort study EPIC[7]. The baseline assessment included a blood collection, self-administered questionnaires for assessment of lifestyle, socio-demographic factors and dietary habits, a personal interview on lifestyle habits and medical history as well as a physical examination to measure among others anthropometric characteristics[8]. A total number of 27,548 individuals participated in the study. From all participants that provided blood samples (n=26,444), a subcohort sample of 2,500 individuals was drawn randomly for measurement of genetic and bio-markers. Genotyping was additionally performed in 795 incident type 2 diabetes cases. Incident type 2 diabetes cases (N=849, mean follow-up time =7 years) were identified by an active follow up procedure[9]. Self-reports of diabetes diagnosis, diabetes-relevant medication, or dietary treatment due to diabetes were verified by questionnaires mailed to the diagnosing physician. Only subjects with a verified type 2 diabetes diagnosis were included in the analysis. The EPIC-Potsdam study was approved by the Ethics

Committee of the Medical Association of the State of Brandenburg (Germany). All participants provided written, informed consent at study entry.

The Exeter Family Study of Childhood Health (EFSOCH)
The EFSOCH is a prospective study, set up to test the fetal insulin hypothesis, and to identify genetic polymorphisms that play a role in determining birthweight and early postnatal growth. We recruited 1,017 families from a postcode-defined area in central Exeter. Specific inclusion criteria were established to obtain a homogeneous, non-diabetic, UK Caucasian cohort. Detailed anthropometric measurements were taken from both parents at 28 weeks' gestation, and from their children at birth, 12 weeks, 1 year and 2 years of age. Insulin and other biochemical analysis were measured in fasting parental samples and an umbilical cord blood sample taken at delivery. Parental and offspring DNA were extracted to allow molecular genetic analysis of candidate genes implicated in fetal growth[10].

The Erasmus Rucphen Family (ERF) Study
The ERF Study is a family-based cohort study in a genetically isolated population in the southwest of the Netherlands. Approximately 3,200 individuals, spanning an age range from 18 to 86 years old, participated in the study. The cross-sectional examinations took place between 2002 and 2005. The rationale and study design have been described elsewhere[11,12].

Family Heart Study (FamHS)
The FamHS began in 1992 with the ascertainment of 1,200 European American families, half randomly sampled, and half selected because of an excess of coronary heart disease or risk factor abnormalities as compared with age- and sex-specific population rates[13]. The families, with approximately 6,000 individuals, were sampled on the basis of information on probands from four population-based parent studies: the Framingham Heart Study (Boston, MA), the Utah Family Tree Study (Salt Lake City, UT), and two Atherosclerosis Risk in Communities (ARIC) study centers (Minneapolis, MN, and Forsyth County, NC). Eight years later, study participants belonging to the largest pedigrees were invited for a second clinical exam (2002-04). Approximately 82% of the recruited subjects returned for the second visit and 275 newly eligible family members were also recruited. In addition, a sample of 633 family members including 153 T2D subjects from 215 African American families was recruited at an additional ARIC field center (Birmingham, AL).

FENLAND Study
The Fenland Study is an ongoing, population-based cohort study (started in 2005) designed to investigate the association between genetic and lifestyle environmental factors and the risk of obesity, insulin sensitivity, hyperglycemia and related metabolic traits in men and women aged 30 to 55 years. Potential volunteers were recruited from General Practice sampling frames in the Fenland, Ely and Cambridge areas of the Cambridgeshire Primary Care Trust in the UK. Exclusion criteria for the study were: prevalent diabetes, pregnant and lactating women, inability to participate due to terminal illness, psychotic illness, or inability to walk unaided. All participants had measurements

done at the MRC Epidemiology Unit Clinical Research Facilities in Ely, Wisbech and Cambridge. Participants attended after an overnight fast for a detailed clinical examination, and blood samples were collected. The Local Research Ethics Committee granted ethical approval for the study and all participants gave written informed consent.

Framingham Heart Study (FHS)
The FHS is a three generational prospective cohort that has been described in detail previously[14]. Individuals were initially recruited in 1948 in Framingham, USA to evaluate cardiovascular disease risk factors. The second generation cohort (5,124 offspring of the original cohort and their spouse) was recruited between 1971 and 1975. The third generation cohort (4,095 grandchildren of the original cohort) was collected between 2002 and 2005. 8,153 European-American individuals had good quality genotypes from the Illumina HumanExome BeadChip array.

First Myocardial Infarction in Northern Sweden 3 (FIA3) Study
FIA3 is a population-based study of myocardial infarction (MI) nested within the Northern Sweden Health and Disease Study, (NSHDS), a population-based cohort study from northern Sweden, which consists of sub cohorts: the Västerbotten Intervention Program (VIP) and the WHO's Multinational Monitoring of Trends and Determinants in Cardiovascular Disease (MONICA) Study in northern Sweden. Both VIP and MONICA are health examination programs for cardiovascular disease (CVD) and diabetes. Cases are identified through the MONICA study in northern Sweden and its MI incidence registry. For the current study 2657 cases were genotyped with Illumina HumanExome BeadChip 12 v1.1[15].

Gene-Lifestyle interactions And Complex traits Involved in Elevated disease Risk (GLACIER) Study
The Gene-Lifestyle interactions And Complex traits Involved in Elevated disease Risk (GLACIER) Study is nested within the Västerbotten Health Survey, which is part of the Northern Sweden Health and Disease Study, a population-based prospective cohort study from northern Sweden. A total of 1000 non-diabetic participants from the GLACIER Study were genotyped with Illumina HumanExome Beadchip 12 v1.1[15].

Generation Scotland: Scottish Family Health Study (GS:SFHS)
The Generation Scotland: Scottish Family Health Study is a collaboration between the Scottish Universities and the NHS, funded by the Chief Scientist Office of the Scottish Government. GS:SFHS is a family-based genetic epidemiology cohort with DNA, other biological samples (serum, urine and cryopreserved whole blood) and socio-demographic and clinical data from ~24,000 volunteers, aged 18-98 years, in ~7,000 family groups. Participants were recruited across Scotland, with some family members from further afield, from 2006 - 2011. Most (87%) participants were born in Scotland and 96% in the UK or Ireland. The cohort profile has been published. GS:SFHS operates under appropriate ethical approvals, and all participants gave written informed consent. After all quality control procedures had been carried out, 9955 participants of European ancestry were genotyped using the Illumina HumanExome BeadChip array[16].

The Johns Hopkins Genetic Study of Atherosclerosis Risk (GeneSTAR)
GeneSTAR is a longitudinal family-based study examining determinants of incident coronary artery disease, stroke, and vascular disease among apparently healthy first degree adult relatives of probands who were hospitalized in Baltimore, Maryland, with documented coronary disease prior to 60 years of age. African American and white family members who were 21-80 years of age were enrolled between 1983 and 2007, and have been followed at regular five-year intervals.

GoMAP (Genetic Overlap between Metabolic and Psychiatric traits)
The GOMAP study includes 2871 unrelated Greek nationals of European ancestry 18-98 years of age recruited from five different hospitals in Athens Greece between 2012 and 2014. The study aims to explore shared inherited risk factors for metabolic and psychiatric disease. Genome-wide association scan (GWAS) was applied to biological samples from 422 individuals with type 2 diabetes (T2D). Clinical and haematological/biochemical measurements, interview-based lifestyle and socio-demographic information, detailed anthropometric measurements as well as information on treatment of T2D volunteers were collected. The cohort included in the present work comprises candidates with T2D regularly followed up in the outpatient diabetes centers (more than three outpatient visits on separate days) and receiving a prescriptions for insulin or oral antidiabetic agents. For newly diagnosed T2D patients ascertainment was conducted according to the WHO criteria. Ethical permission was obtained from the appropriate regulatory bodies and all volunteers gave written informed consent.

Health, Aging, and Body Composition (Health ABC) Study
Health ABC is a longitudinal, prospective study that tracks functional limitation onset and progression in a cohort of high-functioning older men and women recruited from Memphis, TN and Pittsburgh, PA using Medicare records. Recruitment began in 1997-1998 when participants were between 70 and 79 years of age and entry into study was dependent on participants' ability to walk one-quarter mile and climb 10 steps without difficulty. At baseline, the Health ABC cohort included 3,075 men and women, including 552 African-American men and 729 African-American women. The goal of the study is to understand how change in body composition and weight-related health conditions contributed to and affected incident functional limitation. Clinic examinations were conducted at baseline and at years 2, 3, 4, 5, 6, 8, 10, 11, and 16. Follow-up interviews by telephone have been conducted every six months since study initiation.

Health2008
Health2008 is a population-based epidemiological study of general health, diabetes and cardiovascular disease comprising 771 participants. An oral glucose tolerance test was performed with measurement of plasma glucose and serum insulin at fasting and 30 and 120 min after glucose intake. Health2008 was conducted at the Research Centre for Prevention and Health in Glostrup, Denmark. Informed written consent was obtained from all study participants. The studies were conducted in accordance with the Declaration of Helsinki II and were approved by the local Ethical Committee.

Hellenic Isolated Cohorts (HELIC) Study

The HELIC (Hellenic Isolated Cohorts) MANOLIS (Minoan Isolates) and Pomak collections focus on the Cretan Mylopotamos villages and the Pomak villages in Greece, respectively. Recruitment of these population-based samples was primarily carried out at the village medical centres. The study includes biological sample collection for DNA extraction and lab-based blood measurements, and interview-based questionnaire filling. The phenotypes collected include anthropometric and biometric measurements, clinical evaluation data, biochemical and haematological profiles, self-reported medical history, demographic, socioeconomic and lifestyle information. Biochemical measurements were obtained using enzymatic colorimetric assays and included glucose (hexokinase method, insulin and ferritin were measured via chemiluminescence and C-reaction protein (CRP) through an immunoturbidimetric method. The study was approved by the appropriate institutional review board (IRB) and appropriate informed consent was obtained from human subjects.

INCIPE Study
The INCIPE study (Initiative on Nephropathy, of relevance to public health, which is Chronic, possibly in its Initial stages, and carries a Potential risk of major clinical End-points) aims to explore the prevalence of CKD and related cardiometabolic traits in Italy[17]. Six thousand and two hundred (6,200) individuals, all Caucasians, ≥40-years old by January 1, 2006, were randomly chosen from the lists of patients of 62 randomly selected general practitioners (GPs) based in four geographical areas in the Veneto region, NE Italy. After exclusion of pregnant or lactating women, a written informed consent was obtained from a total of 3,870 subjects (62%). Each participant filled a self-administered questionnaire on family and personal medical history, pharmacologic treatments, smoking habits etc. Participants attended after an overnight fast for a detailed clinical examination performed locally in four units by trained medical doctors and blood and urine samples were collected with a standardized protocol. All determinations were centralized (Hospital Trust of Verona, Central Laboratory, Verona, Italy). Patients were asked to refrain from smoking beginning from the night before. BP, waist circumference, body weight, and height were measured as in the NHANES study[18]. About 2,500 study participants underwent Illumina ExomeChip v.1.0 genotyping, 1,933 of them (1,749 non-T2D, 184 affected by T2D) had complete information to contribute to the present study.

Inter99
The Inter99 cohort is a randomized, non-pharmacological intervention study for the prevention of ischaemic heart disease, conducted on 6,784 randomly ascertained participants aged 30 to 60 years at the Research Centre for Prevention and Health in Glostrup, Denmark (ClinicalTrials.gov: NCT00289237). An oral glucose tolerance test was performed with measurement of plasma glucose and serum insulin at fasting and 30 and 120 min after glucose intake. Subsequently, 6,094 participants of Danish nationality and with available DNA were classified as having normal glucose tolerance (*n*=4,525), impaired fasting glycaemia (*n*=504), impaired glucose tolerance (*n*=693), screen-detected type 2 diabetes (*n*=253), or previously diagnosed type 2 diabetes (*n*=119) according to World Health Organization (WHO) 1999 criteria. Informed written consent was obtained from all study participants. The studies were conducted in

accordance with the Declaration of Helsinki II and were approved by the local Ethical Committee.

InterAct Consortium
The InterAct study[19] is a case-cohort study nested within the European Prospective Investigation into Cancer and Nutrition (EPIC) cohorts[7], and includes 12,403 incident cases of T2D and a subcohort of 16,154 individuals (including 778 randomly selected incident T2D cases). Up to 2,266 incident cases of T2D and 3,734 controls genotyped on the Illumina CoreExome chip were included in the current analysis.

IPM Bio*Me* Biobank
The Bio*Me* Biobank Program is an ongoing, prospective, hospital- and outpatient-based population research program operated by The Charles Bronfman Institute for Personalized Medicine (IPM) at Mount Sinai. Bio*Me* has enrolled over 26,500 participants between September 2007 and August 2013. Bio*Me* is an Electronic Medical Record (EMR)-linked biobank that integrates research data and clinical care information for consented patients at The Mount Sinai Medical Center, which serves diverse local communities of upper Manhattan with broad health disparities. IPM Bio*Me* populations include 25% of African American ancestry (AA), 36% of Hispanic Latino ancestry (HL), 30% of white European ancestry (EA), and 9% of other ancestry. The IPM Bio*Me* disease burden is reflective of health disparities in the local communities. Bio*Me* operations are fully integrated in clinical care processes, including direct recruitment from clinical sites waiting areas and phlebotomy stations by dedicated Bio*Me* recruiters independent of clinical care providers, prior to or following a clinician standard of care visit. Recruitment currently occurs at a broad spectrum of over 30 clinical care sites. Analyses of Bio*Me* data was supported in part through the computational resources and staff expertise provided by the Department of Scientific Computing at the Icahn School of Medicine at Mount Sinai.

Insulin Resistance Atherosclerosis Study (IRAS)
IRAS was an epidemiologic cohort study designed to examine the relationship between insulin resistance and carotid atherosclerosis across a range of glucose tolerance. Individuals of self-reported African-American ethnicity were recruited in Oakland, CA and Los Angeles, CA. Recruitment was balanced across age and glucose tolerance status. The overall cohort consisted of 464 African-American individuals.

The Insulin Resistance Atherosclerosis Study Family Study (IRASFS)
IRASFS was a family study designed to examine the genetic and epidemiologic basis of glucose homeostasis traits and abdominal adiposity. Self-reported Mexican-American pedigrees were recruited in San Antonio, TX and San Luis Valley, CO. Probands with large families were recruited from the initial non-family-based IRAS Study. The overall cohort consisted of 1,414 Mexican-American individuals from 90 families and 596 African-American individuals from 42 families.

Jackson Heart Study (JHS) Study Description

The Jackson Heart Study is a community-based observational study of African Americans in the Jackson, MS, metropolitan area. Beginning in 2000 5,301 participants (mean age 54.9 ± 12.9) were enrolled to explore the causes of the large burden of common complex diseases in this population. Participants have been evaluated during three clinic examinations (2000-2004, 2004-2008, and 2009-2012). ExomeChip genotyping has been performed for 2,790 participants[20].

LOLIPOP Study
A population-based study of 21,915 subjects, primarily of Indian Asians and Northwestern Europeans aged 35–75 years, identified from the lists of 58 general practitioners in West London[21]. 549 European whites with exome sequencing data available from the GSK discovery sequence project were included in the current study.

Metabolic Syndrome GEMS Study
The GEMS study is a large multinational study designed to explore the genetic basis of the metabolic syndrome control[22]. Subjects were recruited from two centers in Europe (Oulu, Finland and Lausanne, Switzerland), one in the United States (Dallas, TX), one in Canada (Ottawa, Ontario), and one in Australia (Adelaide, South Australia). Dyslipidemic subjects were required to have the combination of an elevated plasma triglyceride (greater than 75th percentile) and a low serum HDL-cholesterol (less than 25th percentile) for their age, sex and country threshold (age 18-75 years) and were non-diabetic. Unrelated normolipidemic controls were required to have plasma triglyceride lower than 50th percentile, serum HDL-cholesterol greater than 50th percentile for their age, sex and country threshold, body mass index (BMI) greater than 25 kg/m2, and be greater than 40 years of age. Dyslipidemic subjects (n=787 subjects) and normolipidemic controls (n=792 subjects), matched by sex, age and collection center were sequenced in the GSK discovery sequence project and included in the current study.

METSIM (METabolic Syndrome In Men) Study
The METSIM Study includes 10,197 men, aged from 45 to 73 years at recruitment, randomly selected from the population register of the Kuopio town, Eastern Finland, and examined in 2005-2010[23]. Study protocol included interview on cardiovascular risk factors, measurement of height, weight, waist, hip, blood pressure, and bioimpedance for the evaluation of fat percentage. Laboratory studies include an oral glucose tolerance test to evaluate glucose tolerance (samples for glucose and insulin at 0, 30, and 120 minutes), as well as fasting laboratory measurements including lipids, lipoproteins, inflammatory markers, etc.

MRC Ely
The MRC Ely Study is a population-based cohort randomly selected from people living in Ely and surrounding villages (East Anglia, UK), an ethnically homogenous European ancestry population. The study design, methods and measurements of the three phases have been described in detail elsewhere. The current analyses included individuals aged 35-79 years, from phase 3. Ethical permission was granted by the Cambridgeshire

Research Ethics Committee, and study participants provided written informed consent. Data from 1394 individuals were included in the current analyses.

Multi-Ethnic Study of Atherosclerosis (MESA) Study

Three components of the Multi-Ethnic Study of Atherosclerosis (MESA, MESA Family, and MESA Air) underwent genotyping with the Exome chip as part of the MESA SNP Health Association Resource (SHARe). The parent MESA study is a National Heart, Lung and Blood Institute-sponsored, population-based investigation of subclinical cardiovascular disease and its progression[24]. In brief, a total of 6,814 individuals, aged 45 to 84 years, were recruited from six US communities (Baltimore City and County, MD; Chicago, IL; Forsyth County, NC; Los Angeles County, CA; New York, NY; and St. Paul, MN) between July 2000 and August 2002. Thirty-eight percent of the recruited participants are white, 28 percent African-American, 22 percent Hispanic, and 12 percent Asian (predominantly of Chinese descent). In MESA Family, 2,128 additional individuals from 594 African-American and Hispanic-American families were recruited utilizing the existing MESA framework. MESA Air, a study of air pollution exposure and cardiovascular disease, primarily consists of participants from MESA and MESA Family, plus 257 subjects recruited specifically for MESA Air from Los Angeles and New York. For the current study, there were 2456 white and 1549 African-American individuals with fasting glucose or fasting insulin and Exome chip data available for analysis.

Precocious Coronary Artery Disease (PROCARDIS) Study

The PROCARDIS (Precocious Coronary Artery Disease) Study recruited subjects with coronary artery disease at/before 65 years. Control subjects free of CAD before 65 years, were recruited from the same centers as cases. Ethics approval was granted by the each recruitment center (Sweden, UK, Germany, Italy). All participants completed a questionnaire including baseline characteristics, lifestyle, cardiovascular risk factors and medication. Standard biochemical phenotyping was performed[25].

Relationship between Insulin Sensitivity and Cardiovascular disease (RISC) Study

The RISC (Relationship between Insulin Sensitivity and Cardiovascular disease) Study is being carried out in 19 European recruiting centers to examine whether insulin sensitivity (directly measured with the euglycemic clamp technique) predicts cardiovascular disease (CVD) independently of other factors. The study makes use of ultrasound scans of the carotid artery and takes the thickness of the intima-media layer in the artery wall (IMT) as an early marker of atherosclerosis[26].

Rotterdam Study

The Rotterdam Study is an ongoing prospective population-based cohort study, focused on chronic disabling conditions of the elderly. The study comprises an outbred ethnically homogenous population of Dutch Caucasian origin. The rationale of the study has been described in detail elsewhere[27]. In summary, 7,983 men and women aged 55 years or older, living in Ommoord, a suburb of Rotterdam, the Netherlands, were invited to participate. A total of 2986 individuals from the initial study were included in the current study[27,28].

Stockholm Coronary Artery Risk Factor (SCARF) Study
Subjects <60 years admitted to 3 coronary care units in Stockholm (Danderyd Hospital, Karolinska Hospital and Norrtälje Hospital) for first MI were included in this study (n=387). Healthy age, county and sex-matched control were also recruited for each case. Blood sampling was carried out 3 months after the cardiac event at a medical examination under fasting conditions. In addition, an interview of social-economic factors, lifestyle and medical history was completed. Ethics approval was granted by the ethics committee at Karolinska[29].

Singapore Chinese Eye (SCES) Study
SCES is a population-based cross-sectional study of eye diseases in Chinese adults 40 years of age or older residing in the south-western part of Singapore. The study was designed to ascertain the prevalence and impact of major eye disease in Chinese persons in Singapore. An age-stratified (by 10-year age group) random sampling strategy was used to select participants from a computer-generated list provided by the Ministry of Home Affairs, Singapore. Between 2009 and 2011, 3,353 (72.8%) of 4,605 eligible individuals underwent a comprehensive physical and ophthalmologic examinations. Of these 2492 had ExomeChip data. The study adhered to the tenets of the Declaration of Helsinki, and ethical approval was obtained from the Institutional Review Board of the Singapore Eye Research Institute. All participants provided written informed consent.

Taiwan US Diabetic Retinopathy (TUDR) Study
Taiwan US Diabetic Retinopathy (TUDR) is a cohort that enrolled subjects with Type 2 diabetes receiving care at Taichung Veteran General Hospital (Taichung VGH), and a small number of subjects from Taipei Tri-Service General Hospital. All TUDR subjects underwent a complete ophthalmic and fundus examination to carefully document the presence and extent of retinopathy.

TEENAGE (TEENs of Attica: Genes and Environment)
The TEENAGE study comprises 857 adolescents of Greek origin randomly recruited from public secondary schools located in the wider Athens area of Attica in Greece[30].

ULSAM
All men born between 1920 and 1924 in Uppsala, Sweden were invited to participate at age 50 in this longitudinal cohort study that was started in 1970. Participants were reinvestigated at the ages of 60, 70, 77, 82 and 88 years.1 Blood samples for DNA extraction and main cardiovascular risk factors were available from the investigation at age 70 (n=1,146 with DNA and data on CVD risk factors). The participants have undergone extensive phenotyping at repeated time points, including euglycemic clamps, oral glucose tolerance tests, DXA, echocardiography, 24-h ambulatory blood pressure measurement, and a range of biomarkers.

Val Borbera (INGI-VB) Study
The INGI-Val Borbera population is a collection of 1803 samples recruited in the Val Borbera Valley, a geographically isolated valley located within the Appennine Mountains

in Northwest Italy [31,32]. The valley is inhabited by about 3,000 descendants from the original population, living in 7 villages along the valley and in the mountains. Participants were healthy people 18-102 years of age, 54% females having at least one grandfather living in the valley. Initial recruitment was in 2005-2008.

WGHS

The Women's Genome Health Study (WGHS, n=23,294) is a prospective cohort of initially healthy, female North American health care professionals at least 45 years old at baseline representing a subset of participants in the Women's Health Study (WHS) who provided a blood sample at baseline and consent for blood-based analyses. The WHS was a 2x2 trial beginning in 1992-1994 of vitamin E and low dose aspirin in prevention of cancer and cardiovascular disease with about 10 years of follow-up. Since the end of the trial, follow-up has continued in observational mode. Additional information related to health and lifestyle were collected by questionnaire throughout the WHS trial and continuing observational follow-up[33].

Supplementary REFERENCES

1. Harris, T.B. *et al.* Age, Gene/Environment Susceptibility-Reykjavik Study: multidisciplinary applied phenomics. *Am J Epidemiol* **165**, 1076-87 (2007).
2. The Atherosclerosis Risk in Communities (ARIC) Study: design and objectives. The ARIC investigators. *Am J Epidemiol* **129**, 687-702 (1989).
3. Fried, L.P. *et al.* The Cardiovascular Health Study: design and rationale. *Ann Epidemiol* **1**, 263-76 (1991).
4. Firmann, M. *et al.* The CoLaus study: a population-based study to investigate the epidemiology and genetic determinants of cardiovascular risk factors and metabolic syndrome. *BMC Cardiovasc Disord* **8**, 6 (2008).
5. Zemunik, T. *et al.* Genome-wide association study of biochemical traits in Korcula Island, Croatia. *Croat Med J* **50**, 23-33 (2009).
6. Boeing, H., Korfmann, A. & Bergmann, M.M. Recruitment procedures of EPIC-Germany. European Investigation into Cancer and Nutrition. *Ann Nutr Metab* **43**, 205-15 (1999).
7. Riboli, E. *et al.* European Prospective Investigation into Cancer and Nutrition (EPIC): study populations and data collection. *Public Health Nutr* **5**, 1113-24 (2002).
8. Boeing, H., Wahrendorf, J. & Becker, N. EPIC-Germany--A source for studies into diet and risk of chronic diseases. European Investigation into Cancer and Nutrition. *Ann Nutr Metab* **43**, 195-204 (1999).
9. Bergmann, M.M., Bussas, U. & Boeing, H. Follow-up procedures in EPIC-Germany--data quality aspects. European Prospective Investigation into Cancer and Nutrition. *Ann Nutr Metab* **43**, 225-34 (1999).
10. Knight, B., Shields, B.M. & Hattersley, A.T. The Exeter Family Study of Childhood Health (EFSOCH): study protocol and methodology. *Paediatr Perinat Epidemiol* **20**, 172-9 (2006).
11. Aulchenko, Y.S. *et al.* Linkage disequilibrium in young genetically isolated Dutch population. *Eur J Hum Genet* **12**, 527-34 (2004).

12. Pardo, L.M., MacKay, I., Oostra, B., van Duijn, C.M. & Aulchenko, Y.S. The effect of genetic drift in a young genetically isolated population. *Ann Hum Genet* **69**, 288-95 (2005).
13. Higgins, M. *et al.* NHLBI Family Heart Study: objectives and design. *Am J Epidemiol* **143**, 1219-28 (1996).
14. Kannel, W.B., Dawber, T.R., Kagan, A., Revotskie, N. & Stokes, J., 3rd. Factors of risk in the development of coronary heart disease--six year follow-up experience. The Framingham Study. *Ann Intern Med* **55**, 33-50 (1961).
15. Hallmans, G. *et al.* Cardiovascular disease and diabetes in the Northern Sweden Health and Disease Study Cohort - evaluation of risk factors and their interactions. *Scand J Public Health Suppl* **61**, 18-24 (2003).
16. Ng, B.G. *et al.* Mosaicism of the UDP-galactose transporter SLC35A2 causes a congenital disorder of glycosylation. *Am J Hum Genet* **92**, 632-6 (2013).
17. Ferraro, P.M. *et al.* Metabolic syndrome, cardiovascular disease, and risk for chronic kidney disease in an Italian cohort: analysis of the INCIPE study. *Metab Syndr Relat Disord* **9**, 381-8 (2011).
18. Gambaro, G. *et al.* Prevalence of CKD in northeastern Italy: results of the INCIPE study and comparison with NHANES. *Clin J Am Soc Nephrol* **5**, 1946-53 (2010).
19. InterAct, C. *et al.* Design and cohort description of the InterAct Project: an examination of the interaction of genetic and lifestyle factors on the incidence of type 2 diabetes in the EPIC Study. *Diabetologia* **54**, 2272-82 (2011).
20. Taylor, H.A., Jr. *et al.* Toward resolution of cardiovascular health disparities in African Americans: design and methods of the Jackson Heart Study. *Ethn Dis* **15**, S6-4-17 (2005).
21. Kooner, J.S. *et al.* Genome-wide scan identifies variation in MLXIPL associated with plasma triglycerides. *Nat Genet* **40**, 149-51 (2008).
22. Wyszynski, D.F. *et al.* Relation between atherogenic dyslipidemia and the Adult Treatment Program-III definition of metabolic syndrome (Genetic Epidemiology of Metabolic Syndrome Project). *Am J Cardiol* **95**, 194-8 (2005).
23. Stancakova, A. *et al.* Changes in insulin sensitivity and insulin release in relation to glycemia and glucose tolerance in 6,414 Finnish men. *Diabetes* **58**, 1212-21 (2009).
24. Bild, D.E. *et al.* Multi-ethnic study of atherosclerosis: objectives and design. *Am J Epidemiol* **156**, 871-81 (2002).
25. Farrall, M. *et al.* Genome-wide mapping of susceptibility to coronary artery disease identifies a novel replicated locus on chromosome 17. *PLoS Genet* **2**, e72 (2006).
26. Hills, S.A. *et al.* The EGIR-RISC STUDY (The European group for the study of insulin resistance: relationship between insulin sensitivity and cardiovascular disease risk): I. Methodology and objectives. *Diabetologia* **47**, 566-70 (2004).
27. Hofman, A., Grobbee, D.E., de Jong, P.T. & van den Ouweland, F.A. Determinants of disease and disability in the elderly: the Rotterdam Elderly Study. *Eur J Epidemiol* **7**, 403-22 (1991).
28. Hofman, A. *et al.* The Rotterdam Study: 2014 objectives and design update. *Eur J Epidemiol* **28**, 889-926 (2013).

29. Samnegard, A. *et al.* Serum matrix metalloproteinase-3 concentration is influenced by MMP-3 -1612 5A/6A promoter genotype and associated with myocardial infarction. *J Intern Med* **258**, 411-9 (2005).
30. Ntalla, I. *et al.* Body composition and eating behaviours in relation to dieting involvement in a sample of urban Greek adolescents from the TEENAGE (TEENs of Attica: Genes & Environment) study. *Public Health Nutr* **17**, 561-8 (2014).
31. Traglia, M. *et al.* Heritability and demographic analyses in the large isolated population of Val Borbera suggest advantages in mapping complex traits genes. *PLoS One* **4**, e7554 (2009).
32. Colonna, V. *et al.* Small effective population size and genetic homogeneity in the Val Borbera isolate. *Eur J Hum Genet* **21**, 89-94 (2013).
33. Ridker, P.M. *et al.* Rationale, design, and methodology of the Women's Genome Health Study: a genome-wide association study of more than 25,000 initially healthy american women. *Clin Chem* **54**, 249-55 (2008).
